# Hybridization and polyploidy enable genomic plasticity without sex in the most devastating plant-parasitic nematodes

**DOI:** 10.1371/journal.pgen.1006777

**Published:** 2017-06-08

**Authors:** Romain Blanc-Mathieu, Laetitia Perfus-Barbeoch, Jean-Marc Aury, Martine Da Rocha, Jérôme Gouzy, Erika Sallet, Cristina Martin-Jimenez, Marc Bailly-Bechet, Philippe Castagnone-Sereno, Jean-François Flot, Djampa K. Kozlowski, Julie Cazareth, Arnaud Couloux, Corinne Da Silva, Julie Guy, Yu-Jin Kim-Jo, Corinne Rancurel, Thomas Schiex, Pierre Abad, Patrick Wincker, Etienne G. J. Danchin

**Affiliations:** 1 INRA, Université Côte d’Azur, CNRS, ISA, France; 2 Bioinformatics Center, Institute for Chemical Research, Kyoto University, Gokasho, Uji, Kyoto, Japan; 3 Commissariat à l’Energie Atomique (CEA), Institut de Génomique (IG), Genoscope, Evry, France; 4 LIPM, Université de Toulouse, INRA, CNRS, Castanet-Tolosan, France; 5 Université Libre de Bruxelles (ULB), Evolutionary Biology & Ecology, Brussels, Belgium; 6 CNRS, Université Côte d’Azur, Institute of Molecular and Cellular Pharmacology, France; 7 MIAT, Université de Toulouse, INRA, Castanet Tolosan, France; 8 Université d’Evry Val d’Essonne, UMR 8030, Evry, France; 9 Centre National de Recherche Scientifique (CNRS), UMR 8030, Evry, France; National Institute of Genetics, JAPAN

## Abstract

Root-knot nematodes (genus *Meloidogyne*) exhibit a diversity of reproductive modes ranging from obligatory sexual to fully asexual reproduction. Intriguingly, the most widespread and devastating species to global agriculture are those that reproduce asexually, without meiosis. To disentangle this surprising parasitic success despite the absence of sex and genetic exchanges, we have sequenced and assembled the genomes of three obligatory ameiotic and asexual *Meloidogyne*. We have compared them to those of relatives able to perform meiosis and sexual reproduction. We show that the genomes of ameiotic asexual *Meloidogyne* are large, polyploid and made of duplicated regions with a high within-species average nucleotide divergence of ~8%. Phylogenomic analysis of the genes present in these duplicated regions suggests that they originated from multiple hybridization events and are thus homoeologs. We found that up to 22% of homoeologous gene pairs were under positive selection and these genes covered a wide spectrum of predicted functional categories. To biologically assess functional divergence, we compared expression patterns of homoeologous gene pairs across developmental life stages using an RNAseq approach in the most economically important asexually-reproducing nematode. We showed that >60% of homoeologous gene pairs display diverged expression patterns. These results suggest a substantial functional impact of the genome structure. Contrasting with high within-species nuclear genome divergence, mitochondrial genome divergence between the three ameiotic asexuals was very low, signifying that these putative hybrids share a recent common maternal ancestor. Transposable elements (TE) cover a ~1.7 times higher proportion of the genomes of the ameiotic asexual *Meloidogyne* compared to the sexual relative and might also participate in their plasticity. The intriguing parasitic success of asexually-reproducing *Meloidogyne* species could be partly explained by their TE-rich composite genomes, resulting from allopolyploidization events, and promoting plasticity and functional divergence between gene copies in the absence of sex and meiosis.

## Author summary

Sexual reproduction is evolutionary successful in animals as more than 99% of species reproduce sexually. The few animals that have abandoned sex are usually believed to be short-lived and outcompeted by their sexual relatives. Yet, in the root-knot nematodes, plant pests of global economic importance, an intriguing feature is observed. Species that have abandoned sex cause more damage and have a wider worldwide geographic distribution than their sexual cousins. To understand this puzzling success without sex, we have sequenced and analyzed the genomes of the three most devastating asexual nematodes and compared them to that of a sexual relative. We showed that their genomes are large, duplicated, transposon-rich and have hybrid origins. Due to this polylploid hybrid origin, most of their genes are in several copies with substantial sequence divergence. We detected signs of positive selection between these gene copies and confirmed functional divergence at the expression pattern level. We hypothesize that their peculiar hybrid genome structures provide these nematodes with a potential for adaptation and plasticity and could explain their paradoxical success in the absence of sex.

## Introduction

Fully asexual reproduction occurs in only ~0.1% of animal lineages, which generally occupy shallow branches in the tree of life [1,2]. Although there are some exceptions [3–5], the majority of asexual lineages of animals seem to be recently derived from sexual lineages, suggesting they are generally short-lived. Asexual animals lack the possibility to combine advantageous alleles from different individuals *via* sexual recombination and in association with Hill-Robertson effect and linkage between conflicting alleles, selection is assumed to be less efficient [6,7]. Furthermore, Muller's ratchet [8] and Kondrashov’s hatchet [9] models of “clonal decay” predict that they progressively accumulate deleterious mutations. Supporting these models, different studies have demonstrated accelerated accumulation of harmful mutations in asexual lineages [10–14], or (short-term) increased accumulation of transposable elements (TE) in the absence of sex [15,16]. Hence, it is commonly postulated that obligate parthenogenetic animals have evolutionary and adaptive disadvantages compared to their sexual relatives and therefore represent evolutionary dead-ends. Consistent with the geographical parthenogenesis model, parthenogenetic populations of plant and animals are generally present at the edge of the geographical distribution of species, in marginal or anthropologically disturbed environments [17,18]. Their uniparental clonal reproductive mode is supposed to be advantageous for colonizing marginal environments where they escape competition with their sexual relatives. Indeed, parthenogenetic species are frequently found at higher latitudes and altitudes [17].

Root-knot nematodes (genus *Meloidogyne*) display a variety of reproductive modes ranging from sexual reproduction (amphimixis) to obligate asexual reproduction (apomixis) with intermediates able to alternate between sexual (amphimixis) and asexual (automixis) reproduction [19]. These notorious plant pests have been ranked number one in terms of economic threat to the agriculture among all nematodes [20]. Challenging the view that fully asexual lineages of animals are outcompeted by their sexual relatives, *Meloidogyne* species that reproduce without meiosis and without sex have a broader host range, a wider and more southern geographical distribution and are more devastating than their sexual relatives [21,22]. Whether some genomic singularities could account for their higher parasitic success despite the absence of sex remains unclear. In 2008, we coordinated the publication of the draft genome sequence of *Meloidogyne incognita* [23], an obligatory asexual nematode and the draft genome of *M*. *hapla*, a facultative sexual, was published the same year [24]. One singularity of the *M*. *incognita* genome was the presence of genomic regions in two or more copies that spanned several megabases and had an average nucleotide divergence of ~8% [23]. Such a structure was identified neither in the facultative sexual *M*. *hapla* nor in any nematode able to reproduce sexually, so far.

The possible origin of the duplicated and diverged genomic regions observed in *M*. *incognita* is still debated. Two main hypotheses for the origin of this duplicated genome structure are that (i) duplicated regions represent former paternal and maternal genomes that diverged and became rearranged after their diploid sexual ancestor became asexual or (ii) they result from interspecific hybridization events [21]. As early as 1983, observation of heterozygous patterns of isozymes had led to the hypothesis that *M*. *incognita* might have undergone hybridization [25]. Likewise, based on the presence of multiple divergent ITS nuclear markers within apomictic *Meloidogyne* despite closely related mitochondrial markers between species, it was suggested that these species had undergone hybridization from a set of closely related females with more diverse paternal lineages [26]. Recently, on the basis of the comparative analysis of the initial *M*. *incognita* genome and a draft of the meiotic asexual *M*. *floridensis* genome, it was also suggested that *M*. *incognita* is of hybrid origin [27].

Regardless of its origin, the potential functional impact conferred by the duplicated genome structure of *M*. *incognita* has never been assessed. Furthermore, in the absence of genomes for other apomictic *Meloidogyne*, it was impossible to state whether such a duplicated genome structure is a specificity of *M*. *incognita* or a more general signature of the most economically important root-knot nematodes that, intriguingly, all reproduce asexually.

Here, we aimed at characterizing the genome structures of asexual root-knot nematodes, their most likely origin and the potential consequences at the functional level. We have re-sequenced the genome of *M*. *incognita* at much deeper coverage and have sequenced *de novo* the genomes of *M*. *javanica* and *M*. *arenaria*, two other apomictic root-knot nematodes of high economic importance. We have assembled these three genomes and validated genome assembly sizes with experimental assays. We confirm that the genomes of *M*. *incognita* and of the two other mitotic asexual *Meloidogyne* are made of duplicated yet diverged and rearranged genome copies. We have annotated the protein-coding genes and TE of the three genomes and performed a comparative genomic analysis, including the genome of the facultative sexual species *M*. *hapla* and the meiotic parthenogenetic *M*. *floridensis*. We show that the genomes of asexual mitotic *Meloidogyne* have a higher abundance of TE than *M*. *hapla* and any other nematode genome published so far. Using a phylogenomic analysis of the duplicated genomic regions conserved between species, we deciphered the origin and evolutionary history of the peculiar genome architecture of mitotic asexual *Meloidogyne*. To assess the potential functional outcome of these duplicated regions at a whole-genome scale, we searched and found signs of positive selection between the gene copies defining these genomic blocks. Using RNAseq analysis of different life stages of *M*. *incognita*, we show that the majority of gene pairs forming duplicated blocks display diverged expression profiles. Furthermore, gene pairs detected as under positive selection show a significantly higher proportion of diverged expression profiles. Our results show that mitotic asexual *Meloidogyne* possess duplicated, highly diverged and TE-rich genomes, an ensemble of features unequaled in any other nematode genome so far. We propose that the peculiar genome structures of these nematodes offer potential for adaptive plasticity and might contribute to the paradoxical success of these plant-parasitic animals despite the absence of sex.

## Results

### Genome assemblies are large, experimentally supported and show high completeness

**Fig 1:**
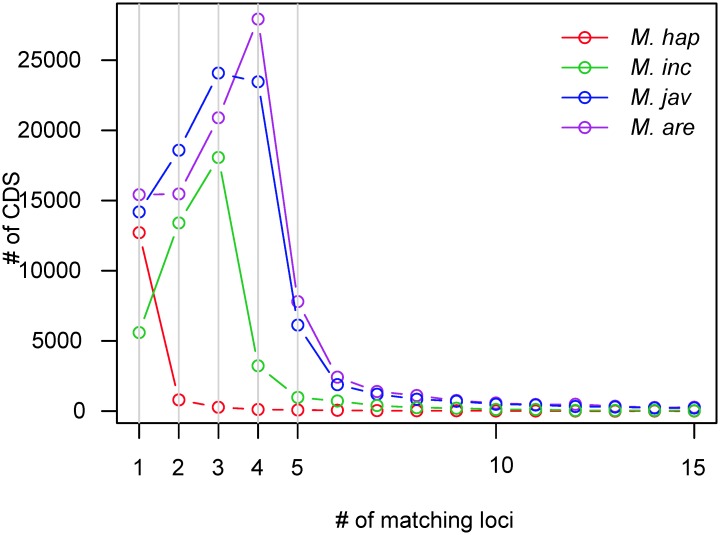
Distribution of CDS mapping one to several loci in the *Meloidogyne* genomes. Occurrences (y axis) of *Meloidogyne* CDS mapping at minimum 95% identity on minimum 2/3 of their length to one, two, three, four or up to 15 loci (x axis) in their respective genomes. In *M*. *hapla* (red), >89% of the CDS map to one single locus while >85% of the CDS map to multiple loci in the apomicts *M*. *incognita* (green), *M*. *javanica* (blue) and *M*. *arenaria* (violet).

We sequenced the genomes of three asexually reproducing *Meloidogyne* species and the assemblies reached 184, 236 and 258 Mb, for *M*. *incognita* (*Mi*), *M*. *javanica* (*Mj*) and *M*. *arenaria* (*Ma*), respectively (Table 1). These genome assemblies are bigger than any *Meloidogyne* genome assembly reported so far. To confirm these genome sizes, we measured DNA content *via* flow cytometry experiments and obtained size estimates of 189 ±15, 297 ±27 and 304 ±9 Mb for *Mi*, *Mj* and *Ma*, respectively. The genome assembly of *Mi* was in the range of estimated size *via* flow cytometry whereas *Mj* and *Ma* assemblies were smaller by 34–60 Mb. To check whether these differences in sizes could be explained by duplicated or repetitive regions collapsed during genome assembly, we plotted the distribution of read coverage along the genome (S1 Fig). We estimated that 17.1 (Mi), 42.9 (Mj) and 21.6 (Ma) Mb of genome assemblies have a coverage twice higher than the rest of the genome sequence and might represent collapsed duplicated regions. Hence, part of the differences in sizes can be explained by these collapsed regions, as previously observed in the genome of the obligate mitotic rotifer *Adineta vaga* [5]. The genome assemblies contained 97% (*Mi*), 96% (*Mj*) and 95% (*Ma*) of the 248 Core Eukaryotic Genes (CEG) in complete length [28]. These are the highest scores for a *Meloidogyne* genome so far, suggesting these assemblies are the most complete available to date. We annotated 45,351 (*Mi*), 98,578 (*Mj*) and 103,269 (*Ma*) genes (including, protein-coding genes, ncRNAs, rRNAs and tRNAs). Protein-coding genes spanned up to 43.7 (24%), 75.2 (32%) and 82.2 (32%) Mb of the *Mi*, *Mj* and *Ma* genomes, respectively. Because genome assemblies of the asexual *Meloidogyne* were ~3–5 times bigger than the haploid genome size of the facultative sexual *M*. *hapla*, we suspected these genomes to be polyploid. As a proxy to estimate ploidy levels, we mapped back all the protein-coding sequences (CDS) to their respective genome assemblies and analyzed the proportion of CDS mapping one locus or several loci in the genomes (Fig 1). In the facultative sexual *M*. *hapla*, we observed a peak of CDS mapping one single locus in the genome, indicating no sign of whole genome duplication (WGD). In contrast, in the mitotic asexuals, we observed peaks of CDS mapping 3, 3 to 4 and 4 loci in the genomes of *Mi*, *Mj* and *Ma*, respectively. These CDS mapping multiple loci are consistent with the genome sizes of the asexual *Meloidogyne* (3-5x bigger than the *M*. *hapla* genome) and support their polyploid nature.

**Table 1:** Size, assembly and gene annotation statistics of *Meloidogyne* genomes.

Species	*M*. incognita*	*M*. javanica*	*M*. arenaria*	*M*. *hapla*	*M*. *floridensis*
Flow cytometry* (Mb)	189 ±15	297 ±27	304 ±9	121 ±3	NA
Assembly size (Mb)	183.53	235.80	258.07	53.60	99.89
N50 (bp)	38,588	10,388	16,462	83,645	3,516
Complete CEG	97%	96%	95%	93%	60%
# genes**	45,351	98,578	103,001	NA	NA
# CDS	43,718	97,208	101,269	14,207	49,941
Protein-coding (Mb) (% assembly)	43.7 (23.8%)	75.2 (31.9%)	82.2 (32.1%)	NA	NA

* This study.

** Including non-protein-coding genes NA = information not available

Transposable elements (TE) covered 50.0% (Mi), 50.8% (Mj) and 50.8% (Ma) of the genome assemblies. In comparison, only 29.2% of the *M*. *hapla* genome was covered by TE, using the same annotation protocol (Table 2). Due to its high fragmentation state, the genome of *M*. *floridensis* could not be annotated for TE. On average, 27–30% of the genes of mitotic parthenogenetic species are included within TE, whereas this proportion reaches only 17% in *M*. *hapla* (see TE section for more details).

**Table 2:** Abundance and diversity of transposable elements in *Meloidogyne* genomes.

% genome / Species	*M*. *incognita*	*M*. *javanica*	*M*. *arenaria*	*M*. *hapla*
**Class I:**	**18.05**	**18.92**	**19.20**	**12.11**
LINE: (copy#)	1.57 (2067)	1.52 (3012)	1.53 (3214)	1.31 (513)
LTR: (copy#)	2.93 (3458)	3.49 (5831)	3.6 (6213)	1.84 (677)
PLE: (copy#)	0.03 (53)	0.04 (86)	0.03 (80)	0.05 (13)
SINE: (copy#)	<0.01 (30)	<0.01 (50)	<0.01 (37)	<0.01 (5)
DIRS: (copy#)	0.21 (221)	0.29 (412)	0.32 (460)	0.05 (17)
Uncl.: (copy#)	13.3 (25347)	13.57 (37451)	13.71 (39449)	8.86 (5025)
**Class II:**	**10.53**	**10.02**	**10.34**	**5.39**
Helitron: (copy#)	2.05 (1588)	2.13 (2774)	2.11 (2755)	1.77 (525)
Maverick: (copy#)	3.62 (100)	3.31 (121)	3.51 (139)	1.54 (2)
TIR: (copy#)	3.69 (7128)	3.53 (9675)	3.58 (10547)	1.66 (977)
Uncl.: (copy#)	1.17 (3577)	1.04 (4478)	1.13 (5169)	0.43 (363)
**Other: cov.**	21.41	21.82	21.24	11.65
**Total:**	**49.99**	**50.76**	**50.78**	**29.16**

### The genomes of asexual *Meloidogyne* are highly duplicated and gene copies form divergent syntenic blocks

Genome sizes as well as distribution of multi-mapping CDS strongly suggested polyploidy in the asexual *Meloidogyne* (see above). We used MCScanX [29] to further investigate the duplication relationships of the protein-coding genes in *Meloidogyne* genomes. MCScanX classifies protein-coding genes as (*i*) singleton when no duplicates are found in the assembly, (*ii*) proximal when duplicates are on the same scaffold and separated by 1 to 10 genes, (*iii*) tandem when duplicates are consecutive, (*iv*) WGD or segmental when duplicates form collinear blocks with other pairs of duplicated genes and (*v*) dispersed when the duplicates cannot be assigned to any of the other categories. In the three mitotic asexual *Meloidogyne* species, 93.0–94.1% of protein-coding genes were estimated to be duplicated whereas only 46.6% were duplicated in the facultative sexual *M*. *hapla* and 52.9% in the meiotic parthenogen *M*. *floridensis* (Table 3). We noted that the dispersed category was the most frequent in all *Meloidogyne* genomes. However, this proportion negatively correlated with N50 values in the mitotic *Meloidogyne*, suggesting that these duplicates might be re-classified in other categories in the future.

**Table 3:** MCScanX classification of protein-coding genes in *Meloidogyne* genome sequences.

Species	*M*. *incognita*	*M*. *javanica*	*M*. *arenaria*	*M*. *hapla*	*M*. *floridensis*
Total CDS	43,718	97,208	101,269	14,207	49,941
Singleton	3,043 (7.0%)	5,872 (6.0%)	5,937 (5.9%)	7,582 (53.4%)	23,513 (47.1%)
Dispersed	25,964 (59.4%)	78,989 (81.2%)	73,569 (72.7%)	4,891 (34.4%)	25,610 (51.3%)
Proximal	1,026 (2.4%)	2,116 (2.2%)	2,647 (2.6%)	791 (5.6%)	168 (0.3%)
Tandem	1,240 (2.8%)	4,425 (4.6%)	3,484 (3.4%)	853 (6.0%)	638 (1.3%)
WGD / segmental	12,445 (28.5%)	5,806 (6.0%)	15,632 (15.4%)	90 (0.6%)	12 (~0%)
N50 (kb)	38.6	10.4	16.5	83.6	3.5

Interestingly, 12,445, 5,806 and 15,632 genes were classified in the WGD / segmental category in *Mi*, *Mj* and *Ma*, respectively (Table 3). They formed 933 (*Mi*), 581 (*Mj*) and 1,648 (*Ma*) pairs of segmentally duplicated genome regions. In contrast, there were only 90 genes forming 11 pairs of duplicated regions in *M*. *hapla* and only 12 genes forming one pair of regions in *M*. *floridensis* (Table 4). Collinear duplicated regions span up to 58.6, 14.8 and 59.0 Mb of *Mi*, *Mj* and *Ma* genomes, corresponding to 31.8%, 6.3% and 23.0% of their respective sizes (Table 4). Average nucleotide divergence between pairs of duplicated regions was 8.4%, 7.5% and 8.2% for *Mi*, *Mj* and *Ma*, respectively, indicating a similar average divergence of ~8%. The distribution of % divergence between duplicated regions presented one single to two almost totally overlapping peaks (S2 Fig). This observation holds for the three apomictic *Meloidogyne* and suggests the duplication events have occurred in a same time window. The divergence levels were substantially lower in coding regions (4.7, 6.0 and 5.9%) than in non-coding regions (9.7, 9.0 and 9.7% for intergenic and 11, 10.4 and 11.1% for introns) for *Mi*, *Mj* and *Ma*, respectively (Table 4, S3 Fig).

**Table 4:** Number of pairs of duplicated regions, cumulative size and divergence.

Species		*M*. *incognita*	*M*. *javanica*	*M*. *arenaria*
# pairs of duplicated regions		933	581	1648
Cumulative size of duplicated collinear regions(Mb) (% genome sequence)		58.6 (31.8%)	14.8 (6.3%)	59.0 (23.0%)
% nucleotide divergence (standard deviation) computed from NUCmer alignments	Overall	8.4 (2.5)	7.5 (2.9)	8.2 (2.7)
	intergenic	9.7 (3.3)	9.0 (4.2)	9.7 (3.5)
	introns	11.0 (2.8)	10.4 (3.2)	11.1 (2.4)
	CDS	4.7 (1.7)	6.0 (2.7)	5.9 (2.4)
Median CDS % divergence (MCscanX)		3.5	3.8	4.1
Median protein % divergence (MCscanX)		2.7	3.7	4
Median Ks		0.1	0.1	0.1

Median rates of synonymous substitutions (Ks) between gene pairs forming duplicated regions were 0.1 for the three apomictic *Meloidogyne*. Averaged Ks for duplicated pairs of regions were significantly negatively correlated with collinearity (P<10^−8^ for *Mi*, *Mj* and *Ma*), which we measured as the fraction of collinear genes within a pair of regions (S4 Fig). This indicates that divergence in terms of number of conserved genes between a pair of regions correlates with the nucleotide divergence of the coding sequences.

**Fig 2:**
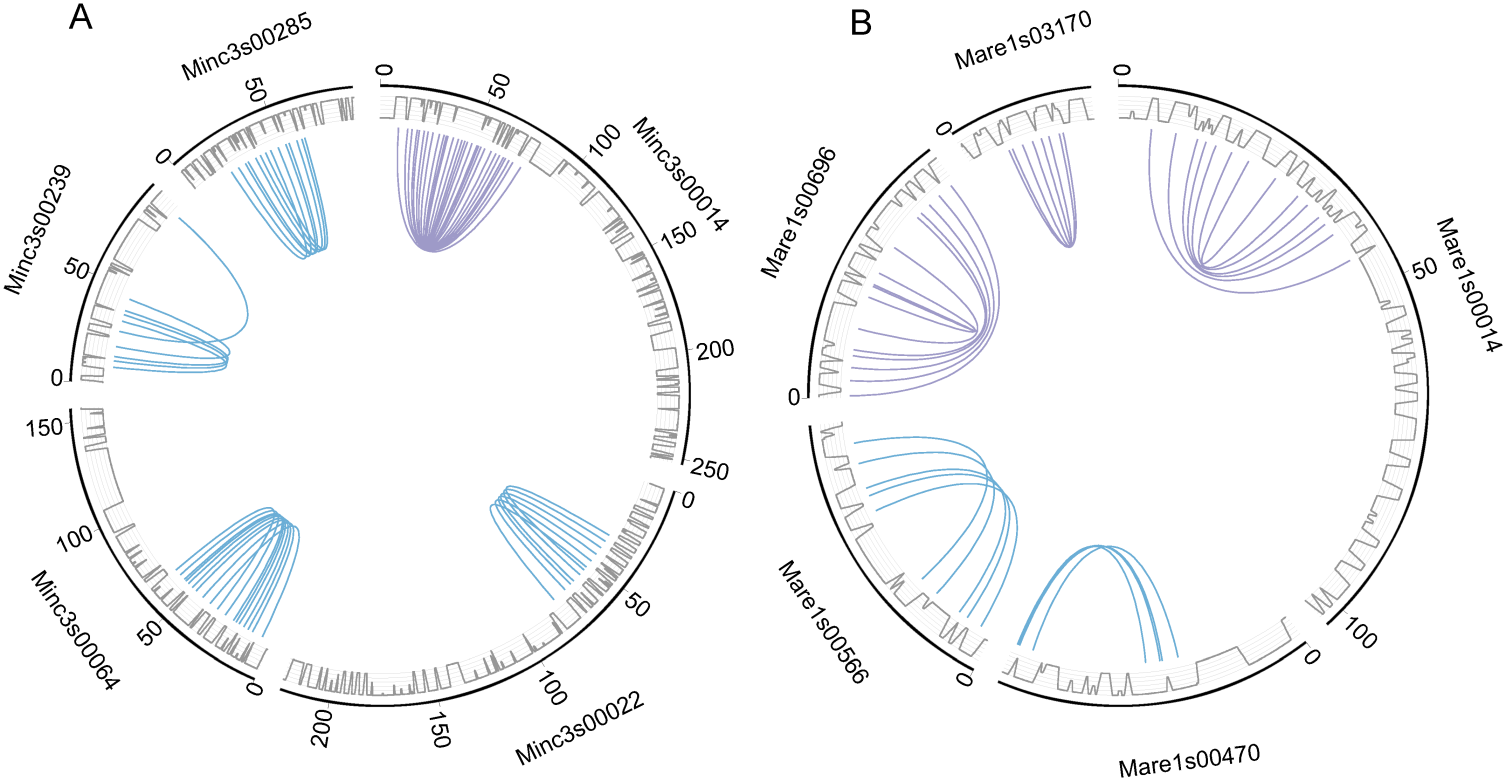
A genome structure consistent with absence of meiosis. Five pairs of duplicated homologous collinear regions co-occur on a same scaffold in *M*. *incognita* (A) and five in *M*. *arenaria* (B). All curves show connections between collinear gene pairs used by MCScanX to define duplicated regions (blue curves show tandem duplications and purple curves show palindromic duplications). Grey lines represent the gene density on the scaffolds.

In *M*. *incognita* and *M*. *arenaria*, we found 5 instances of duplicated regions on a same scaffold (Fig 2). In *M*. *incognita*, this corresponded to 42 collinear genes present in 4 pairs of tandem regions and 1 palindrome, whereas in *M*. *arenaria*, we found 29 collinear genes present in 2 pairs of tandem regions and 3 palindromes. If the duplicated regions represent vestiges of homologous chromosomes, such tandem and palindrome structures appear consistent with absence of chromosome pairing and meiosis, such as in the genome of *A*. *vaga* [5]. No similar structure was found in the genomes of *M*. *javanica*, *M*. *hapla* or *M*. *floridensis*. Average Ks value of gene pairs forming tandem or palindromic regions were in the range of Ks values measured for gene pairs in the rest of duplicated regions, suggesting they have the same divergence times.

We found that 29–37% of gene copies forming duplicated regions in mitotic *Meloidogyne* were TE-derived, a proportion comparable to the proportion observed for the rest of the whole gene sets (27–30%). Furthermore, the distribution of Ks for pairs of TE-derived genes is not significantly different from the distribution of the rest of pairs of genes in duplicated regions, according to a Wilcoxon test. Thus, we can rule out the possibility that the observed duplicated regions are the results of TE multiplications.

In plant genomes, following WGD, fractionation biases can be observed. One genomic copy tends to retain more genes and to accumulate less mutations than the other copy [30]. For each pair of duplicated regions in *Meloidogyne*, we tested whether a bias of retention of ancestral genes could be observed (Methods). We found 24 (*Mi*), 1 (*Mj*) and 36 (*Ma*) cases where one region had significantly (Chi-square test, 5% level) retained more ancestral genes than its counterpart. By comparing all the genes in duplicated regions and the MCScanX classification of gene copies, we also estimated that only 6%, 4% and 5% of ancestral collinear genes had no more copies anywhere in the *Mi*, *Mj* and *Ma* genomes, respectively and had probably been lost after WGD.

### The duplicated regions have different origins and evolutionary histories

**Fig 3:**
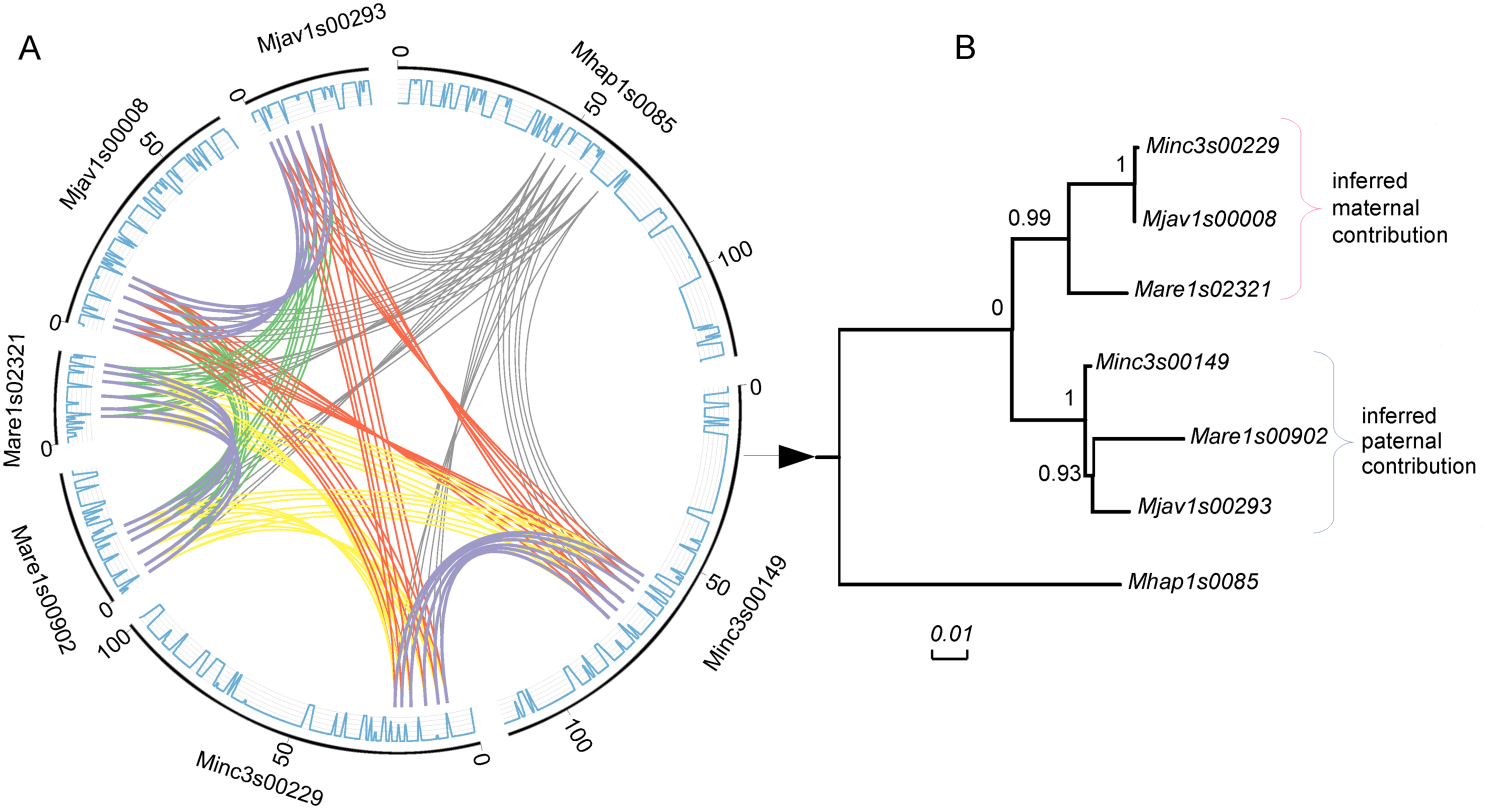
Example of structural and evolutionary relationship between pairs of duplicated regions. A. Circos [31] plot showing the collinear gene pairs (forming homologous regions) that were used for phylogenetic analyses (units = kb). All curves show the connections between the collinear gene pairs used by MCScanX to define segmental duplications. In each Circos plot, color codes are as follows. Collinear orthologs between *M*. *hapla* and any of the three asexuals species are in grey. Collinear ‘homoeologs’ within asexual species are in purple. Collinear orthologs between *M*. *arenaria* and *M*. *javanica* are in green. Collinear orthologs between *M*. *arenaria* and *M*. *incognita* are in yellow. Collinear orthologs between *M*. *incognita* and *M*. *javanica* are in red. The outer blue lines represent the gene density on the scaffolds. B. Maximum-likelihood phylogeny of concatenated alignments of collinear protein-coding genes used to form blocks with SH-like branch support. Topologies identical to the mitochondrial phylogeny were considered to represent the maternal contribution to the nuclear genome. The other topologies were considered as representative of paternal contributions.

To decipher the evolutionary history of the duplicated structure observed in the mitotic *Meloidogyne*, we conducted a phylogenomic analysis focused on the gene copies forming pairs of duplicated regions. We identified and used a dataset composed of 60 groups of homologous genomic regions defined as follows. The genomic regions must be conserved and contain at least 3 collinear genes in 2 copies in each of the mitotic *Meloidogyne* vs. one single copy in the amphimictic *M*. *hapla* (Fig 3 for an example). These 60 groups of genomic regions encompass 2,202 homologous genes distributed in 222 clusters (Fig 3A for an example, S5 Fig for the 60 conserved and duplicated homologous regions). Within each of the 60 groups of genomic regions, we generated multiple alignments of all the clusters of homologous genes individually. Although duplicated genes within a species are, on average, relatively distant (Ks = 0.1, 5–6% nucleotide divergence), gene copies between species can occasionally be identical. In order to maximize phylogenetic signal, the multiple alignments of each cluster of homologous genes were concatenated in each group of conserved duplicated regions. From the 60 concatenated multiple alignments, we successfully generated 54 maximum-likelihood (ML) phylogenies (6 failed because of short alignments, see Fig 3B for an example tree and S5 Fig for the 54 ML trees of conserved homologous regions).

**Fig 4:**
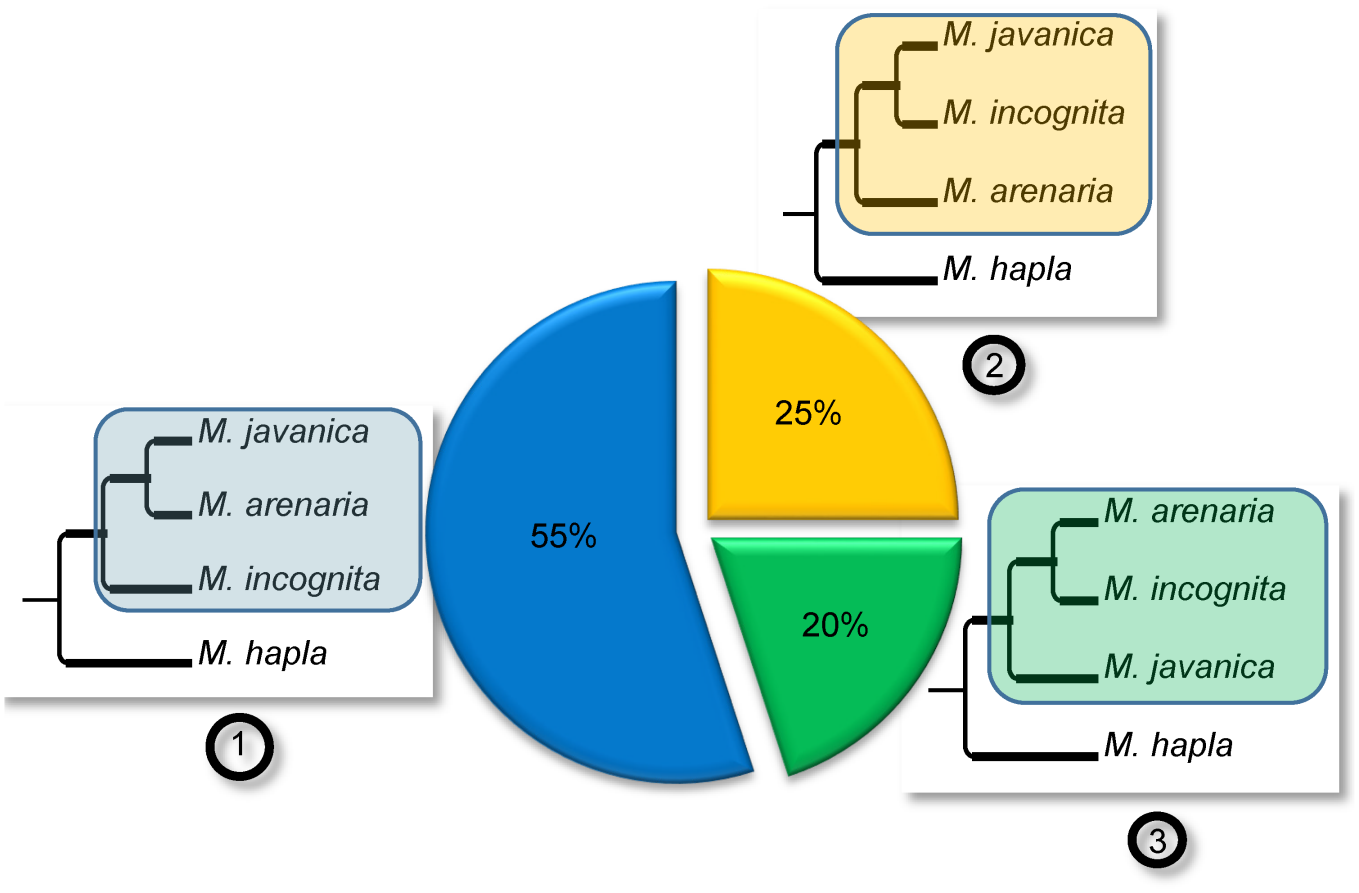
Phylogenetic relationships between duplicated regions in the genomes of apomictic *Meloidogyne*. The three possible topologies for bifurcating trees separating *M*. *incognita*, *M*. *javanica*, *M*. *arenaria* and their sexual relative *M*. *hapla* are represented as (1), (2) and (3) and their relative observed frequencies are indicated in the associated pie chart. The frequencies were calculated from the 40 phylogenetic trees containing at least one monophyletic clade with the 3 apomictic *Meloidogyne* that were constructed from the concatenated alignments covering a total of 2,202 protein-coding genes.

**Fig 5:**
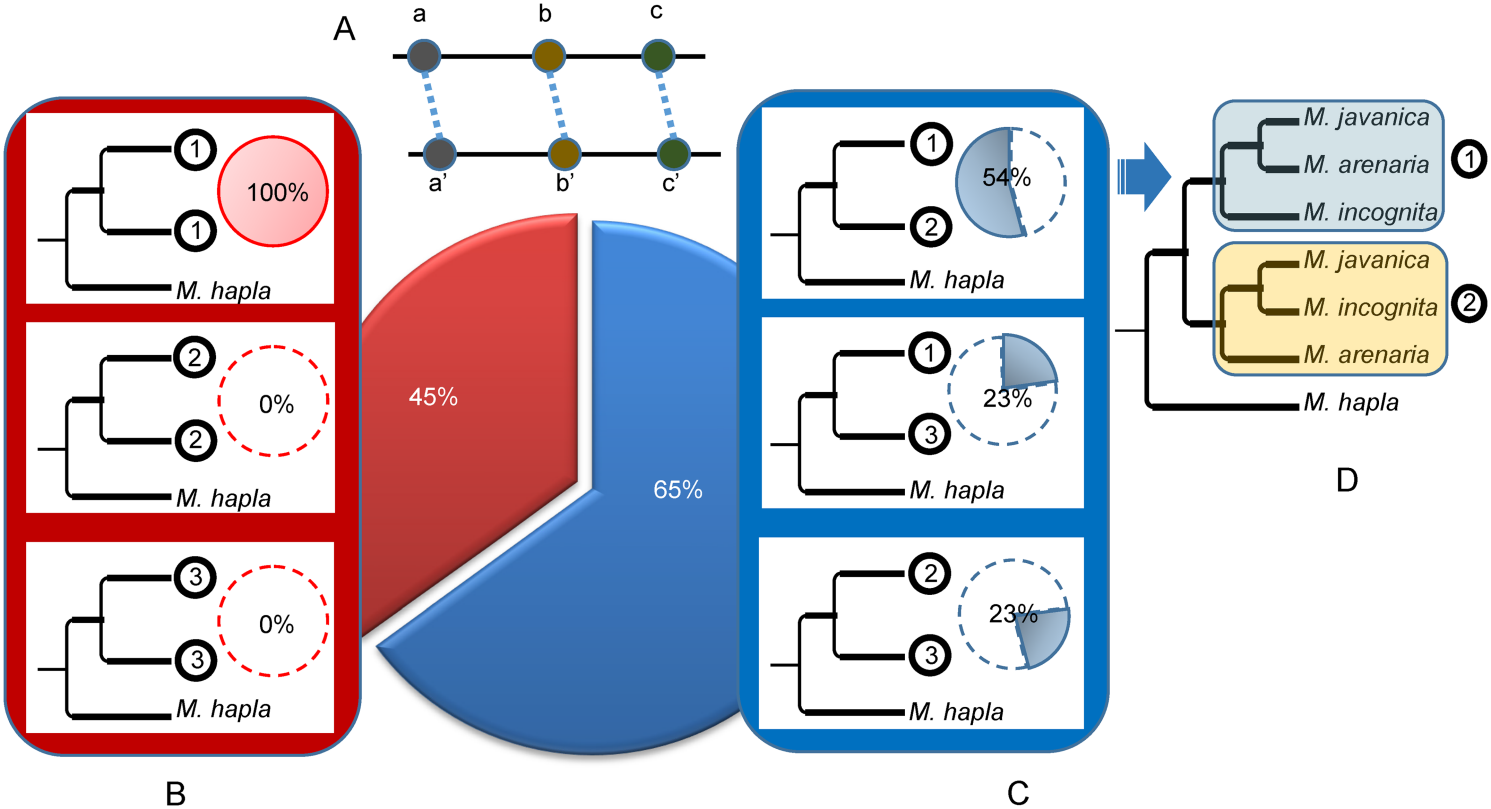
Combinations of topologies for duplicated regions in the three parthenogenetic *Meloidogyne*. (A) Schematic representation of two duplicated regions each containing 3 collinear genes (a, b, c and a’, b’, c’). (B) ML trees combining twice the same topology among (1), (2) and (3), for the two duplicated regions (further detailed in Fig 4). These trees suggest the two duplicated regions have the same evolutionary history. (C) ML trees combining two different topologies for the duplicated regions. These trees suggest the two duplicated regions have different evolutionary histories. The relative frequencies of trees combining twice the same (red) or two different (blue) topologies are indicated in the big central pie chart. Relative frequencies within the red and blue categories are indicated by small pie charts next to the corresponding schematic tree. (D) The most frequently observed ML trees consist in a combination of topologies (1) and (2).

Only three possible bifurcating monophyletic topologies exist to separate the three mitotic *Meloidogyne* (Fig 4): (1): (*Mi*, (*Ma*; *Mj*)), (2): (*Ma*, (*Mi*; *Mj)*) or (3): (*Mj*, (*Ma*; *Mi*)). We identified 60 such monophyletic clades and the most frequent corresponded to topology 1, observed 33 times. Topology 2 was observed 15 times and topology 3, 12 times (Fig 4). A total of 20 trees combined two of the three topologies mentioned above and allowed testing whether or not the two duplicated regions present the same evolutionary history across the 3 mitotic *Meloidogyne*. Among these 20 trees, a majority (13) combined two different topologies for the apomictic *Meloidogyne*, suggesting that the two regions have different evolutionary histories rather than a common ancestral duplication (Fig 5). The combination of topologies 1 and 2 was observed 7 times (see Fig 3B for an example). The combination of topologies 1 and 3 and the combination of topologies 2 and 3 were each observed 3 times. Only 7 of the 20 trees showed twice the same topology; and in all these cases this was twice topology 1.

**Fig 6:**
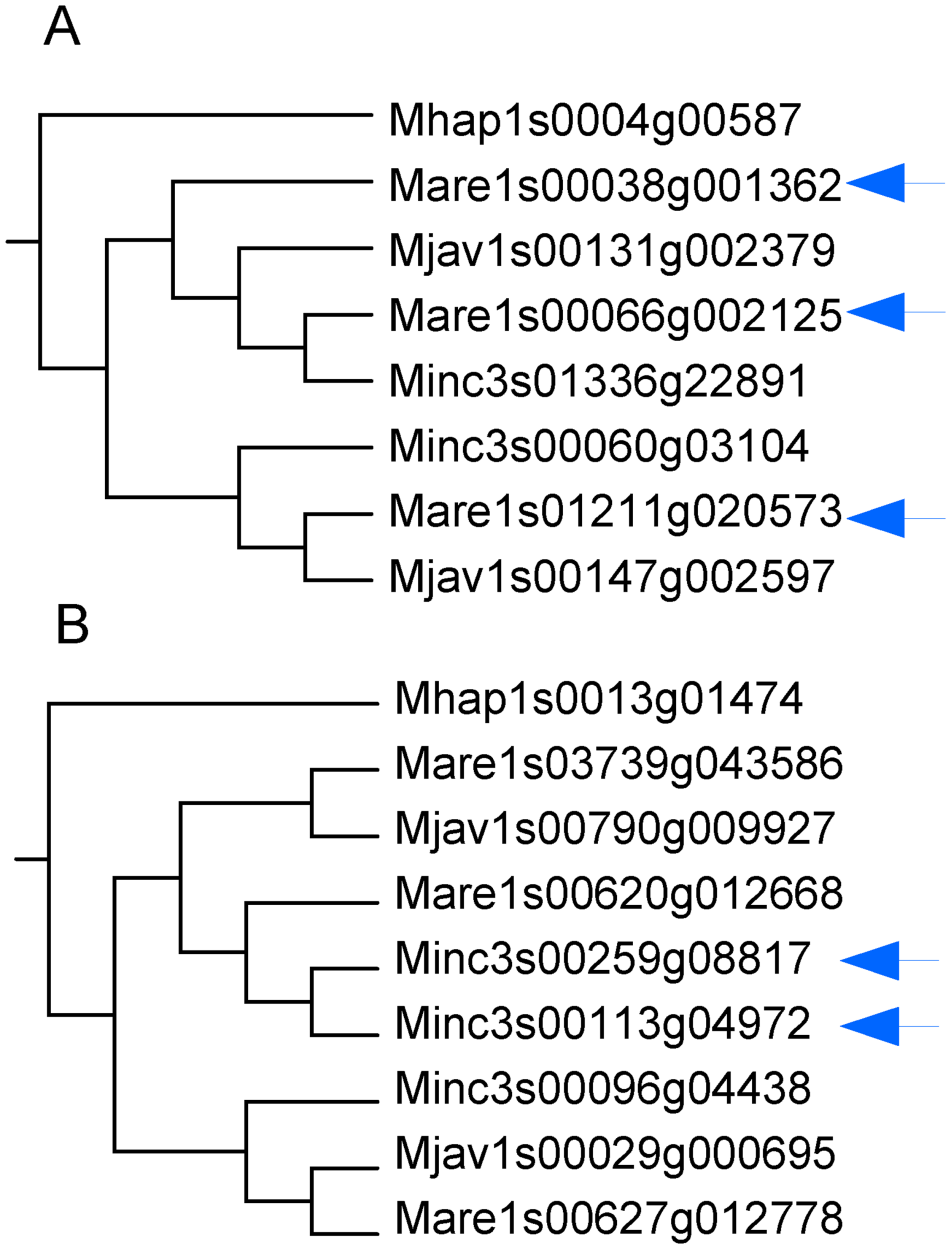
Allele-like versus homeologous relationship between genes in more than two copies. (A) An example of homoeologous relationship where each *M*. *arenaria* gene copy (arrows) clusters with the copy of another species. (B) An example of allele-like relationship where two of the three *M*. *incognita* gene copies (arrows) are more similar to one another than they are to a copy from another species.

Part of the genes forming duplicated regions were present in more than two copies in at least one apomictic species. We identified 387 groups of homologous collinear genes (total of 4,262 genes) forming 3 or 4 duplicated regions in at least one mitotic *Meloidogyne* and at least 2 duplicated regions in the other mitotic. To decipher the evolutionary history of these additional copies, we counted the number of times the third or fourth copies hold a recent in-paralog (or allele-like position) relative to another copy, *vs*. the number of times these copies were in a new independent branching position (Fig 6). For genes present in 3 copies in a given genome assembly, the number of allele-like relationship was significantly lower (binomial test, P<10^−6^) than the number of new phylogenetic position, for all three mitotic species (Table 5). Hence, genes present in 3 copies more frequently formed a new independent branch in the phylogenetic trees than species-specific recent paralogs or allele-like branches. For genes in four copies within a given genome assembly, the number of allelic-like relationship was lower than the number of new positions in all species but the difference was significant (binomial test, P<10^−5^), for *M*. *arenaria* only (Table 5).

**Table 5:** Number of trees supporting allelic-like *versus* homoeologs evolutionary relationship for genes in regions present in 3 or 4 copies per species.

Nb copies	Relation	*M*. *incognita*	*M*. *javanica*	*M*. *arenaria*
3	Allele-like	33 trees ***	11 trees ***	19 trees ***
	Homoeologs	95 trees	80 trees	226 trees
4	Allele-like	3 trees	5 trees *NS*	6 trees **
	Homoeologs	1 trees	12 trees	31 trees

***: *P*<10^−6^,

**: *P*<10^−3^, *NS* = non-significant, according to a binomial test

Overall, this ensemble of results suggests that the duplicated genome regions have different evolutionary histories and thus probably result from allopolyploidization. These pairs of regions and the corresponding gene pairs can thus be considered as homoeologous [32]. This term refers here to pairs of genes that originated by speciation and were brought back together in the same genome by hybridization.

### Asexual *Meloidogyne* share nearly identical mitochondrial genes

**Fig 7:**
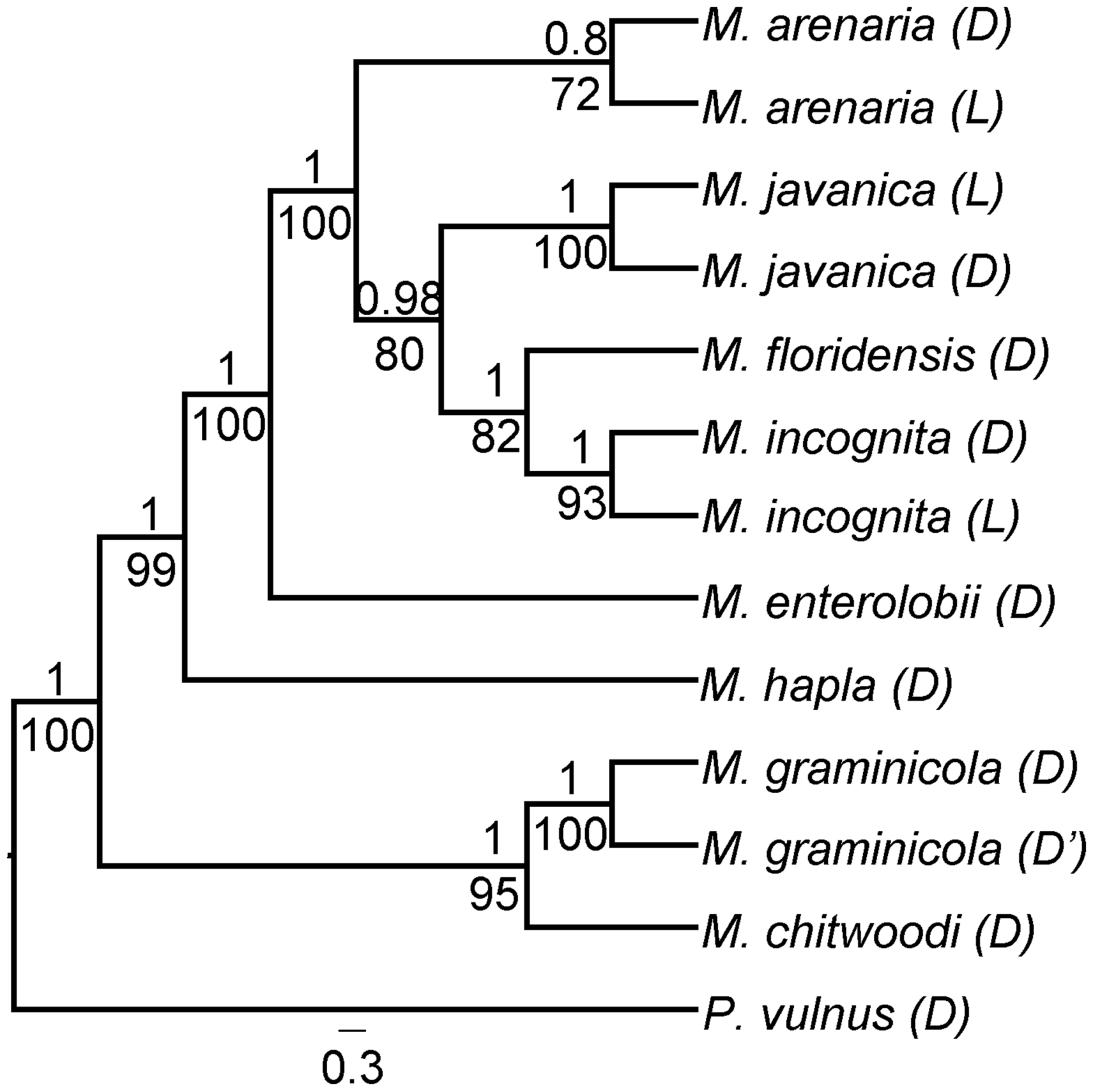
Mitochondrial phylogeny. Consensus phylogeny obtained using ML and Bayesian analyses on 14 concatenated mitochondrial genes (12 protein-coding and 2 rRNA). Posterior probability (above) and bootstrap (below) support values are given at each corresponding branches. Phylogenies were rooted with *Pratylenchus vulnus* as an outgroup. The tree is represented without taking branch lengths into an account for better visibility of phylogenetic relations; the same tree with actual branch lengths is available in S6 Fig. Species with a (L) suffix indicate sequences coming from the genome sequencing effort undertaken as part of this paper. Species with a (D) suffix indicate sequences coming from other databases. Full species names and accession numbers for the sequences coming from external databases are given in S1 Table.

To reveal the maternal evolutionary history of *Meloidogyne* species included in our analysis, we performed a phylogeny based on mitochondrial protein-coding genes as well as the 12S and 16S rRNAs (S1 Text). The phylogenetic tree (Fig 7, S6 Fig, S1 Table) returned the following highly-supported topology: (*Ma*,((*Mi*,*Mf*),*Mj*))). This topology corresponds to topology 2, the second most frequently observed in the analysis of the 60 groups of homoeologous duplicated regions (Fig 4). This suggests that genomic regions displaying topology 2 correspond to the maternal contribution to the nuclear genome. We also measured the average nucleotide divergence of mitochondrial genes between the 3 apomictic *Meloidogyne* (*Mi*, *Mj* and *Ma*). On average, the inter-species nucleotide divergence was very low (0.17%) and ranged from 0.00 to 0.33%. In contrast, the average nucleotide divergence between the meiotic *M*. *hapla* and the three mitotic was 24.50% and ranged from 24.42 to 24.58%. Hence, mitochondrial phylogenetic analysis reveals a high similarity between mitochondrial genomes of the three mitotic species and a substantial distance to their sexual relative *M*. *hapla*.

### The duplicated genomes of asexual *Meloidogyne* enable positive selection between gene copies

**Fig 8:**
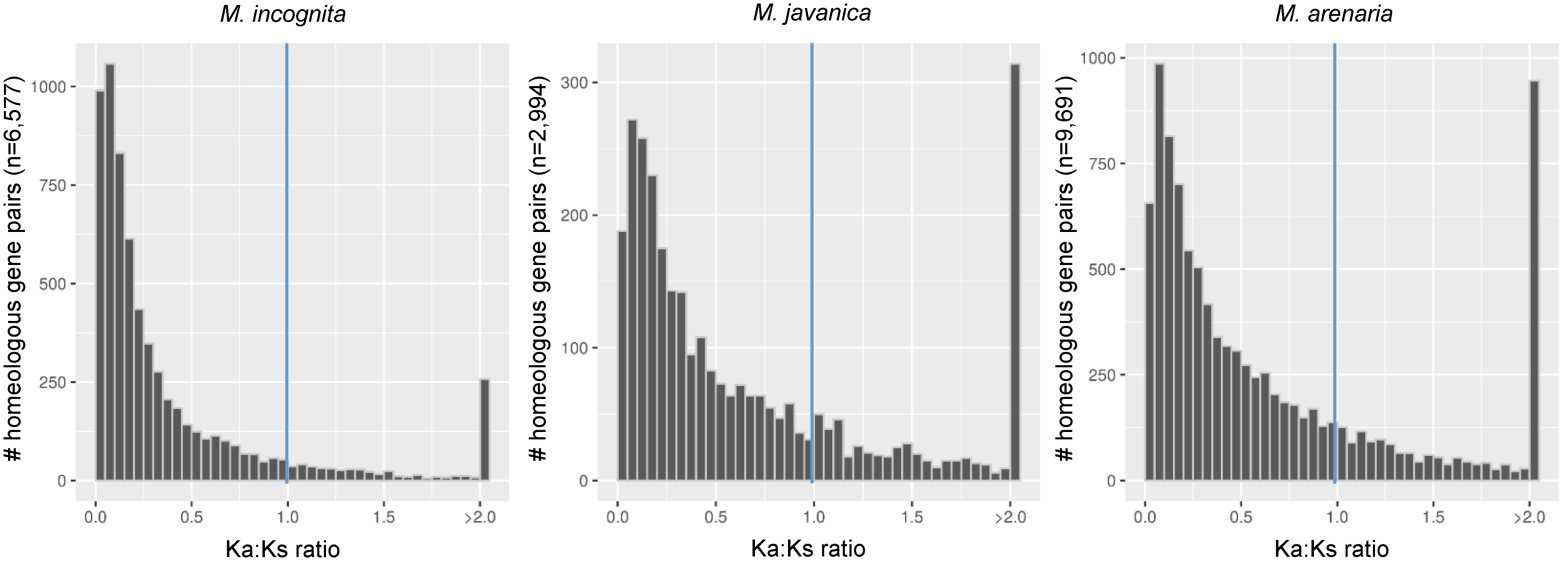
Distribution of the Ka / Ks ratio for pairs of collinear genes in asexual *Meloidogyne*. Histogram displaying the distribution of the ratio of rates of non-synonymous (Ka) / rates of synonymous (Ks) nucleotide substitutions for pairs of duplicated collinear genes in *M*. *incognita*, *M*. *javanica* and *M*. *arenaria*. Pairs with a Ka / Ks *>* 1 indicate positive selection between genes.

We tested whether gene redundancy due to the duplicated genomic regions might result in a relaxation of selective pressure on the gene copies. We employed two different strategies to detect positive and episodic diversifying selection. One raw approach based on pairwise computation of the ratios of rates of non-synonymous (Ka) vs. synonymous mutations (Ks); and a phylogeny-based statistical approach. We found that 612 (8.8%) (*Mi*), 698 (22.4%) (*Mj*) and 2,061 (20.9%) (*Ma*) homoeologous gene pairs had a Ka / Ks ratio greater than 1, indicating possible positive selection (Fig 8). In a second, phylogeny-based approach, we looked for signs of episodic diversifying selection (EDS) in homoeologous genes shared by the 3 apomictic *Meloidogyne* genomes. We retrieved all homoeologous genes present in the three apomictic species and *M*. *hapla* and in 2 or more copies in at least one apomictic *Meloidogyne*. We found 1,735 such groups and used them to generate multi-gene alignments and their respective ML midpoint-rooted phylogenies. Using the random effects branch-sites model [33], we found 172 (*Mi*), 109 (*Mj*) and 208 (*Ma*) gene copies showing evidence of EDS (S7 Fig for an example) at the 0.05 confidence level (P_Holm: corrected for 9 tests using the Holm-Bonferroni procedure). Among these genes, 20 (*Mi*), 21 (*Mj*) and 47 (*Ma*) were also found to have Ka / Ks ratios >1.

To assess which functional categories were affected by positive selection or EDS, we examined Pfam domains and gene ontology (GO) terms associated to these genes (S1 Text). Overall, a large variety of Pfam domains and associated GO terms were identified among proteins encoded by genes under positive selection (Ka / Ks >1) or subject to EDS, in the three apomictic species. As many as 363, 304 and 674 distinct Pfam domains, corresponding to 177, 167, and 310 distinct GO terms were found in proteins encoded by genes with Ka / Ks >1 in *Mi*, *Mj* and *Ma*, respectively (S2 Table). Similarly, we identified 123, 78 and 174 distinct Pfam domains, corresponding to 93, 56 and 112 distinct GO terms, in proteins encoded by genes under EDS in *Mi*, *Mj* and *Ma*, respectively (S3 Table). Regardless of the dataset (Ka / Ks or EDS), few Pfam domains and GO terms were common to the 3 apomictic species and the majority of them were related to enzymatic, binding and metabolic activities as well as cell cycle-related and transport functions (S2 and S3 Tables). Mapping raw GO terms to the more generic GO-slim terms, revealed more overlap between the three mitotic species. The vast majority of GO-slim terms were shared by the three species in the EDS as well as in the Ka / Ks datasets (S2 and S3 Tables). Overlap between the EDS and Ka / Ks datasets was also high as 23 of the 28 GO terms shared by the three mitotic in the EDS dataset were also shared by them in the Ka / Ks dataset. These terms were mainly related to diverse enzymatic, catabolic, metabolic and biosynthetic functions. We identified significantly enriched GO and GO-slim terms in the Ka / Ks datasets as compared to the rest of homoeologous gene pairs (S2 Table). However, no significantly enriched GO or GO-slim term was common to the three species. No GO or GO-slim term was found to be enriched at the significance threshold (FDR<0.05) in the proteins under EDS as compared to the rest of homoeologous proteins.

### Homoeologous gene copies show functional divergence at the expression pattern level

**Fig 9:**
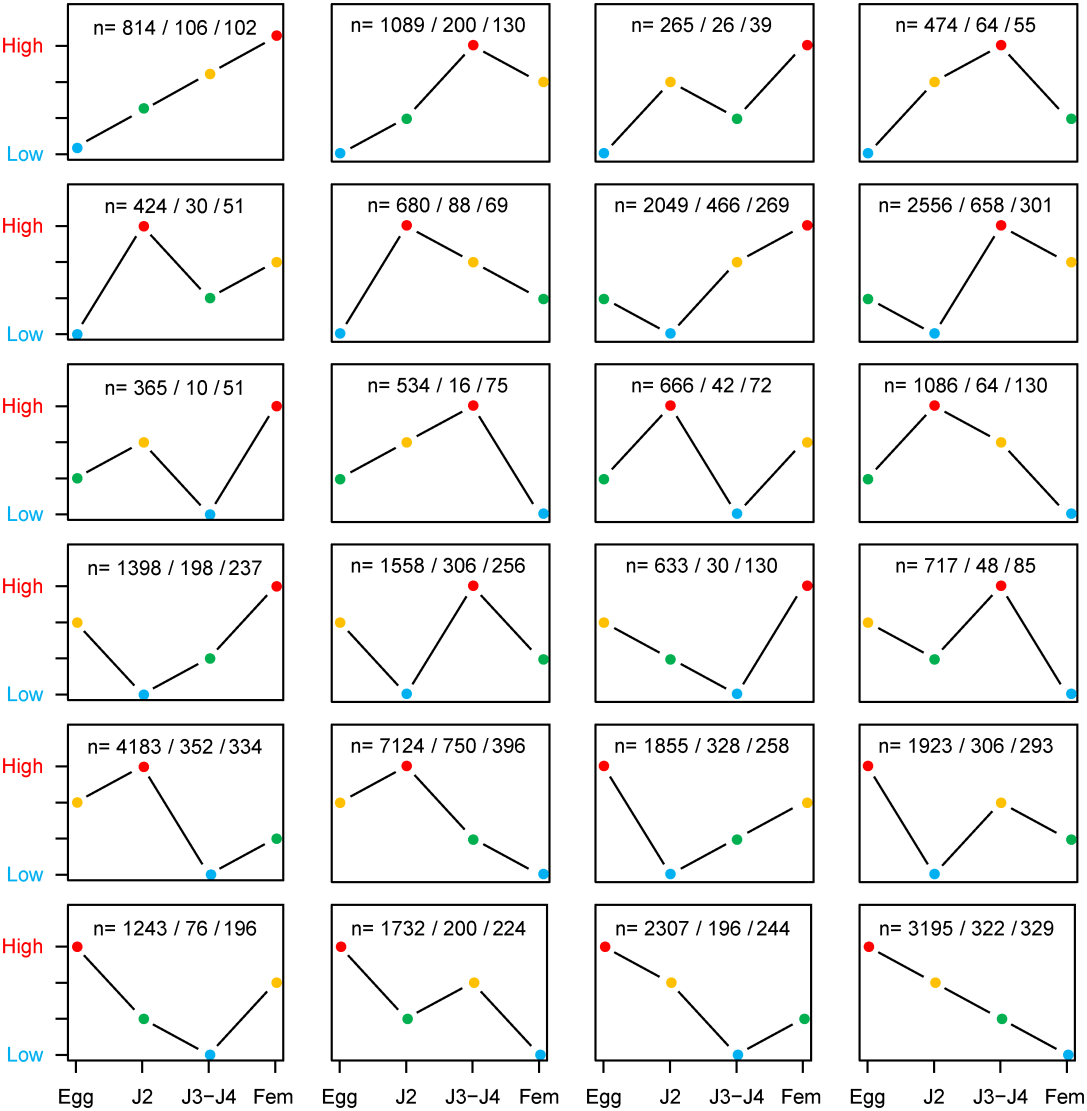
Distribution of *M*. *incognita* genes and copies in the expression clusters. For each of the 24 expression clusters, the information ‘n = a/b/c’ indicates. a: the total number of *M*. *incognita* genes having this expression pattern. b: the number of times the two genes from a same pair have this same expression pattern. c: the number of genes part of a pair having this pattern, while the other cognate gene has a different expression pattern (i.e. belongs to another expression cluster). Hence, c refers to gene pairs with diverged expression patterns. The expression ranks across the four developmental life stages (eggs, J2 infective juveniles, J3-J4 larval stages and adult females) are represented in colors as follows rank1: lowest expression (blue), rank2: second lowest expression (green), rank3: second highest expression (orange) and rank4: highest expression (red).

Functional divergence between gene copies can be viewed at different levels, including the biochemical function, the biological process or the expression pattern. Gene copies featuring the same biochemical function but expressed in different tissues or time points can be involved in different biological processes (e.g. development of different organs). To biologically assess whether functional divergence actually occurs between homoeologous gene copies, we analyzed their expression patterns across four developmental life stages (eggs, J2 infective juveniles, J3-J4 larval stages and adult females) of *M*. *incognita* using RNAseq (methods). We generated between 60.5 (J3-J4 replicate 2) and 94.2 million (egg replicate 2) 2x75bp paired-end reads across the 12 libraries (4 stages x 3 replicates). After all the cleaning steps, between 11.8 (J3-J4 replicate 2) and 48.5 (egg replicate 2) million clean paired-end reads were mapped to the *M*. *incognita* reference genome. The proportion of paired-end reads aligned on the genome varied between 75.0% (female replicate 3) and 96.2% (egg replicate 1). The majority of the read pairs (57.2–76.1%) mapped to a unique position on the genome (Table 6). Overall, a total of 42,705 *M*. *incognita* protein-coding genes (or 97.7% of 43,718 in total) had a log10(FPKM+1) >1 in at least one sample and were considered as expressed. After filtering out low-signal values as well as low-complexity genes, a total of 38,870 expressed genes remained, including 6,767 homoeologous gene pairs (7,299 initially). This ensemble of expressed genes was classified into 24 distinct expression clusters (methods). We assessed whether homoeologous gene pairs tended to fall in the same expression cluster or in different expression clusters, indicating functional divergence. We found that 4,326 out of 6,767 (63.9%) expressed homoeologous gene pairs showed signs of diverged expression by being assigned to two different expression clusters (Fig 9). Interestingly, pairs of homoeologous genes showing evidence of positive selection in *M*. *incognita* tend to be more often in different expression clusters than those showing no sign of positive selection (74.1% vs 63.9%, p-value <10^−7^). This ensemble of results biologically confirms that the peculiar allopolyploid genome structure of asexual root-knot nematodes is associated to functional divergence between gene copies.

**Table 6:** RNAseq of four *M*. *incognita* life stages, sequencing, cleaning and mapping.

Sequencing		Cleaning	Mapping to the genome			
Libraries	Initial read pairs	Cleaned read pairs	Mapped read pairs	% mapped	% unique	% multi
egg-1	87 074 210	48 295 627	46 074 870	95.40	76.08	19.32
egg-2	94 158 382	48 492 099	46 562 165	96.02	76.02	20
egg-3	66 074 037	32 259 963	30 478 818	94.48	75.17	19.3
J2-1	72 958 025	32 264 784	29 720 974	92.12	72.42	19.7
J2-2	78 380 200	36 426 756	33 827 380	92.86	72.17	20.69
J2-3	80 099 032	32 972 463	28 181 323	85.47	67.05	18.42
J3J4-1	92 349 648	18 410 414	16 356 975	88.85	69.75	19.1
J3J4-2	60 483 114	11 793 967	10 875 445	92.21	73.35	18.86
J3J4-3	84 491 183	14 725 039	13 804 525	93.75	72.52	21.23
adult-1	68 718 904	15 362 025	14 462 874	94.15	72.08	22.07
adult -2	76 861 805	16 904 124	13 226 056	78.24	59.74	18.5
adult -3	79 680 298	20 839 034	15 621 417	74.96	57.15	17.81

### Asexual *Meloidogyne* genomes are rich in genes and in transposable elements

Although recombination can prevent the accumulation of TE, sexual reproduction can favor transmission of TE between individuals. In parallel, hybridization can initially favor TE multiplication by exposing naïve host genomes to TE uncontrolled by their inactivation machinery (e.g. chromatin modification or small RNAs). Thus, we investigated whether differences in TE abundance could be revealed between sexual and asexual *Meloidogyne*. With 29.2% of its genome occupied by TE, *M*. *hapla* has a relatively high TE abundance compared to other nematodes. Indeed, TE span 16.5% and 22.4% of the genomes of *C*. *elegans* and *C*. *briggsae*, respectively [34], 18% in *Trichinella spiralis* [35], 14–15% in *Brugia malayi* [36] and 22% in *Bursaphelenchus xylophilus* [37]. We found that TE span 50.0, 50.8 and 50.8% of the genome assemblies of the asexual *Mi*, *Mj* and *Ma*, respectively (Table 2). The genomes of the asexually reproducing *Meloidogyne* thus appear to be particularly rich in TE and 1.7 times richer than the only sexual *Meloidogyne* genome available to date. Consistent with this observation, Class I retro-elements are on average 1.5 times more abundant in the asexual species. Within Class I elements, DIRS-like (*Dictyostelium* intermediate repeat sequence) appear to have undergone a particular expansion in the asexuals as they are on average 5.5 times more abundant than in the sexual species. Class II DNA transposons are 1.9 times more abundant in the three apomictic species than in the *M*. *hapla* genome. Although Helitron occupy a comparable proportion in asexuals and in the sexual, all the other categories are more than 2 times more abundant in the asexuals. This includes Maverick-like and TIR (terminal inverted repeats) elements as well as “unclassified” TE that possessed characteristics of Class II elements but could not be further assigned to one family. The rest of the potential TE is in the “other” category, which gathers DNA fragments displaying contradictory features of both Class I and II elements. This category was also more abundant in the asexuals than in the sexual species (~1.8 times). This overall abundance of TE in asexual *Meloidogyne* has implications at the protein-coding level. While 27–30% of the protein-coding genes of asexual *Meloidogyne* are totally included within TE, only 17% of *M*. *hapla* genes are within TE. Hence, TE abundance partly explains the higher number of genes observed in the asexual *Meloidogyne* (43,718–102,269 compared to 14,207 in *M*. *hapla*).

**Fig 10:**
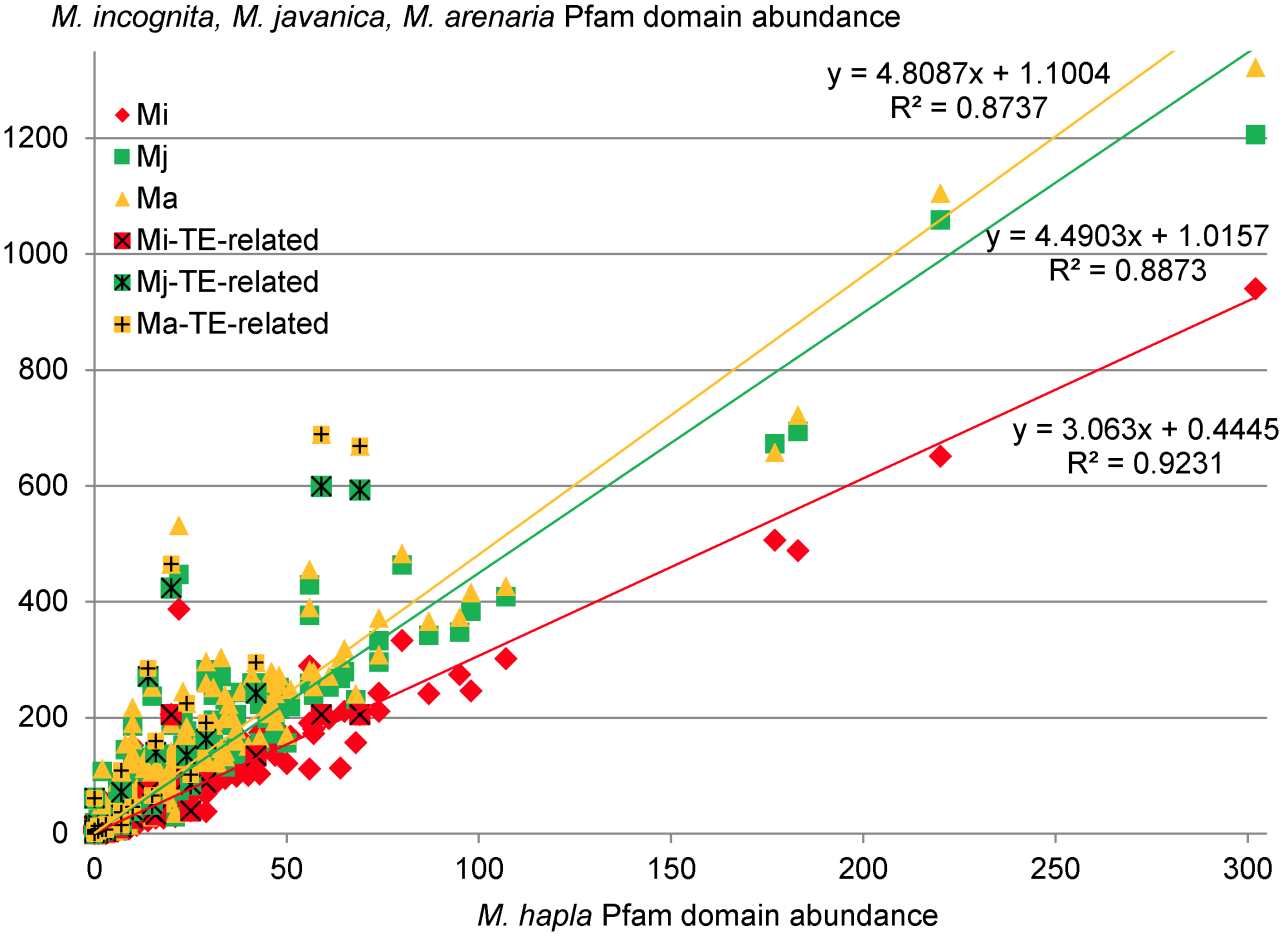
Pfam domain abundance in asexual *Meloidogyne* as a function of the abundance in *M*. *hapla*. The x-axis represents abundance of Pfam domains in *M*. *hapla* and the y-axis represents abundance of the same domains in *M*. *incognita* (red diamonds) *M*. *javanica* (green squares) and *M*. *arenaria* (yellow triangles). Linear regressions of *M*. *incognita*, *M*. *javanica* and *M*. *arenaria* Pfam domain abundances as a function of the abundance in *M*. *hapla* are plotted alongside their respective equations and correlation coefficients. Pfam domains associated to transposable elements (TE) are represented as crossed red, green and yellow squares in *M*. *incognita*, *M*. *javanica* and *M*. *arenaria*, respectively.

We tested whether the higher gene numbers observed in asexual *Meloidogyne* were homogeneously distributed along all protein domain families. We plotted the abundance of protein domains in *Mi*, *Mj* and *Ma* as a function of their abundance in *Mh* (Fig 10). The abundances of protein domains in *Mi*, *Mj* and *Ma* were all positively correlated to the abundance in *Mh* (R^2^ = 0.92, R^2^ = 0.89 and R^2^ = 0.87 for *Mi*, *Mj* and *Ma*, respectively). The slopes of the linear regressions were 3.06, 4.49 and 4.80 for *Mi*, *Mj* and *Ma*, respectively, suggesting that most of the protein domains are between 3 and 5 times more abundant in the three asexuals as compared to *M*. *hapla*. We compared the abundance of Pfam domains known to be found in TE-related genes and important for their own transposition activity (e.g. reverse transcriptase, integrase, transposase) in the four *Meloidogyne* species. We found that, on average, these domains were 3.4 to 9.8 times more abundant in asexual *Meloidogyne* than in *M*. *hapla* (S4 Table). For instance, rve (integrase core domain) is present in 205–689 copies in the three asexuals while it is found in only 59 copies in *M*. *hapla*. Similarly, the DDE_ 3 (DDE superfamily endonuclease) domain is absent in the *M*. *hapla* protein set while it is found in 13–61 copies in the three asexuals. This suggests that expansion of at least some families of TE might be in part responsible for the higher number of protein-coding genes in the asexuals.

## Discussion

### Asexual root-knot nematodes have large and diverged polyploid genomes

Meiotic pairing and segregation require high sequence identity and collinearity between homologous chromosomes. Usually, sequencing the genome of a diploid sexual animal involves performing repeated cycles of inbreeding to obtain lineages virtually homozygous at all loci. Genome assembly then results in collapsing all these virtually identical paternal and maternal variants into one single haploid reference sequence. We actually observed this in *M*. *hapla* which was assembled into a ~54 Mb genome [24], similar to previously reported measures of haploid genome sizes (~50 Mb) [38,39], and confirmed by our flow cytometry measures (~60 ± 1.5 Mb, Table 1). Concordance of genome assembly size with experimental measures, associated to the absence of extensive duplications of genomic regions, indicate a canonical sexual diploid genome. The haploid chromosome number of *M*. *hapla* is n = 16, similar to the putative ancestral haploid number of chromosomes (n = 18) in *Meloidogyne* species [19,22,40]. Hence, we can hypothesize that the ancestral haploid genome size for a *Meloidogyne* is ~55–60 Mb with n = 16–18 chromosomes. The genome assembly sizes of the three mitotic *Meloidogyne* species we describe here reach ~180 Mb, ~235 Mb and ~260 Mb for *Mi*, *Mj* and *Ma*, respectively. This represents ~3x, ~4x and >4x the expected haploid genome size for a *Meloidogyne* species. Flow cytometry measures of nuclear DNA content suggest an even larger genome size of up to ~300 Mb for *Mj* and *Ma* (Table 1). Hence, the genomes of apomictic *Meloidogyne* (~180–300 Mb) are 3 (*Mi*) to 5 times (*Ma*) bigger than the ancestral haploid genome size for a *Meloidogyne* species. Furthermore, Pfam domains are on average 3, 4.5 and ~5 times more abundant in the mitotic *Mi*, *Mj* and *Ma* genomes, respectively than in the *M*. *hapla* genome (Fig 10). Moreover, alignment of the CDS to the respective *Meloidogyne* genomes showed that while in the sexual *M*. *hapla*, most CDS map one single locus; we observe a peak at 3 matching loci for *Mi*, a peak between 3 and 4 for *Mj* and a peak at 4 matching loci for *Ma* (Fig 1). Finally, we showed that a substantial proportion of gene copies form collinear blocks of duplicated genome regions. Taken together, these results strongly suggest that the genomes of mitotic *Meloidogyne* are polyploid, with *M*. *incognita* being most likely triploid, *M*. *javanica* tetraploid and *M*. *arenaria* tetra- to pentaploid. Observation of chromosome numbers (supplementary discussion S1) further supports polyploidy of the three asexual *Meloidogyne*. Similarity between genome assembly sizes and measures of total nuclear DNA content *via* flow cytometry suggests that most of the former paternal and maternal donor genomes have been separately assembled, probably due to their high within-species divergence. Indeed, the duplicated collinear genome regions span several Mb and thousands of genes in each mitotic *Meloidogyne* genome and show a similarly high within-species average nucleotide divergence of ~8%, confirming initial analysis of the *Mi* draft genome[23]. Likewise, the per-site synonymous substitution rate (Ks) of collinear gene pairs that define these duplicated genome regions had a very similar median of 0.1 for all three species. This homogeneity of nucleotide divergence and Ks between pairs of collinear regions for the three mitotic *Meloidogyne* species suggests that they have duplicated in a same time window and thus separated for a similar amount of time.

### The genome structures are consistent with absence of meiosis

Due to multiple synteny breakpoints, no long scaffold could be aligned on its whole length to another long scaffold in any of the three mitotic *Meloidogyne*. This rearranged chromosomal structure combined with the high average divergence between homoeologous blocks suggest chromosome pairing must be complicated if not impossible. Furthermore, in *Mi* and *Ma*, we observed collinear regions present in palindromic or tandem arrangement on a same scaffold. Such structures, similar to the ones observed in the ancient asexual bdelloid rotifer *A*. *vaga* [5], appear incompatible with segregation of homologous chromosomes in conventional meiosis. Both the difficulty of pairing homologous chromosomes and the impossibility to separate the genome in two equivalent chromosome sets are consistent with the absence of meiosis and the strict asexual reproduction of these organisms. Palindromes or tandem blocks were not observed in the genome of the meiotic *M*. *hapla*, and because this genome presents high contiguity (the highest for a *Meloidogyne*); this most probably represents true absence. Similar analysis could not be performed for the *M*. *floridensis* genome; due to its low contiguity and fragmented nature, (only 12 genes were found in one pair of duplicated regions).

### The asexual *Meloidogyne* species most likely have recent hybrid origins

The duplicated genome regions in mitotic *Meloidogyne* tend to be more similar across different species than they are to their other copies within the same species. Furthermore, when duplicated regions form two or more clades in phylogenomic analysis, these clades more frequently present distinct topologies. Thus, collinear duplicated regions within a species have different origins and evolutionary histories and probably do not originate from common ancestral allelic regions that accumulated mutations separately (*i*.*e*. no Meselson-White effect). Contrasting with the high within-species divergence of duplicated blocks in the nuclear genome (avg. divergence ~8%), mitochondrial genes are almost identical in *Mi*, *Ma* and *Mj* (avg. divergence ~0.17%). This confirms previous observations that these three species share virtually identical mtDNA markers [26,41,42] and suggests that *Mi*, *Mj*, and *Ma* share closely related or common maternal ancestors. The mitochondrial genome is expected to accumulate mutations faster than the nuclear genome (e.g. 100–1,000 times more rapidly than the nuclear genome in *C*. *elegans* [43–45]). Hence, the divergence time between the nuclear genome copies within a same species is assumed much higher than the divergence time between the different species themselves, based on mitochondrial data. Inter-specific hybridization is the most likely hypothesis that could resolve at the same time the discrepancy between low between-species mitochondrial divergence and high within-species nuclear divergence levels and the observed topologies in the phylogenomic analysis (alternative hypotheses are discussed in S1 Text). We propose that not only *Mi* but also *Mj* and *Ma* most likely originated from multiple hybridization events with a same or closely related maternal donor lineage and different paternal donors. This confirms, at a whole genome scale, a previous formulation of this hypothesis based exactly on the same kind of observed discrepancy between low divergence in mitochondrial markers between species and high divergence in ITS nuclear markers within species of apomictic *Meloidogyne* [26]. In this regard, the case of salamander in the *Ambystoma* genus constitute an interesting parallel. Indeed, some unisexual species in this genus are characterized by their absence of meiosis, their various ploidy levels (from 2n to 5n), their hybrid origin and their closely related mitochondrial genomes [46].

In apomictic *Meloidogyne*, because the mitochondrial divergence is very low, the speciation between *Mi*, *Mj* and *Ma* as well as the hybridization and associated loss of sex must be very recent. The very low proportion of collinear genes lost in duplicated genomic blocks further support recent whole genome duplication events (*via* hybridization). Indeed, after a whole genome duplication event, regardless whether it involves hybridization or not, most of the redundant gene copies are expected to be lost relatively rapidly. For instance, it has been shown in teleost fish that 70–80% of the genes have been rapidly lost after the latest WGD event [47].

### Multiple hybridization steps probably led to the 3-5n polyploid genomes

Based on genome sizes, Pfam domain abundance and peaks of genes in 3, 4 or more copies, we estimated that *Mi* was most likely triploid while *Mj* and *Ma* were respectively most likely tetra to penta-ploids (see above). Some collinear regions are conserved in more than 2 copies within all the genomes, which allowed assessing the evolutionary histories of the third and fourth genome copies. For the three asexual *Meloidogyne*, the third copies of collinear genes were significantly more frequently similar to a cognate gene in another *Meloidogyne* species than to any of the other two copies found in the same species (Table 5). Thus, the third copies probably derive from a distinct hybridization event. This suggests a two-step hybridization process. First, homoploid hybridization (hybridization between two diploid AA and BB progenitors without associated genome doubling [48,49]) took place and led to a diploid AB hybrid. Then, a second hybridization between an unreduced AB gamete of the homoploid hybrid with a reduced C gamete of another sexual species led to the presence of three distinct copies (ABC) of nuclear genomes within a same species. It should be noted that unreduced gametes are frequently produced by inter-specific hybrids [50]. Although this hypothesis could explain the triploid genome of *M*. *incognita*, additional steps are needed to explain the tetra to penta-ploid genomes of *M*. *javanica* and *M*. *arenaria*. We hypothesize that the triploid bridge pathway described in several polyploid plants [51,52] could explain the transition between triploids (similar to *M*. *incognita*) and tetraploids (similar to *M*. *javanica*). Indeed, under this hypothesis, triploids can constitute a bridge towards tetraploidy by producing unreduced triploid gametes, that, by fusing with a haploid gamete, would lead to a tetraploid progeny. Those tetraploids would in turn produce diploid gametes that would combine with haploid gametes of other species, creating a new triploid. And this triploid, could in turn serve as an intermediate towards new tetraploid by fusing unreduced triploid gametes with haploid gametes, constituting a “triploid-tetraploid-triploid” circle as suggested in [53]. Finally, fusion of an unreduced triploid gamete with either a reduced gamete from a tetraploid or an unreduced gamete from a diploid, could lead to a pentaploid hybrid similar to *M*. *arenaria*.

In this perspective, the plant genus *Boechera* constitutes a good exemplary system for cases of hybridization at different ploidy levels and ecological success [54]. Indeed, it constitutes a genus in which both homoploid diploid and triploid hybrids are present with also less frequent tetraploids or species of higher ploidy levels. Similarly to the *Meloidogyne*, the hybrids are apomicts and highly heterozygous.

The whiptail lizards constitute an interesting similar example of animal with fully asexual reproduction. Like the asexual *Meloidogyne*, these lizards are of hybrid origin. Interestingly, several polylploid lineages were identified and they also present a fixed heterozygosity [55,56].

### High abundance of TE in asexual *Meloidogyne* possibly provides genome plasticity

Following loss of sexuality, it has been hypothesized that TE could invade the genomes because recombination would tend to favor their elimination [57]. Alternatively, it has been suggested that the only asexual animals that survive are those that control TE multiplication in their genomes. Examples supporting these two different hypotheses exist in the literature. In *Daphnia* arthropods, sexual reproduction seems to be correlated with an initial slower accumulation of TE in genomes whereas, at the long-term sex is associated with higher TE loads [15]. In parasitoid wasps, it has been shown that TE are more abundant in Wolbachia-induced asexual lineages than in sexual lineages [58]. However, whether this is a consequence of sex loss or of Wolbachia infection remains to be clarified. In contrast, in the ancient asexual bdelloid rotifer *A*. *vaga*, TE occupy only 3% of the genome and while a high diversity of TE was found, they are generally present at very low copy numbers [5]. This suggests that TE proliferation might be under control in this species. Recently, a comparison of the TE load in five sexual vs. asexual lineages of arthropods showed no evidence for TE accumulation in the asexuals [59]. In *Meloidogyne*, we found that TE occupy ~50% of the genomes of the three mitotic species while they occupy only 29% of the genome of *M*. *hapla*. Although it appears that TE have proliferated in the genomes of the asexual *Meloidogyne*, this might be a consequence of their hybrid origin rather than of their mode of reproduction. Regardless their origin, this abundance of TE might constitute a potential for genomic plasticity in the absence of sexual recombination. Supporting this hypothesis, some canonical full length TE were previously experimentally identified in these *Meloidogyne* species [60]. Furthermore, a Tm1 transposon has been identified in apomictic *Meloidogyne* but no homolog with an intact transposase could be found in the sexually-reproducing relative *M*. *hapla* [61]. Interestingly, the Cg-1 gene, whose deletion is associated to resistance-breaking strains of *M*. *javanica*, has been identified within one of these Tm1 transposons. Thus, TE possibly have an adaptive impact on these nematodes, including on their host plant range.

### The allopolyploid genomes are associated with positive selection between gene copies

*Mi*, *Mj* and *Ma* are exceptionally successful, globally distributed parasites of diverse agricultural crops [62,63]. Intriguingly, their geographical distributions and host ranges are wider than those of their sexual relatives. Furthermore, in controlled condition, they are able to overcome plant resistance within a few generations [22]. In the absence of sex and meiotic recombination to provide genomic plasticity and adaptability, their allopolyploid nature may provide benefits contributing to their parasitic success. First, polyploidy can provide the raw material for neo- and sub-functionalization of duplicated gene copies, resulting in novel genetic variation [64,65]. It has been shown in yeast that ploidy level is correlated to faster adaptation [66]. Also, it has been suggested that polyploidy could mask deleterious recessive alleles [67] and limit their accumulation *via* gene conversion between homologous regions [5]. Furthermore, allopolyploidy, by combining several genomes in one species, may lead to transgressive phenotypes that surpass those of the parent species *via* novel genetic combination and heterosis [50,67,68]. *Ambystoma* salamanders constitute one clear case of transgressive phenotype in animals. Indeed the hybrids between one native and one introduced species are ecologically fitter and more successful than the parental native species as well as other related species in the native environment [69,70].

Here, we tested whether the presence of several divergent genomic copies in a same species, could have functional consequences at the coding level. Hybridization brings together homoeologs chromosomes and therefore orthologous gene copies within an individual. Because the three mitotic *Meloidogyne* have very close mitochondrial genomes, their speciation was certainly recent. Hence, we can hypothesize that most of the high within-species nucleotide divergence between duplicated genomic regions is due to hybridization rather than long-term divergence. Most likely, the hybrid inherits orthologs that had retained similar function and following functional redundancy, selective pressure on these genes may relax and drive them to different evolutionary trajectories [71,72]. In some cases, the relaxation of selective pressure can allow emergence of new adaptive mutations. We have shown that ~8 to 20% of gene copies coming from the duplicated genomic regions harbor signs of positive selection. A diversity of Pfam domains and associated gene ontology terms were predicted in proteins encoded by positively selected genes. Although many terms and domains were related to enzymatic and other catalytic functions, there was a poor overlap between the three apomictic species, and different domains and functions were specifically enriched in positively selected genes in each species. These observations suggest that the functional consequences of the hybrid genome structure were different in each species.

### Functional divergence at the expression pattern level

In the model root-knot nematode *M*. *incognita*, we showed that more than 60% of homoeologous gene copies display diverged expression patterns. These gene copies resulting from hybridization have only single-copy equivalents in the sexual relative *M*. *hapla*. This biological confirmation of functional divergence suggests that additional genes in asexual root-knot nematodes are not just merely functionally redundant with their single-copy orthologs in the sexual relatives but actually support plasticity and variability. Thus, we can assume that the allopolyploid genome structures of asexual root-knot nematodes provide them with a reservoir of variability and adaptability that could partly compensate the absence of sexual reproduction. Noteworthy, these results are consistent with two other recent studies of hybrid animal genomes (the Atlantic salmon and the frog *Xenopus laevis*) that both also showed extensive functional divergence at the expression level between homoeologous gene copies [73,74]. Interestingly, we noted that the proportion of expression divergence is significantly higher (>70% vs. >60%, p-value<1.10^−7^) in homoeologous gene pairs that are under positive selection. These gene pairs combine both divergence at the expression level and accumulation of non-synonymous mutations that could lead to functional divergence at the biochemical level. They are thus the most obvious candidates for neo or sub functionalization.

### Concluding remarks

How an animal can survive without sexual reproduction and compete with its sexual relatives remains an evolutionary puzzle. Intriguingly, asexually reproducing (apomictic) root-knot nematodes outcompete their sexual relatives as plant parasites of global economic impact. We have shown here that the genomes of the apomictic *Meloidogyne* are duplicated most likely because of a complex series of hybridization events. Although the parental lineages are unknown, they probably belong to the sexual relative clades. Hence, this parasitic success could be viewed as a case of transgressive phenotype, where the ecological success of the hybrid progeny surpasses those of the parents [27,68]. Furthermore, hybridization has been proposed as an important evolutionary phenomenon that could give rise to new parasites and pathogens. For instance, hybridization of two host-specific plant parasitic tephritid fruit flies gave rise to a new species able to parasitize a new invasive host plant [75]. Similarly, hybridization of two *Blumeria* fungal pathogens gave rise to a new species that is able to infest a host plant of economic interest resistant to both progenitor species [76]. In the asexual root-knot nematodes, the presence of duplicated and diverged genomic regions probably promotes functional novelty between resulting gene copies, following positive selection. We confirmed this functional divergence at the expression level at the whole genome scale. Furthermore, the TE-rich nature of their genomes might also foster genomic plasticity not only actively by TE movements across the genomes but also passively by promoting chromosomal shuffling between these repeated genomic regions. Such a TE-promoted chromosomal shuffling associated to adaptation to different host plants has already been shown in a plant-pathogenic fungus [77]. Part of the intriguing success of mitotic asexual *Meloidogyne* could thus reside in their duplicated, diverged and TE-rich genomes resulting from hybridization. It would be interesting to explore the potential for plasticity and adaptation in the genomes of other asexual animals, particularly parasites and pathogens, to assess whether convergent or independent genomic characteristics support this potential.

## Materials and methods

### Genome assembly

DNA samples preparation protocols for genome sequencing are detailed in the S1 Text. Genome assemblies were performed in four steps, following the same procedure as developed for resolving the degenerate tetraploid genome structure of the bdelloid rotifer *A*. *vaga* [5]: (i) assembly of 454 data into contigs, (ii) correction of the 454 contigs using Illumina data, (iii) scaffolding of 454 contigs and (iv) gap closing using Illumina data. For the first step, we used the multi-pass assembler MIRA [78] version 3.9.4 (normal mode, default options except the number of cycles) to generate contigs from the 454 genomic libraries (S5 and S6 Tables). Although computationally demanding, running MIRA with multiple cycles is particularly appropriate to separate heterozygous regions in genomes, as anticipated in polyploid species. Moreover, Sanger reads of the *M*. *incognita* first draft genome sequence [23] were used to generate the current assembly. Twelve (*M*. *arenaria* and *M*. *javanica*) or sixteen (*M*. *incognita*) cycles were performed to separate a maximum of repeats and heterozygous regions. We subsequently used Illumina data to correct the homopolymer errors of the 454 contigs following a standard procedure [79]. The corrected contigs were linked into scaffolds using the program SSPACE [80] with 454, Sanger and Illumina data. Finally, assemblies were gap-closed using GapCloser from the SOAPdenovo 2 package [81] with Illumina data. The statistics of the three genome assemblies are summarized in S5 Table. We assessed the completeness of the three genome assemblies by counting the number of Core Eukaryotic Gene (CEG) using CEGMA [82].

### Experimental determination of nuclear DNA content

Flow cytometry was used to perform accurate measurement of nuclear DNA content in the three apomictic *Meloidogyne* (*M*. *incognita*, *M*. *javanica* and *M*. *arenaria*) as well as in the facultative sexual *M*. *hapla*, compared to internal standards with known genome sizes. *Caenorhabditis elegans* strain Bristol N2 (approximately 200 Mb at diploid state [83,84]) and *Drosophila melanogaster* strain Cantonese S. (approximately 350 Mb at diploid state [85,86]) were used as internal standards. Extraction of nuclei was performed as previously described [87]. Briefly, for each *Meloidogyne* species about two hundred thousand stage 2 juveniles (J2s) were suspended in 2 mL lysis buffer (1mM KCl, 30 mM NaCl, 10 mM MgCl2, 0.2 mM EDTA, 30 mM Tris, 300 mM sucrose, 5 mM sodium butyrate, 0.1 mM PMSF, 0.5 mM DTT, 40 μl Igepal), grinded for 10 min with a Dounce homogenizer and filtered through a 0.20 μm nylon mesh. Subsequently, this 2 mL suspension was overlaid on top of 8 mL suspension buffer (same as lysis buffer except for sucrose, 1.2 M, and without Igepal) so that the tubes were ready for centrifugation (10,000 rpm, 30 min, 4°C) to reduce the level of debris and to pellet nuclei. Supernatant was completely discarded and pelleted nuclei were re-suspended in suspension buffer. Then nuclei suspension was stained, at 37°C for 30 min, with 75 μg/mL propidium iodide and 50 μg/mL DNAse-free RNAse. The same nuclei extraction protocol was performed at the same time on the samples and on the two internal standards. Flow cytometry analyses were carried out using a LSRII / Fortessa (BD Biosciences) flow cytometer operated with FACSDiva v6.1.3 (BD Biosciences) software. Data were analyzed with Kaluza v1.2 software (Beckman Coulter) and cytograms exhibiting peaks for each phase of the cell cycle (G0/G1, S and G2/M) were obtained. Standards and samples were processed both alone and together. Only mean fluorescence intensity of the first peak (arbitrary units), corresponding to G0/G1 phase of the cell cycle of the cytograms, was taken into account to estimate DNA content. In this method [88,89], the amounts of DNA in the *Meloidogyne* samples were determined by interpolating the fluorescence signals generated from the standards using the following equation:

$$Meloidogyne\text{ DNA content }\left(\text{Mb}\right)=\left(\text{G}0/\text{G}1_{Meloidogyne\;\text{sample}}\text{x Standard DNA content}\right)/\text{G}0/\text{G}1_{\text{standard}}.$$

The estimated DNA contents of the *Meloidogyne* samples were calculated by averaging the values obtained from three biological replicates (S8 Fig).

### Search for collapsed duplicated regions

To check whether some nearly identical duplicated genomic regions had been collapsed during the assembly (as previously observed in the *A*. *vaga* genome [5]), we aligned the Illumina PE-reads of each species against their respective genome assembly sequence, using BWA [90] with default parameters. We computed the per base read coverage using BEDtools genomeCoverageBed [91] and plotted the distribution of the per-base coverage depth. This clearly showed 2 peaks for the three species, one systematically at twice the coverage of the first peak (S1 Fig). We calculated the number of bases with per base coverage comprised in the range of the second peak and summed it up to obtain the total size of the duplicated regions that had been collapsed during the assembly.

### Structural annotation

Predictions of protein-coding genes were performed using EuGene 4.1c [92], optimized and tested for *M*. *incognita* on a dataset of 301 non-redundant full-length cDNAs. Translation starts and splice sites were predicted using SpliceMachine [93]. Three datasets of *M*. *incognita* transcribed sequences were provided to EuGene to contribute to the prediction of gene models: i) Sanger ESTs (Genbank 20110419), ii) a dataset of seven Illumina transcriptomes obtained in our lab in a previous study [94], and iii) a dataset of nine Trinity [95] assemblies of RNAseq data, generated in this study (S7 Table, S1 Text). Transcribed sequences were aligned on the genome using GMAP [96]; spliced alignments spanning 80% of the transcript sequence length at a 90% identity cut-off were retained. Similarities to i) *C*. *elegans* release Wormpep221, ii) *G*. *pallida*, release 1.0 [97], and iii) Swiss-Prot release December 2013 (excluding proteins similar to REPBASE [98]) were searched using BLAST [99] and provided to EuGene to contribute to gene modelling. The gene modelling algorithm used the standard parameters for the 4.1c version, except for the fact that i) the gene finding algorithm was applied on both strands independently allowing overlapping gene models, ii) non-canonical GC/donor and AC/acceptor sites were allowed on the basis of transcriptional evidences, iii) a gene model was not allowed to span a gap (‘N’) longer than 1,000 nucleotides, iv) the minimum length of introns was set to 35 nucleotides, and v) the minimum CDS length cut-off was set to 150 nucleotides. For *M*. *arenaria* and *M*. *javanica*, the EuGene pipeline, with models and parameters tuned on *M*. *incognita*, was used to annotate both genomes. Two modifications were applied on the selection of reference datasets i) Swiss-Prot (excluding proteins similar to REPBASE) and the proteome of *M*. *incognita* were used as reference proteomes ii) assemblies of *M*. *arenaria* and *M*. *javanica* RNAseq data were used as sources of transcription evidences.

We annotated ncRNAs using RNAmmer [100], tRNAscan-SE [101], Rfam release 11 [102], and in house scripts to remove redundancy and consolidate results.

### Functional annotation

The predicted protein sequences of *M*. *incognita*, *M*. *javanica*, *M*. *arenaria* and *M*. *hapla* were scanned for the presence of Pfam protein domains using the program PfamScan [103] against the Pfam-A HMM domain library (release 27.0), using default thresholds and parameters. A gene ontology annotation was inferred from the Pfam protein domain annotation using the pfam2go mapping file maintained at the gene ontology portal and generated from the InterPro2GO mapping [104]. Gene ontology terms were also mapped on the generic GO-slim ontology using the GOSlimViewer utility developed as part of AgBase [105].

### Analysis of whole-genome duplication

The duplicated structures of *Meloidogyne* species were estimated by detecting conserved blocks of duplicated genes. The protein sequences of each genome were initially self-blasted to determine a homologous relationship with an e-value threshold of 1e^-10^. Conserved blocks of duplicated genes were detected based on the gene locations in the genome using MCScanX [29] with default parameters. We required at least 3 collinear genes pairs for MCScanX to form a block. Using the perl script “add_ka_and_ks_to_colinearity.pl” included in the MCScanX package, we calculated Ks values for each homologous gene pairs between duplicated blocks. The median Ks value was considered a representative of the divergence between duplicated regions.

We used a custom python script (S2 Text) to compute the pairwise nucleotide identity between collinear blocks for each species. Briefly, pairs of duplicated genomic regions were extracted according to the GFF3 positions of their first and last collinear genes. They were then aligned using NUCmer from MUMmer v3.23[106] with default parameters. We then filtered out sub-alignment shorter than 50 nt (delta-filter -l 50) and summarized alignment using the dnadiff program from the MUMmer package. The average identity at the nucleotide level between duplicated regions was obtained from the output of dnadiff. Identity within coding and non-coding sequences was obtained by masking coding or non-coding sequences in each duplicated region before NUCmer alignment using BEDtools maskFastaFromBed v2.17.0 [91].

To analyze synteny conservation between genomes, we concatenated all the inter-/intra-species BLAST hits (e-value threshold of 1e^-10^) of *M*. *incognita*, *M*. *javanica* and *M*. *arenaria* and *M*. *hapla* protein sequences and fed MCScanX with this pooled BLAST result as well as with information on the location of the corresponding genes in the respective genomes, as recommended in the MCScanX manual for multi-species comparisons. The *M*. *floridensis* genome had to be discarded from this comparative analysis because only one pair of regions composed of 12 genes block was detected for this genome preventing any large-scale analysis of conserved synteny. We required at least 3 collinear genes pairs for MCScanX to detect a block. We parsed the results of the collinearity analysis between genomes of *Meloidogyne* species (HTML files output by MCScanX) to extract collinear genes forming duplicated regions conserved between *Meloidogyne* species. We used those homologous collinear genes to perform phylogenomics analyses (see below).

### Determination of fragmentation bias

For each pair of duplicated regions, the genes present on each region were counted and the number of genes that were present in the ancestor of these two regions was calculated as the total number of genes on the two collinear regions minus the number of gene pairs. We then compared the number of genes in each region to the number of estimated genes in the ancestral region to statistically determine whether one region had retained significantly more genes than the other within a pair.

### Alignments, phylogenies and topologies searching

First, protein sequences were aligned using MUSCLE v3.8.31 [107,108]. Second, protein alignments were back translated into codon alignment using PAL2NAL v.14 [109] with the ‘nogap’ option. Third, codon alignments were trimmed using GBLOCKS [110] with default options. The fittest model of nucleotide evolution was searched using the function ModelTest as implemented in the R package phangorn [111]. We then used PhyML [112] (-d nt -b -4 -m GTR -f e -t e -v e -a e -s BEST) to build maximum likelihood phylogenies with SH-like branches support on these pruned alignments. We rooted the phylogenies using the midpoint function of R package phangorn. For the rest of the analyses, we only retained the trees in which *M*. *hapla* displayed an outgroup position relative to the other *Meloidogyne* species in the midpoint-rooted topologies. Tree topologies were classified and counted using a custom R script. Phylogenetic tree figures were formatted and edited using EvolView [113].

### Identifying gene copies subject to positive selection

To compute the Ka / Ks ratios per pair of collinear homologous genes, we used the Nei-Gojobori method [114] implemented in MCScanX. We eliminated all cases where 0.01<Ks<1 to discard genes that were either evolving extremely slowly or extremely rapidly and could potentially yield erroneous Ka/Ks estimates. We performed tests of episodic diversifying selection (EDS, a form of positive selection) using the random effects branch-sites model [33] implemented in the HYPHY package [115]. We looped the branchSiteREL.bf script over the 1,735 multi-sequence alignments and their respective ML midpoint rooted trees. Each alignment contained at least one collinear protein-coding gene for all three apomictic species and *M*. *hapla* and a duplicate in at least one asexual species. We chose the adaptive version of BSRE and allowed branch-site variation in synonymous rates. Branch with length less than 0.01 were not considered because ω rate classes cannot be inferred reliably for very small branches (< 0.01).

### RNAseq sample preparation and sequencing

Total RNAs were extracted from 4 *M*. *incognita* developmental stages (pre-parasitic J2s, parasitic J3-J4, adult females and eggs) using TRIzol Reagent (Invitrogen); three independent biological replicates were performed for each stage. Total RNA quality and quantity were assessed by a 2100 Bioanalyser (Agilent technologies). Samples with RNA integrity number (RIN) over 8.5 were kept for cDNA library construction, except for eggs samples for which RIN ranged between 6 and 7. An input of 100 ng total RNA was provided to construct cDNA libraries *via* the Ovation Universal RNAseq system (Nugen technologies). To eliminate unwanted rRNA transcripts, we designed 101 InDA-C primers to target *M*. *incognita* 28S and 18S transcripts for depletion. The 12 cDNA libraries (4 stages x 3 replicates) were quantified and equilibrated to 4 nM using Kapa QPCR (Kapa Biosystems). Finally, multiplexed libraries were sequenced on an Illumina NextSeq 500 sequencer on two High 150 flow cells (400M PE75 reads), on the UCA Genomix sequencing platform of Nice Sophia-Antipolis.

### RNAseq reads cleaning, mapping and counts

The quality of the raw read fastq files were manually checked using FastQC and the following series of filters were applied to all the files. We first eliminated possible remaining ribosomal RNA contamination using SortMeRNA [116]. We then used PRINSEQ [117] to trim sequence ends with quality score lower than 28, and only kept reads with an overall score >28 and a length >60 nucleotides. We aligned the cleaned reads to the *M*. *incognita* indexed genome assembly using the STAR 2-pass procedure [118].

We used RSEM [119] to estimate read counts, transcripts per million (TPM) as well as fragments per kilobase per million mapped reads (FPKM) for the *M*. *incognita* predicted protein-coding genes. RSEM takes into account multi-mapped reads and assigns them proportionally to the different loci according to probabilities estimated based on uniquely mapped reads.

### Identification of homoeologous gene copies with different gene expression patterns

We transformed the raw FPKM values in log10 (FPKM+1) values. To avoid the risk of spurious read counts due to low complexity regions in transcripts, we eliminated all the transcripts that had more than 1/3 of their length covered by low-complexity regions, as measured by RepeatMasker [120]. We also filtered out genes showing too much variability between replicates and showing an inside-replicate coefficient of variation of log10(FPKM+1) higher than 0.8. We finally averaged expression over each triplicate, and filtered out genes with log10(FPKM+1) mean expression values lower than 0.3 in all conditions, as this corresponded to low signal.

We then clustered genes according to their expression pattern in the four conditions. To be as robust and conservative as possible, we clustered together genes showing the same relative expression patterns in the four conditions, i.e. genes whose expression values are ranked in the same order between the four conditions, resulting in 24 (= 4!) groups. Homoeologous genes from a same pair that were located in two different gene expression clusters were considered as having diverged expression patterns.

### Transposable elements annotation and analysis

#### TE detection and annotation

Repeat annotation was performed using the REPET pipelines TEdenovo and TEannot [121]. The TEdenovo pipeline was used to search for repeats in the contigs of the four *Meloidogyne* genomes pooled together (*M*. *incognita*, *M*. *javanica*, *M*. *arenaria*, *M*. *hapla*) to ensure that the annotation results were based on the same *de novo* predictions and were comparable between species. The high scoring segments pairs detection was performed by aligning all genomes against themselves using BLASTER [122] with the following parameters: identity >90%, High Scoring segments Pairs (HSP) length >100b & <20kb, e-value <1e^-300^. The LTRs retrotransposons were searched using LTRharvest [123] with the following parameters: LTR similarity >90% min LTR size >100bp & <1000bp. The repetitive HSPs identified from the BLASTER output were clustered using Recon [124], Grouper [122] and Piler [125]. The predictions from LTRharvest were clustered using the MCL algorithm. The consensus for each cluster are obtained using MAP [126] and classified with PASTEC Classifier [127]. The unclassified consensus sequences (noCAT) were filtered out to keep only consensus sequences built from at least 10 sequences in the cluster. The library of 10,535 consensus sequences obtained with TEdenovo was used for separate annotation of each genome using TEannot with the same parameters. The alignment of the reference consensus TE sequences on the genome was made using Blaster, CENSOR [128] and RepeatMasker (http://www.repeatmasker.org/). The results of the three methods were concatenated and MATCHER [122] was used to remove overlapping HSPs and make connections with the "join" procedure. Using in-house perl script, we retrieved TE annotation with >80% sequence identity to consensus sequences & length >150 nucleotides.

#### TE-related Pfam domains

A published list of 124 Pfam domains associated with TE [129] was combined with the list of TE-related Pfam domains included in the LTR Digest software [130], resulting in a non-redundant list of 129 TE-related Pfam domains. The *Meloidogyne* species analyzed in the present article possessed 28 of these 129 TE-related domains. The compared abundance of these 28 domains in the three apomictic *Meloidogyne* is presented in S4 Table.

## Supporting information

S1 Fig Per base coverage distribution of Illumina PE-reads mapped on asexual *Meloidogyne* genomes. Two peaks are observed. One corresponding to the average coverage of the genome and a second at ~ twice the coverage of the first peak. This second peak shows that a substantial portion of the genomes has a twice higher coverage. Sequences with this twice higher coverage may represent nearly identical duplicated sequences merged into single sequences in our assembly because they are almost identical.
(PDF)

S2 Fig Distribution of % identity between pairs of duplicated regions in the asexual *Meloidogyne*. Counts of pairs of duplicated genomic blocks (y-axis) as a function of their % identity (x-axis), in *M*. *incognita* (M. inc), *M*. *javanica* (M. jav) and *M*. *arenaria* (M. are).
(PDF)

S3 Fig Vista plot of alignment of a pair of duplicated blocks taken as an example for *M*. *incognita*. To illustrate the divergence between pair of duplicated collinear blocks formed by MCScanX, we aligned (using LAGAN: at http://genome.lbl.gov/vista/index.shtml), 2 collinear blocks of *M*. *incognita*. Block located on scaffold “Minc3s00001” from position 286,600 to 315,888 is aligned against its homoeolog block located on scaffold “Minc3s00990” from position 3,627 to 27,194. Percentage identity (between 50 and 100%) is plotted against the scaffold sequence (light grey line) for local alignment with 70% coverage and a minimum of 100 bp. Location of homologous genes is indicated by dark grey arrows. Identity is in salmon for conserved non-coding sequences (CNS) and in light slate blue for protein-coding sequences.
(PDF)

S4 Fig Collinearity plotted against average Ks per pair of duplicated blocks. For each pair of collinear blocks collinearity is computed as the fraction of collinear genes within a pair of blocks and Ks is obtained using the add_ka_and_ks_to_collinearity.pl script of the MCScanX package.
(PDF)

S5 Fig Circos and corresponding phylogenies for genomic regions in one copy in *M*. *hapla* and two or more copies in asexual *Meloidogyne*. In each Circo (unit = kb), color codes between collinear blocks are as follows. Collinear orthologs between *M*. *hapla* and any of the three asexuals species in grey. Collinear ‘homoeologs’ within asexual species in purple. Collinear orthologs between *M*. *arenaria* and *M*. *javanica* in green. Collinear orthologs between *M*. *arenaria* and *M*. *incognita* in yellow. Collinear orthologs between *M*. *incognita* and *M*. *javanica* in red. The outer scaled blue line represents gene density on scaffolds. The corresponding ML phylogenies performed on the concatenated alignments per blocks are given below each Circo.
(PDF)

S6 Fig Mitochondrial phylogeny with branch lengths. Mitochondrial phylogenetic tree of *Meloidogyne* with actual branch lengths showing high identity level between apomictic root-knot nematodes.
(PDF)

S7 Fig Example of episodic diversifying selection detected in a phylogenetic tree. Example of a duplicated gene copy harboring signature of episodic diversifying selection as detected by the Branch-Site REL model. The branch leading to the gene Mjav1s00402g005840 is inferred to be under (or to have underwent) episodic diversifying selection (corrected p = 0.013). The hue of each color indicates strength of selection, with primary red corresponding to *ω* > 5, primary blue to *ω* = 0 and grey to *ω* = 1. The width of each color component represents the proportion of sites in the corresponding class. Thicker branches have been classified as undergoing episodic diversifying selection by the sequential likelihood ratio test at corrected p ≤ 0.05. *ω*: the inferred rates of non-synonymous mutations / rate of synonymous mutations ratio.
(PDF)

S8 Fig Relative DNA staining in nuclei of *Meloidogyne* spp. Example of an unfiltered cytogram (arbitrary units) where nuclei were processed alone in a single acquisition: **(A)** *M*. *incognita* sample and **(B)** *C*. *elegans* standard. **(C)** Cytogram example obtained after gating on G0/G1 nuclei (arbitrary units) from each *Meloidogyne* species (*M*. *hapla*, *M*. *incognita*, *M*. *javanica* and *M*. *arenaria*) when processed mixed altogether with an internal standard (*D*. *melanogaster*: approximately 350 Mb).
(PDF)

S1 Text Supplementary methods, results and discussion. (PDF)

S2 Text Python script “extractColBlock.py”. (PDF)

S1 Table Species list and sequence accession number for the mitochondrial phylogeny. (PDF)

S2 Table Functional annotation of genes under positive selection (Ka/Ks >1). (XLSX)

S3 Table Functional annotation of genes under episodic diversifying selection (EDS). (XLSX)

S4 Table Abundance of Pfam domains associated to transposable elements. (PDF)

S5 Table Statistics of genome assemblies. (PDF)

S6 Table Statistics of genome reads used for genome assemblies. (PDF)

S7 Table Statistic of transcriptome reads used for gene models prediction. (PDF)
