# Supplementary figures and images for: Hybridization and polyploidy enable genomic plasticity without sex in the most devastating plant-parasitic nematodes
Source: PLoS Genet. 2017 Jun 8;13(6):e1006777. doi: 10.1371/journal.pgen.1006777 (PMC5465968; doi:10.1371/journal.pgen.1006777)

*M. incognita*

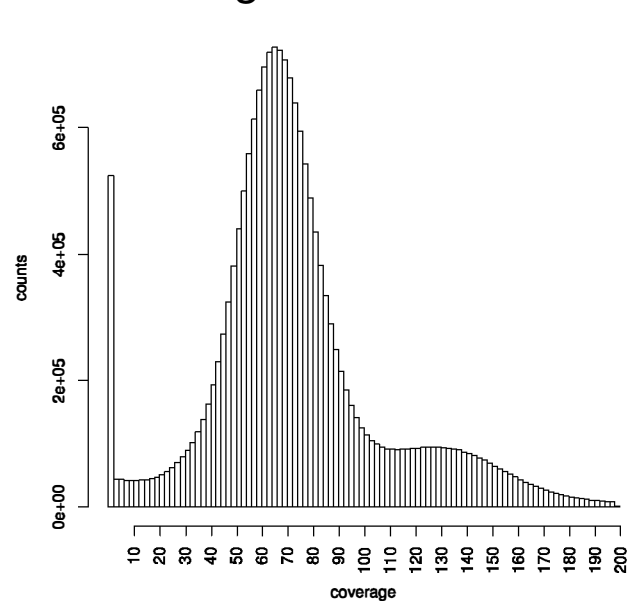

*M. javanica*

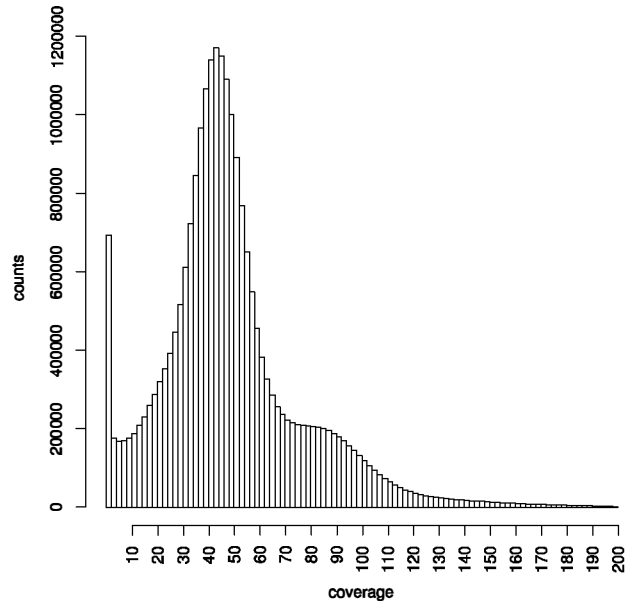

*M. arenaria*

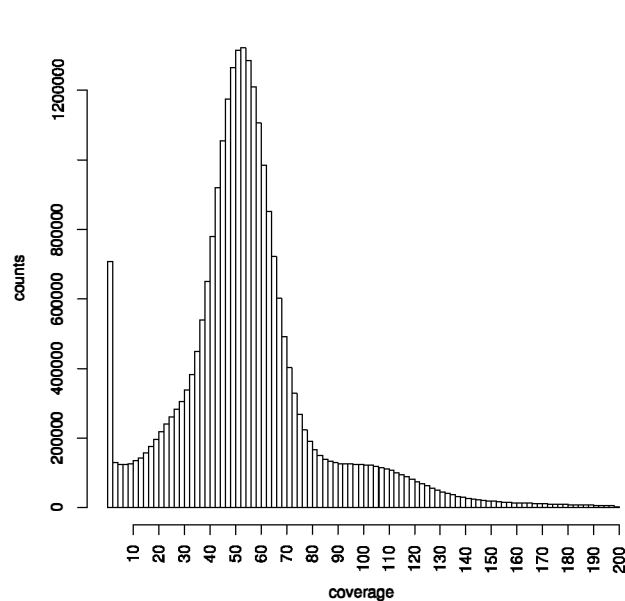

Supplement: S1 Fig — Two peaks are observed. One corresponding to the average coverage of the genome and a second at ~ twice the coverage of the first peak. This second peak shows that a substantial portion of the genomes has a twice higher coverage. Sequences with this twice higher coverage may represent nearly identical duplicated sequences merged into single sequences in our assembly because they are almost identical. (PDF) [file pgen.1006777.s001.pdf]

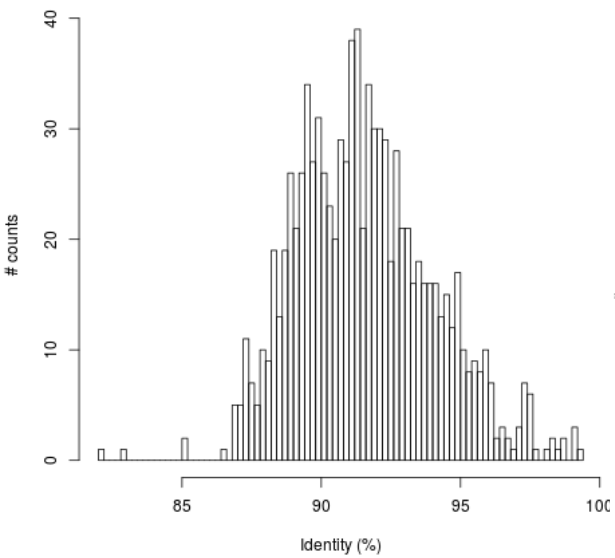

*M.inc*

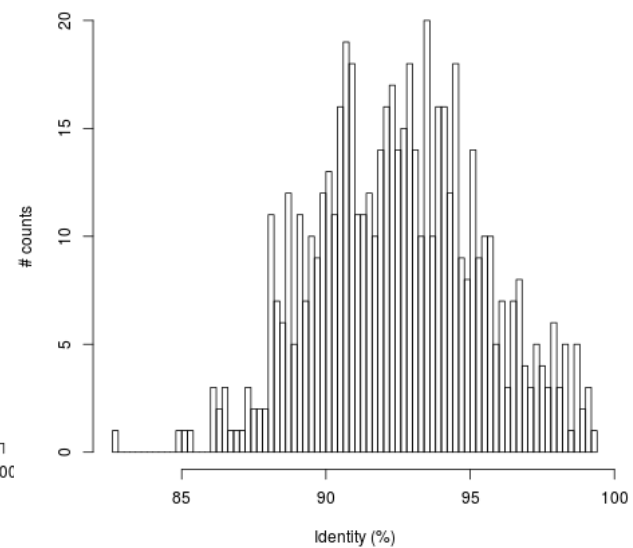

*M.jav*

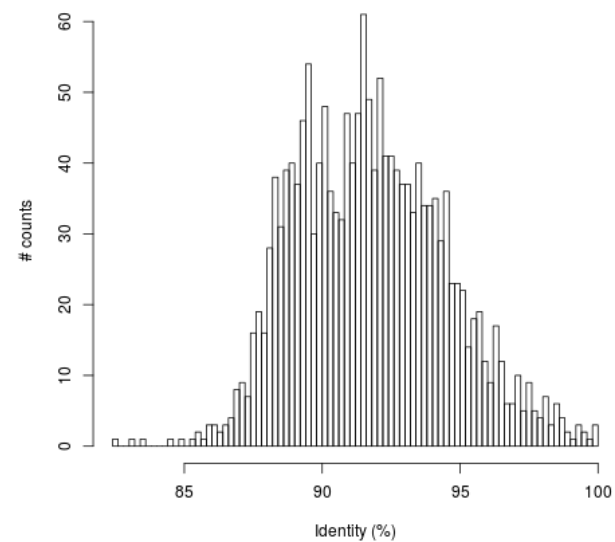

*M.are*

Supplement: S2 Fig — Counts of pairs of duplicated genomic blocks (y-axis) as a function of their % identity (x-axis), in M. incognita (M. inc), M. javanica (M. jav) and M. arenaria (M. are). (PDF) [file pgen.1006777.s002.pdf]

*M. incognita*

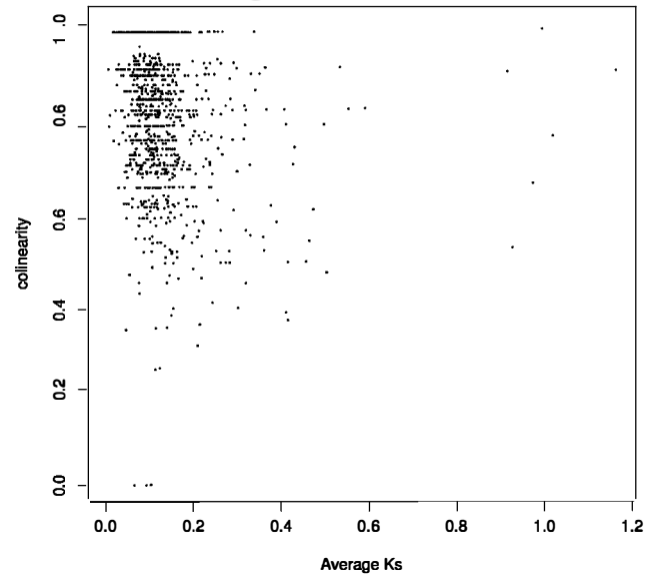

*M. javanica*

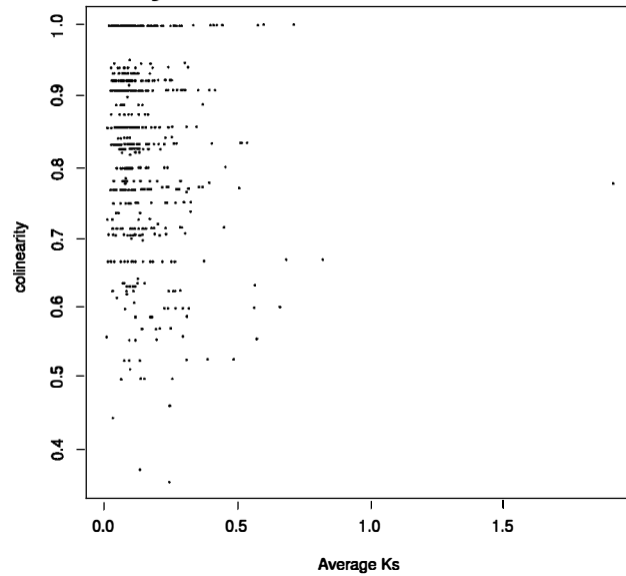

*M. arenaria*

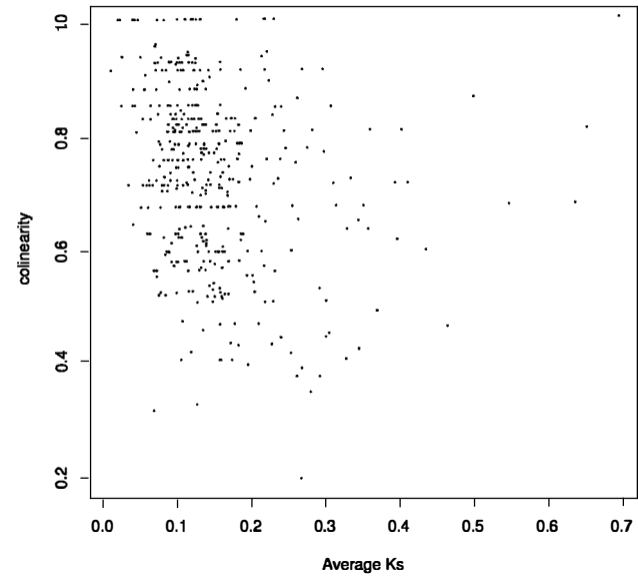

Supplement: S4 Fig — For each pair of collinear blocks collinearity is computed as the fraction of collinear genes within a pair of blocks and Ks is obtained using the add_ka_and_ks_to_collinearity.pl script of the MCScanX package. (PDF) [file pgen.1006777.s004.pdf]

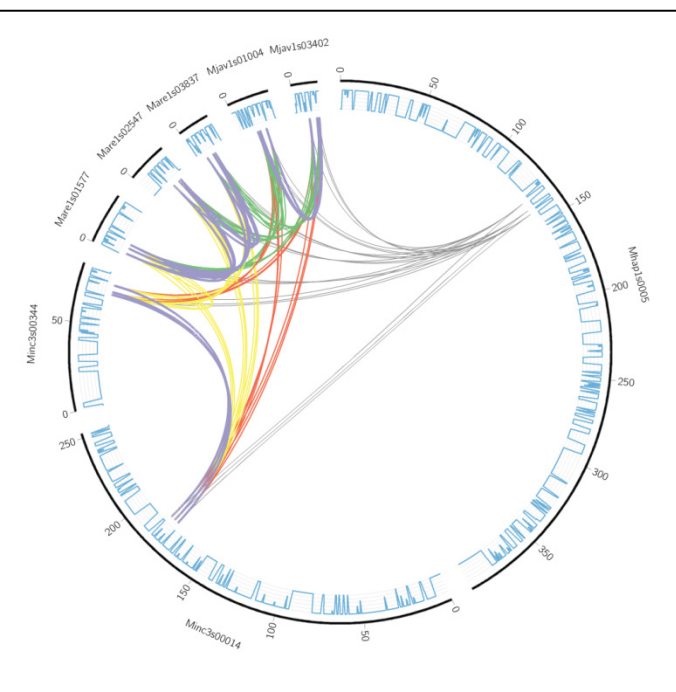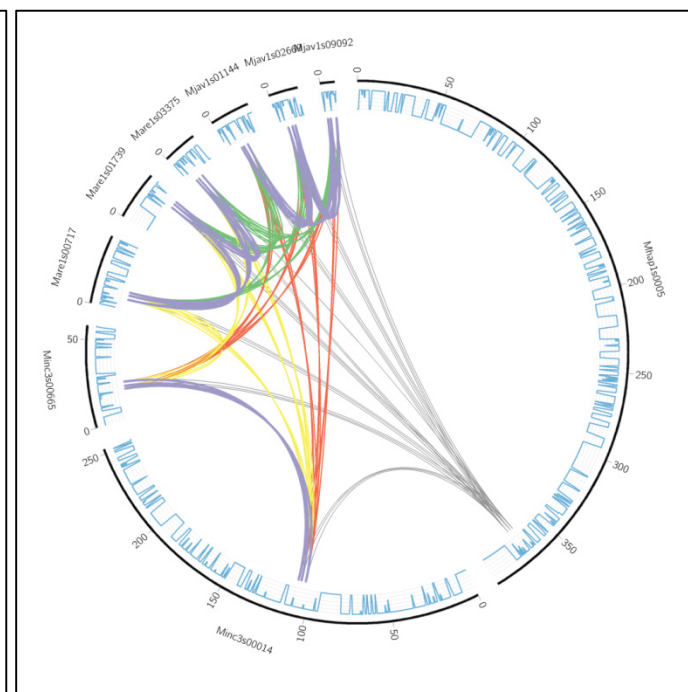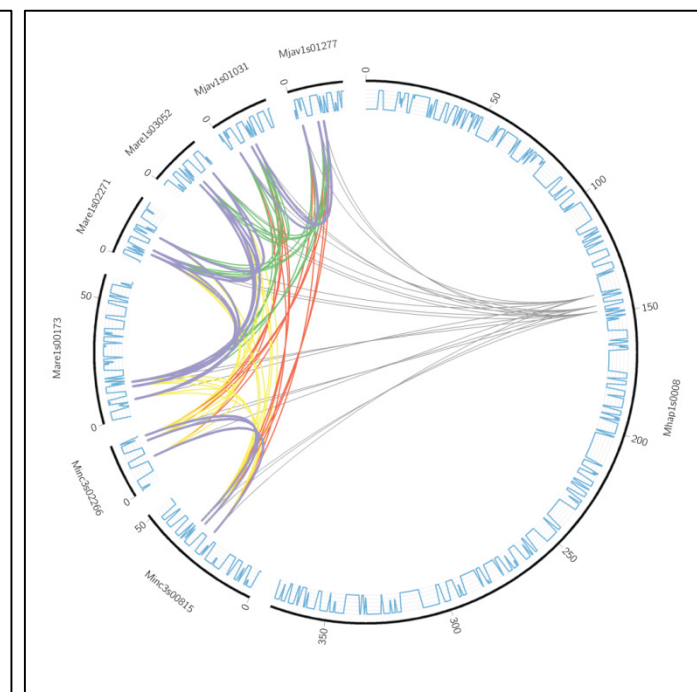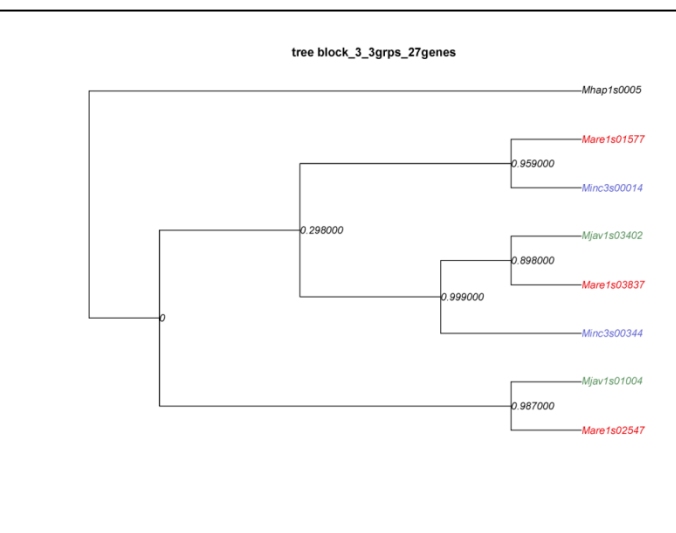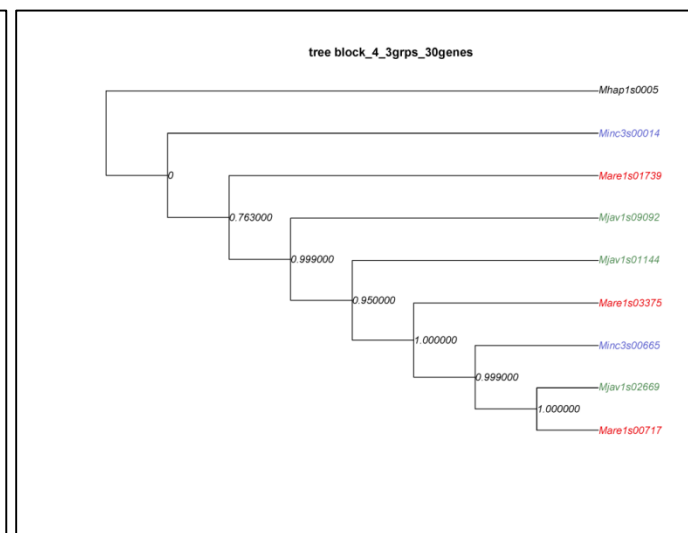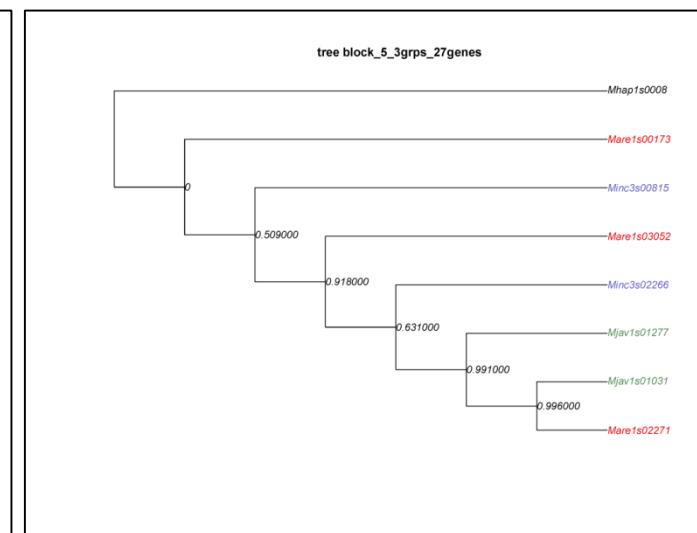

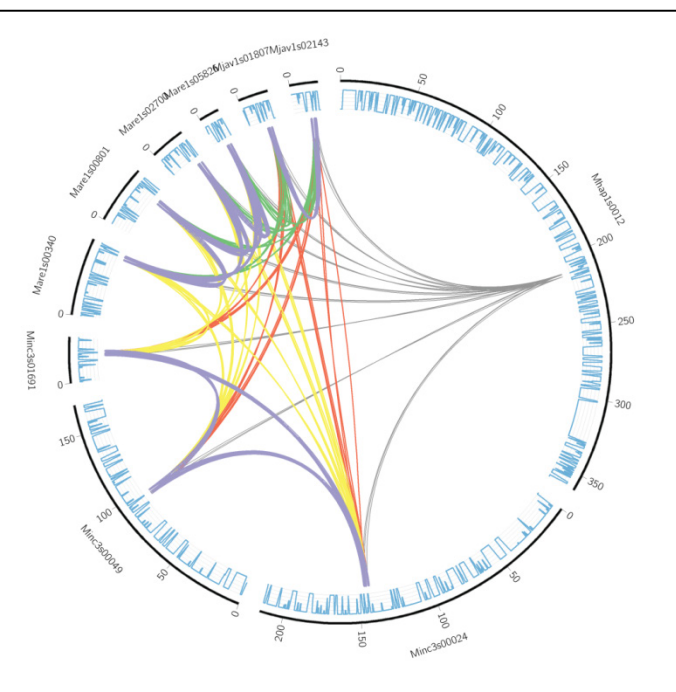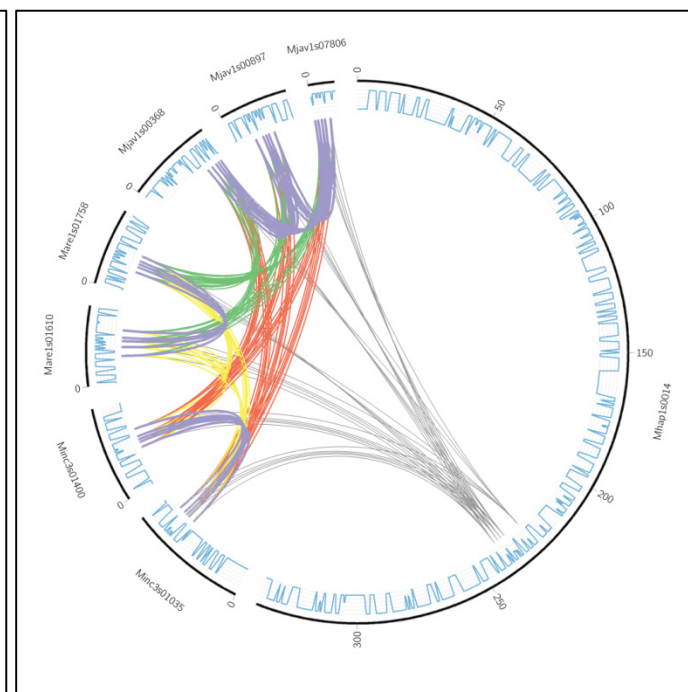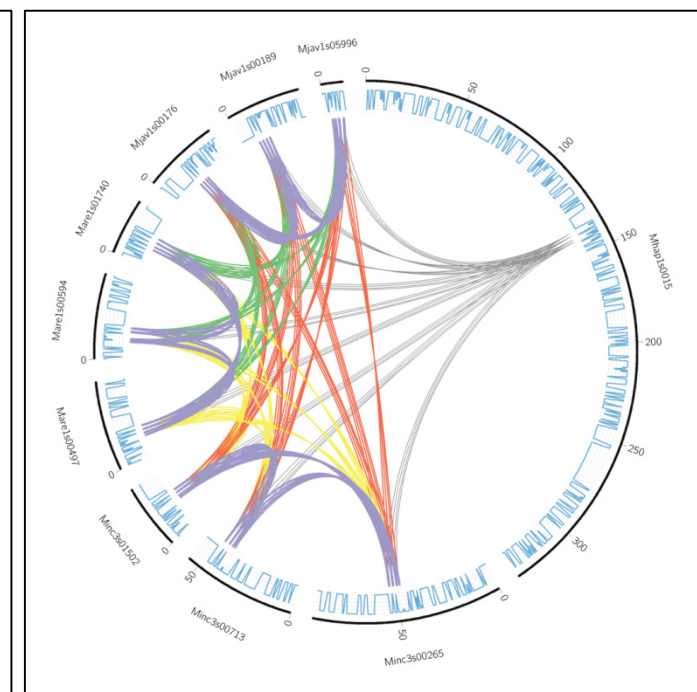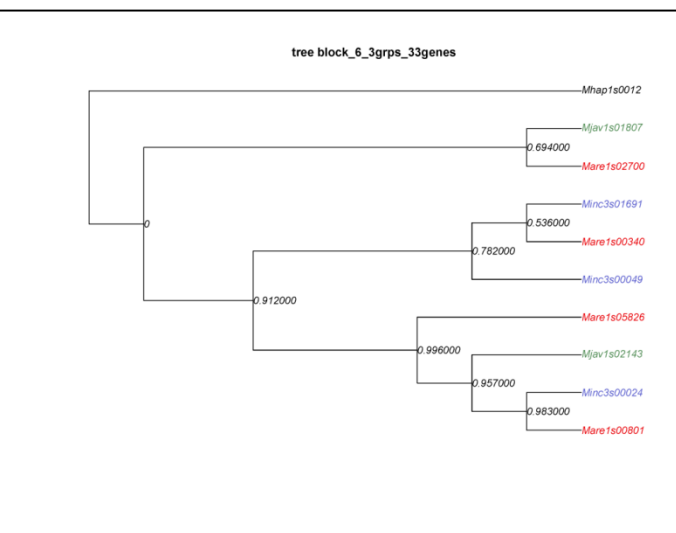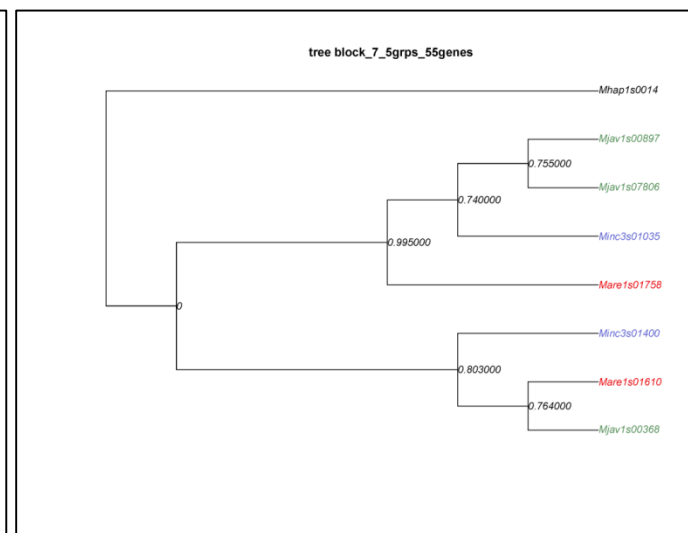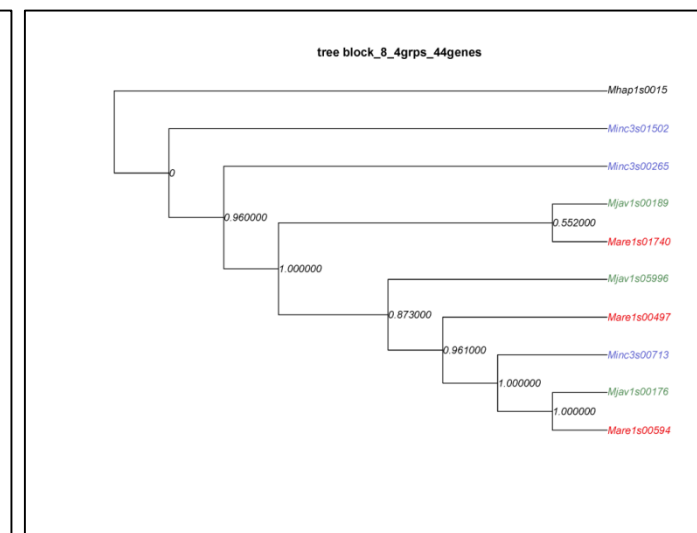

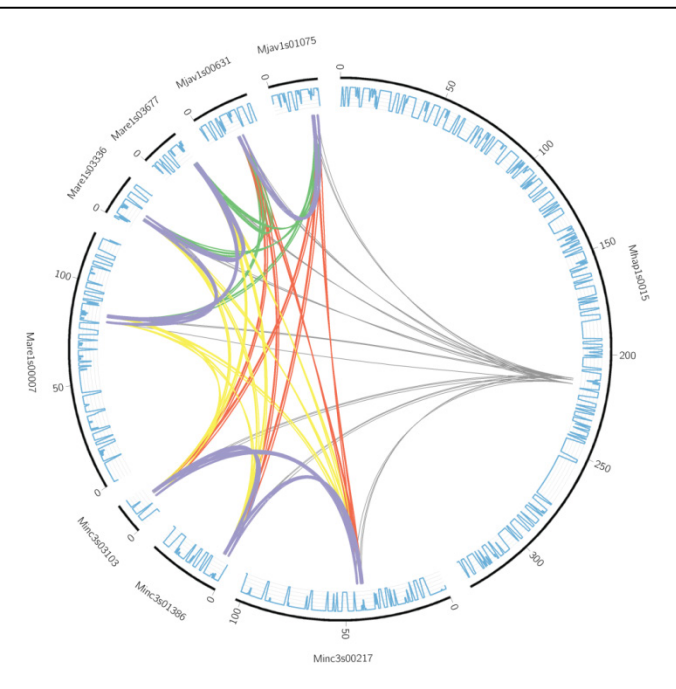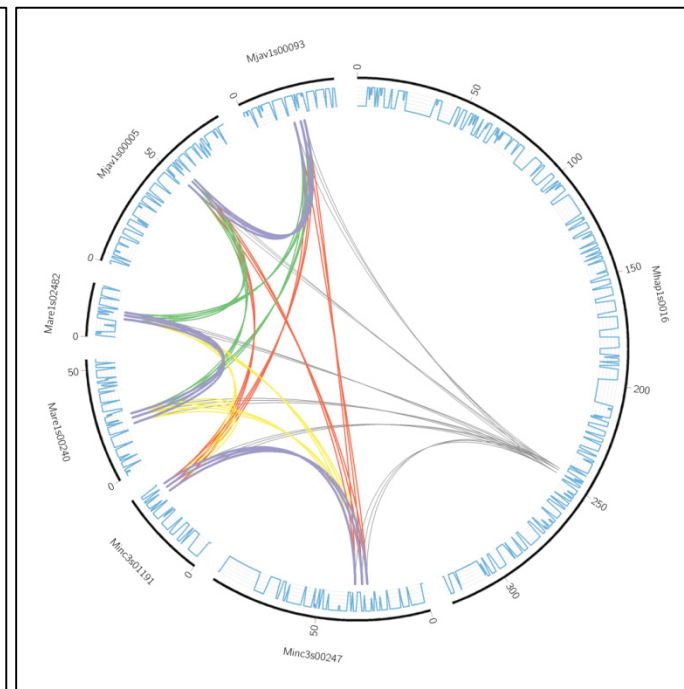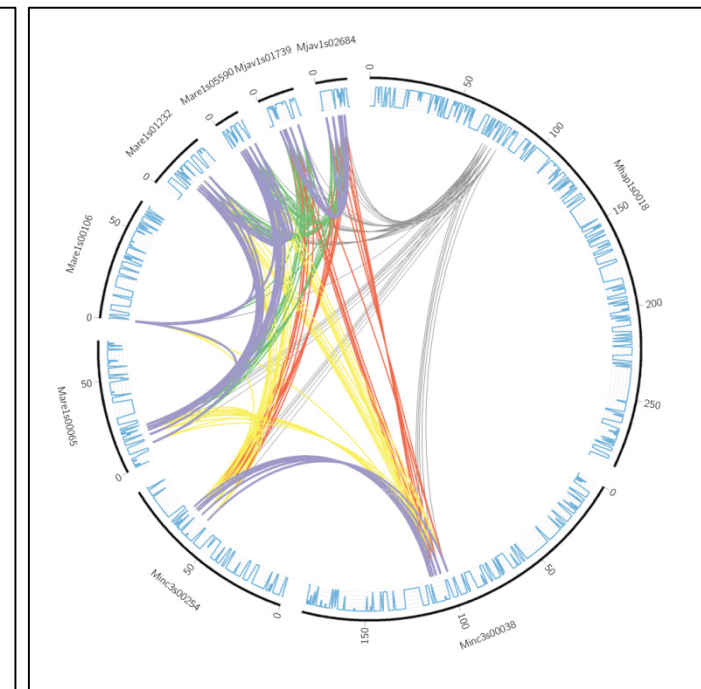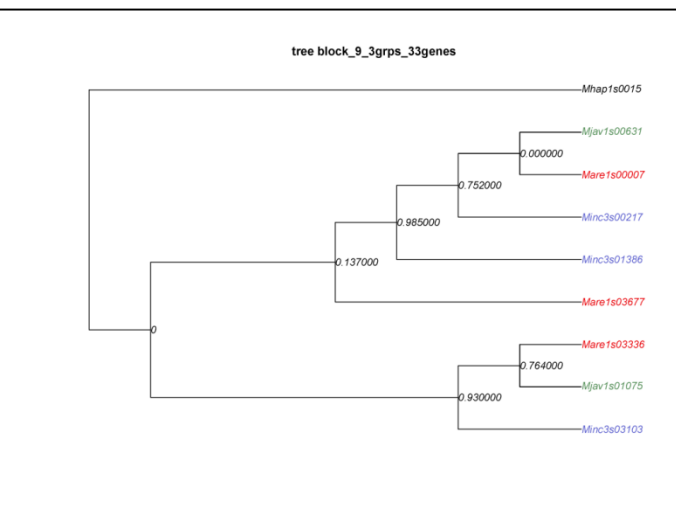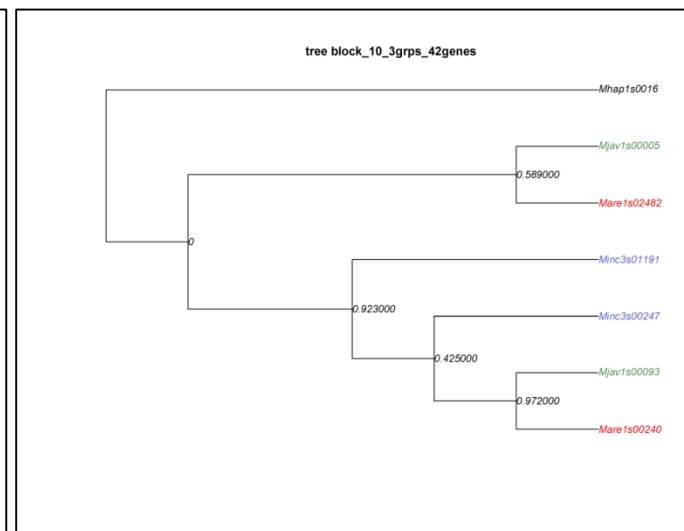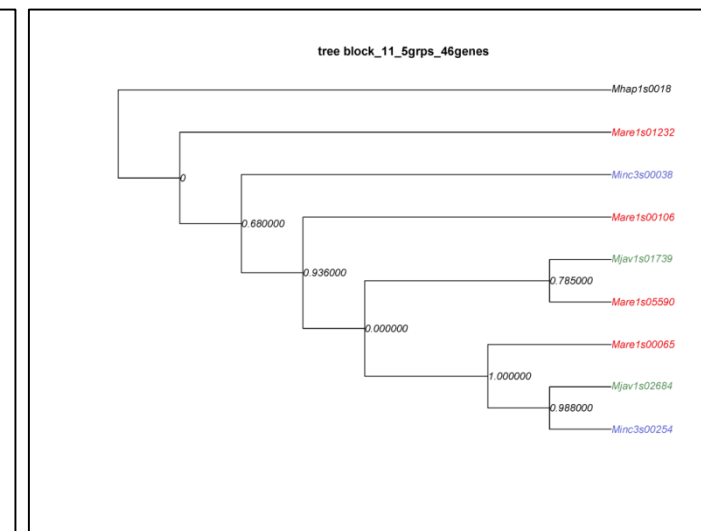

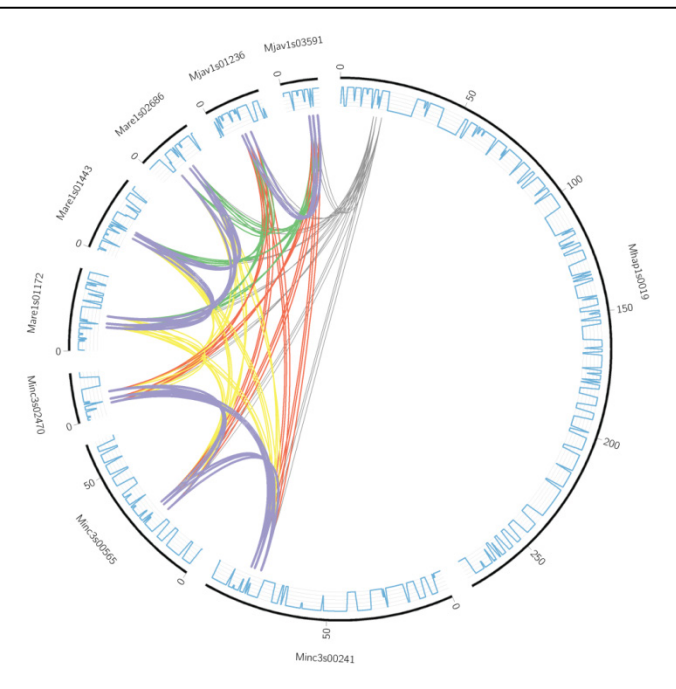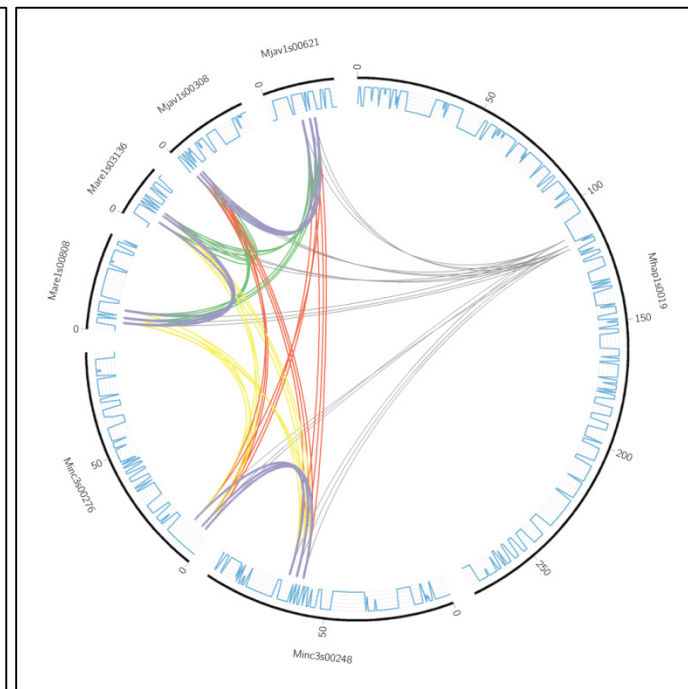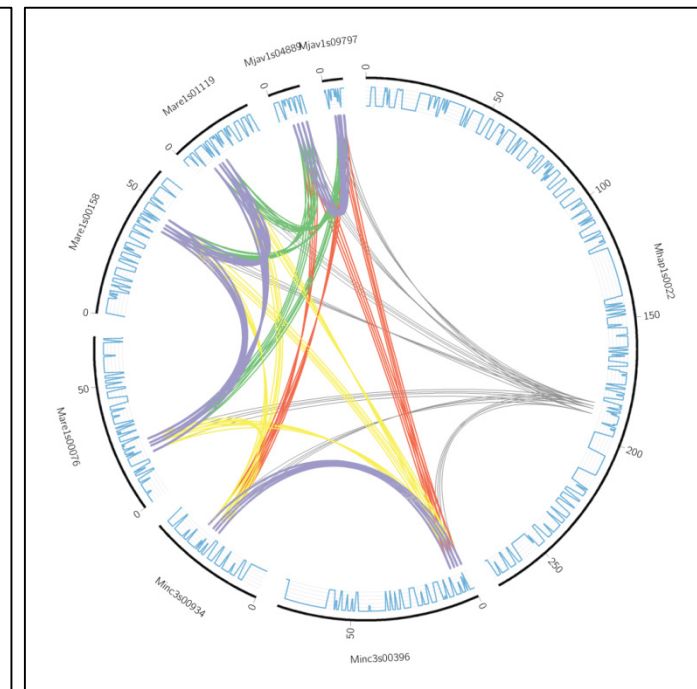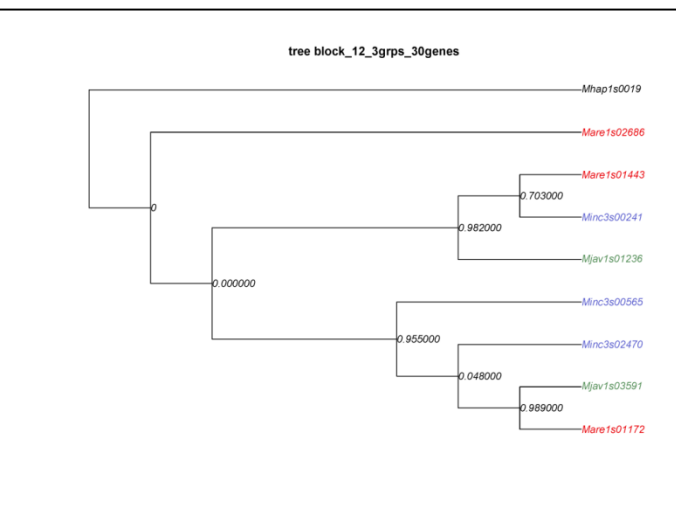

No tree

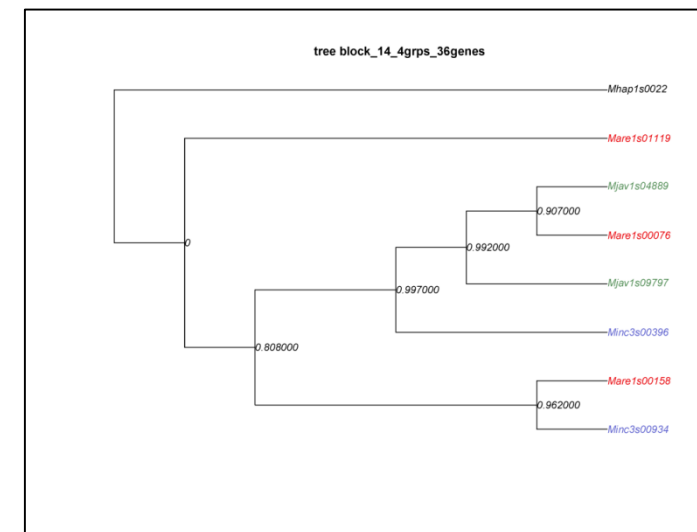

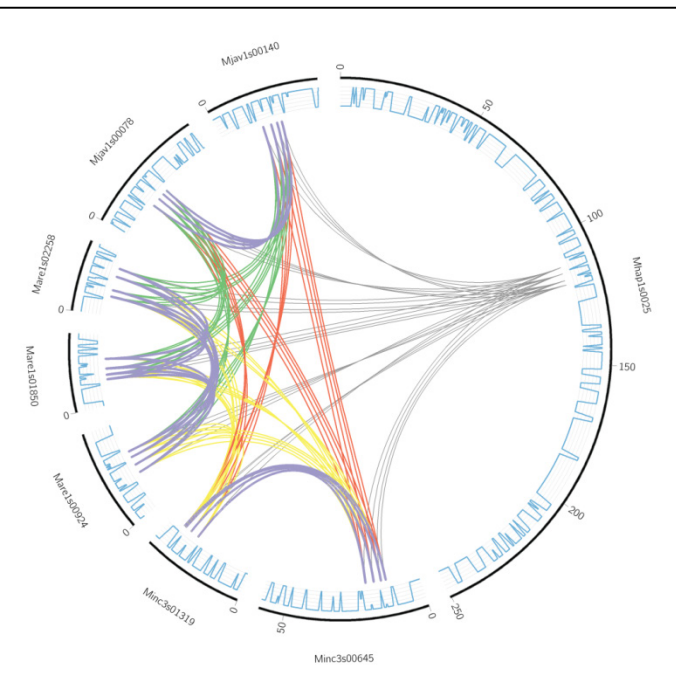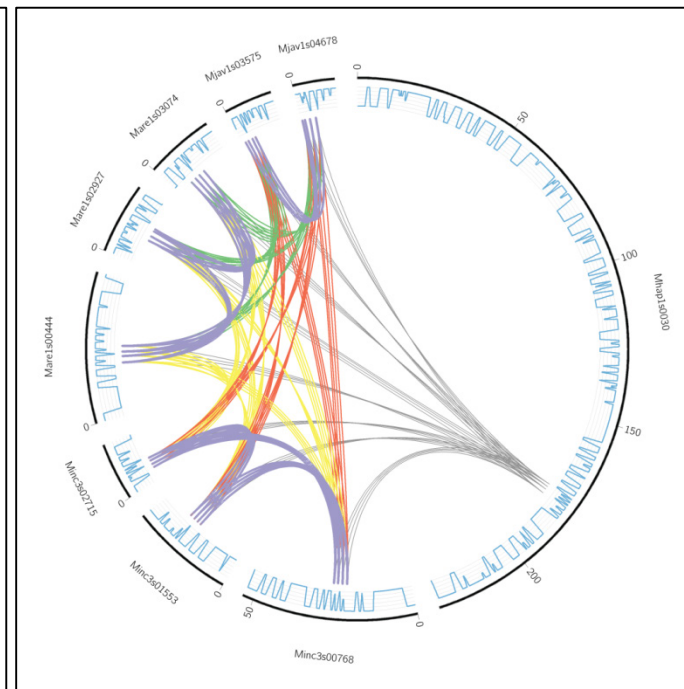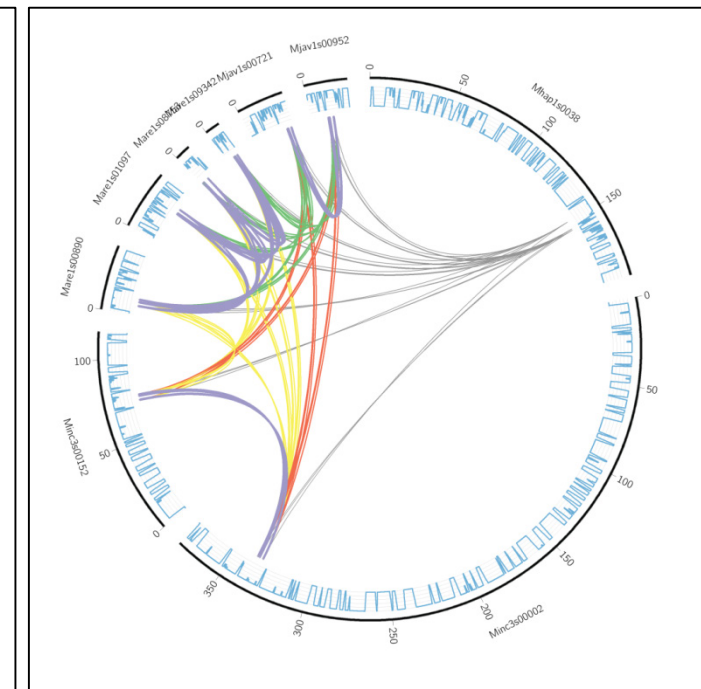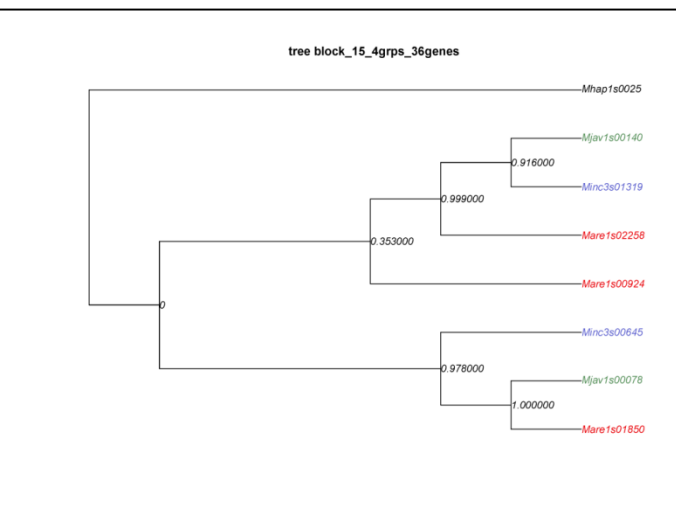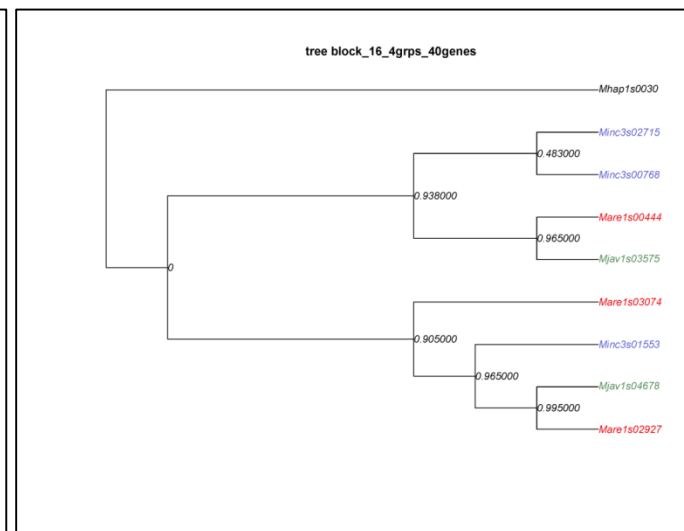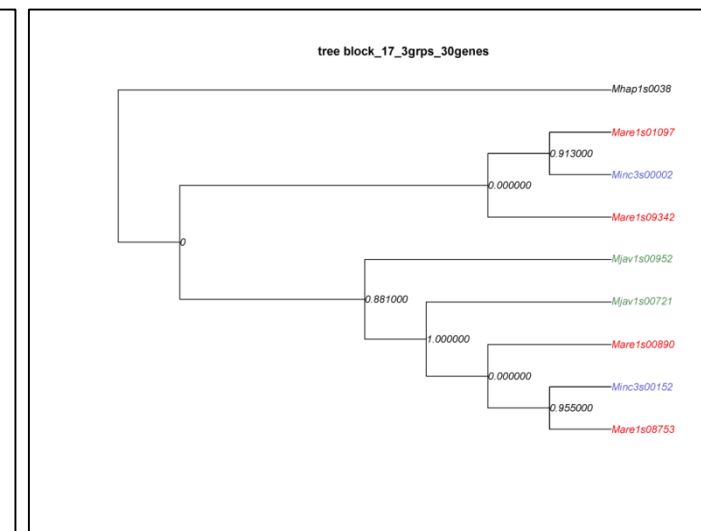

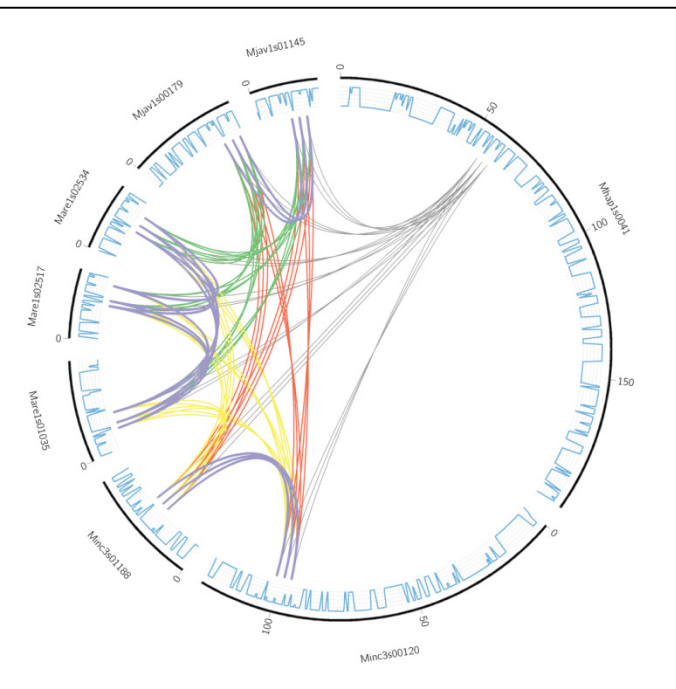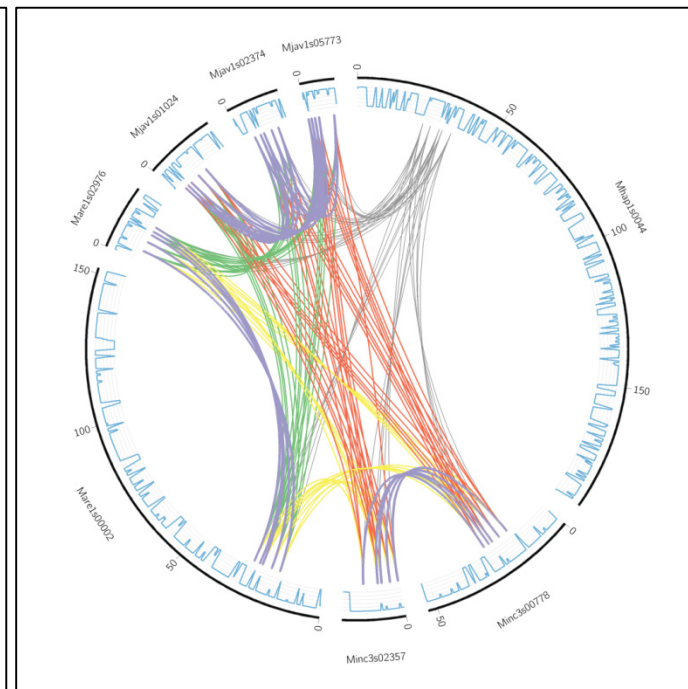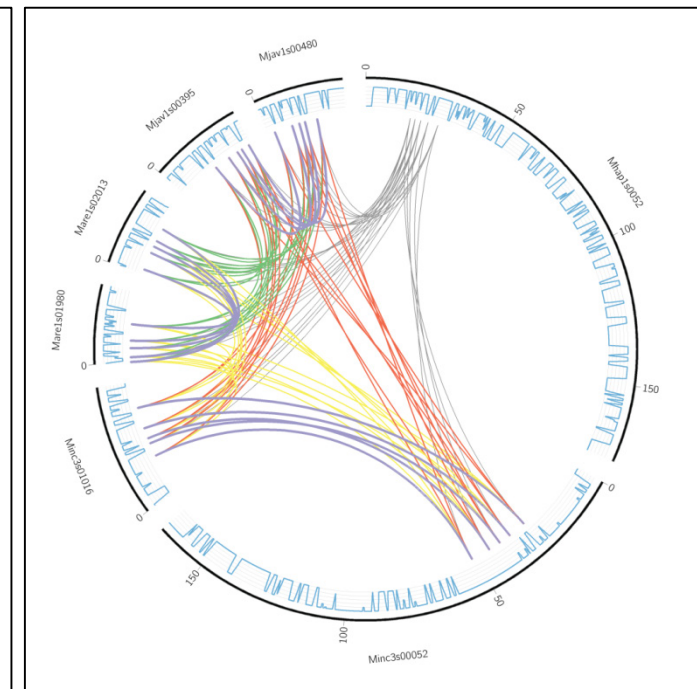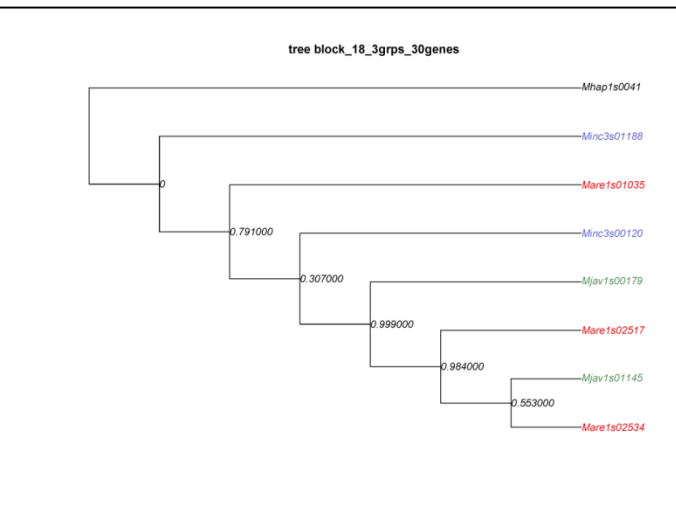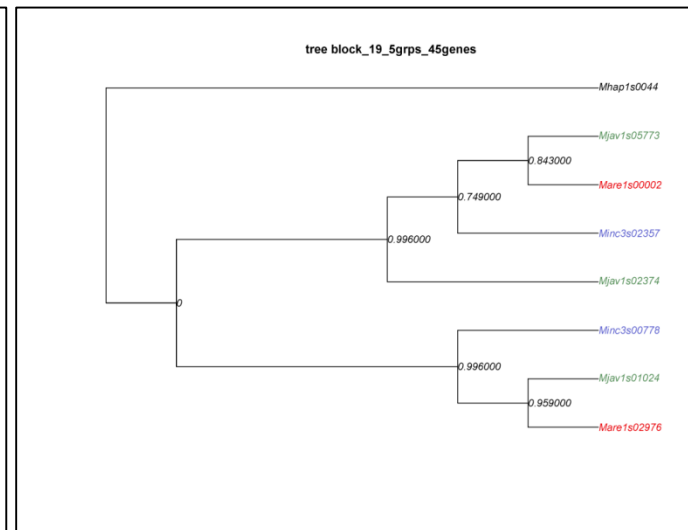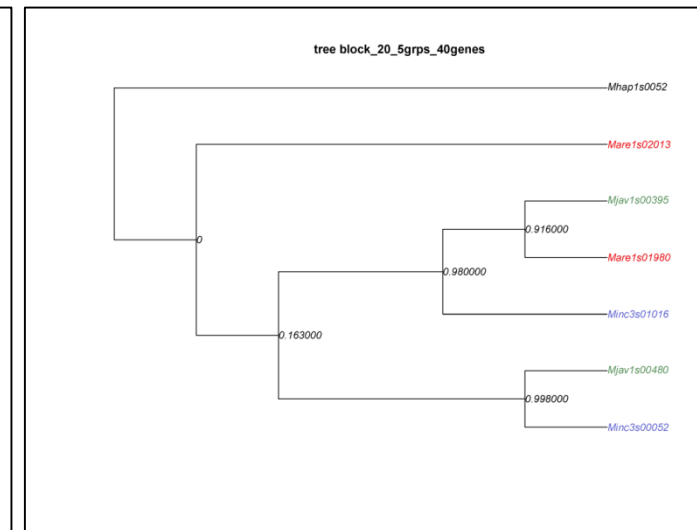

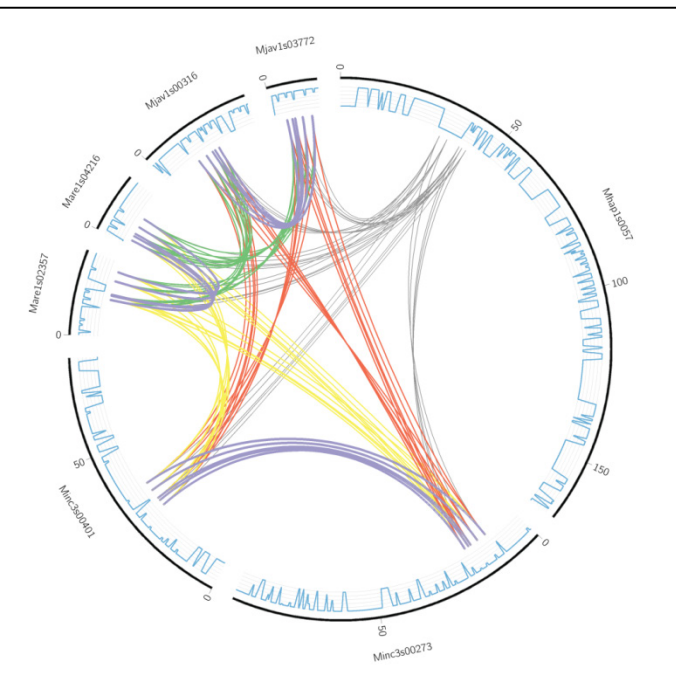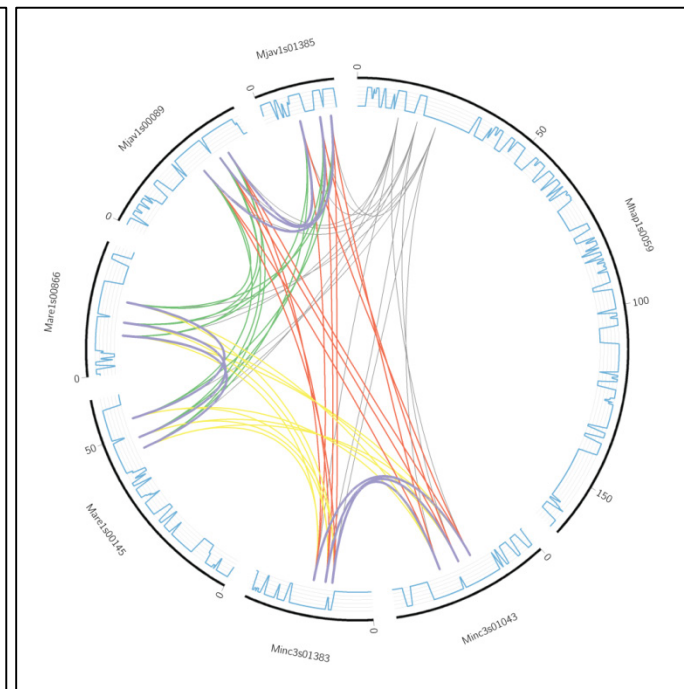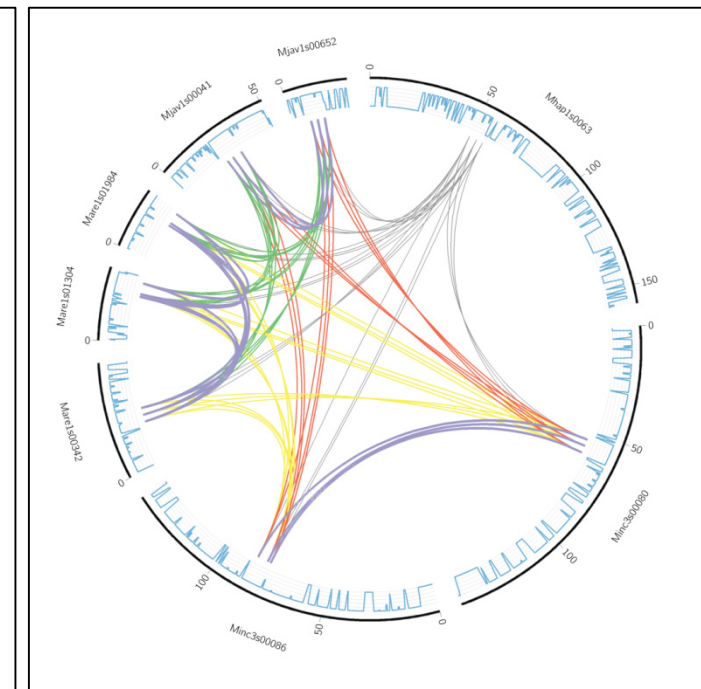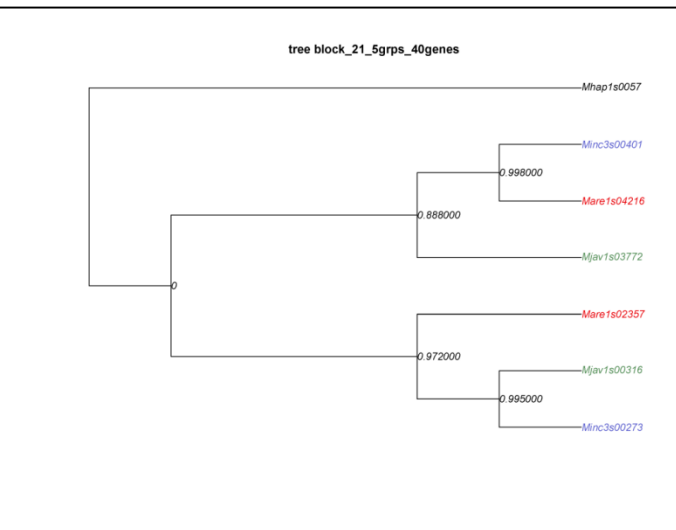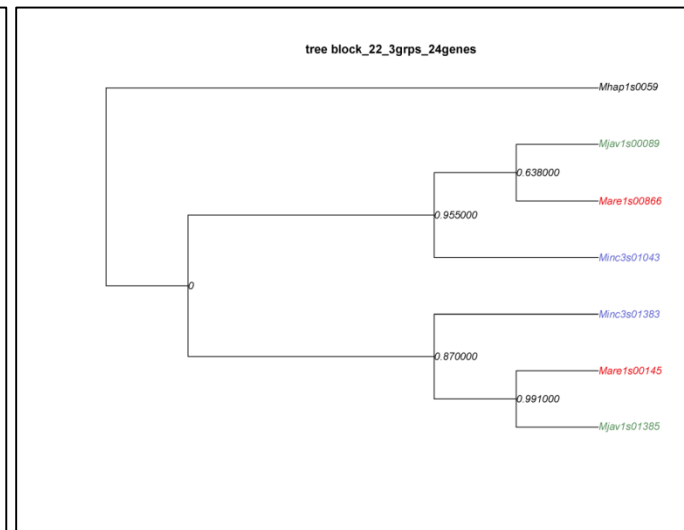

No tree

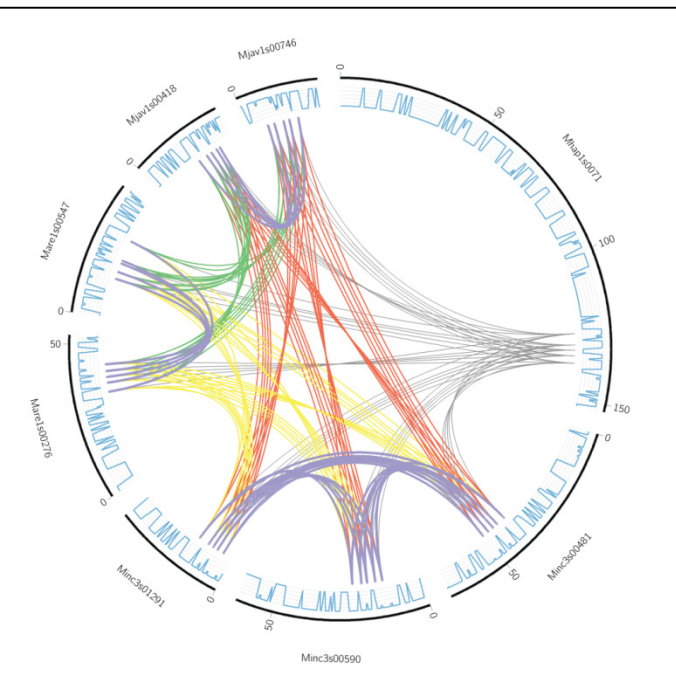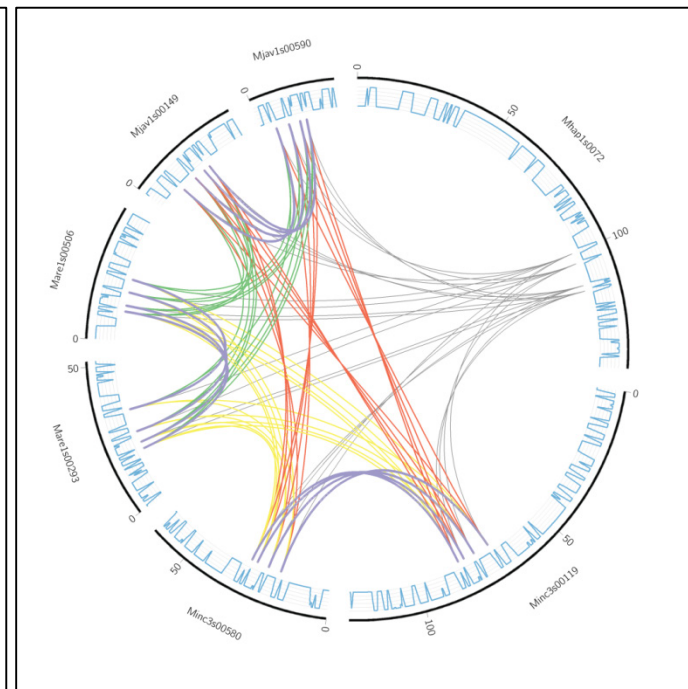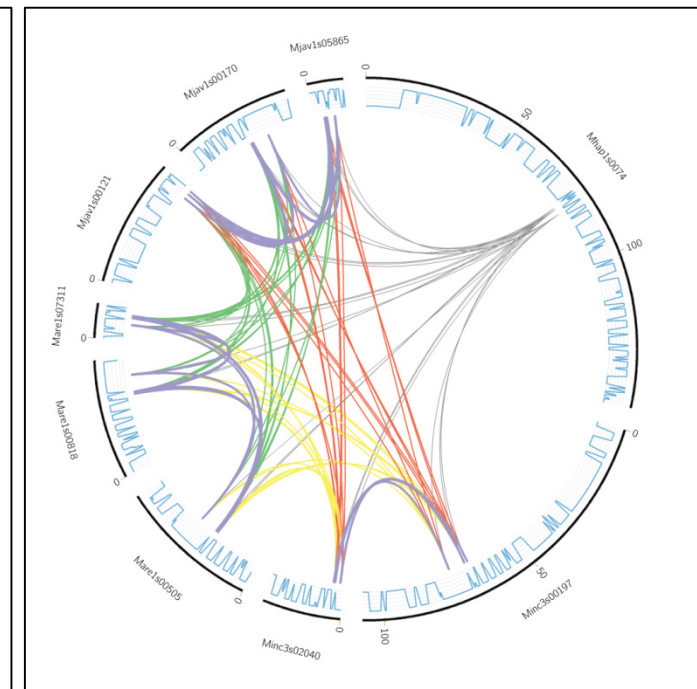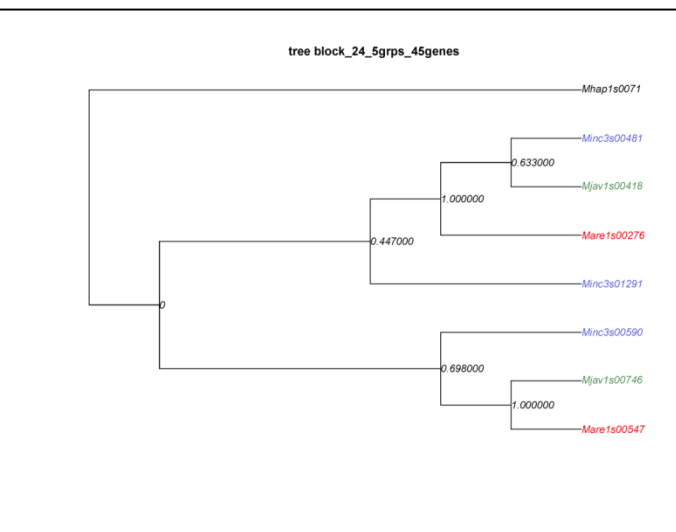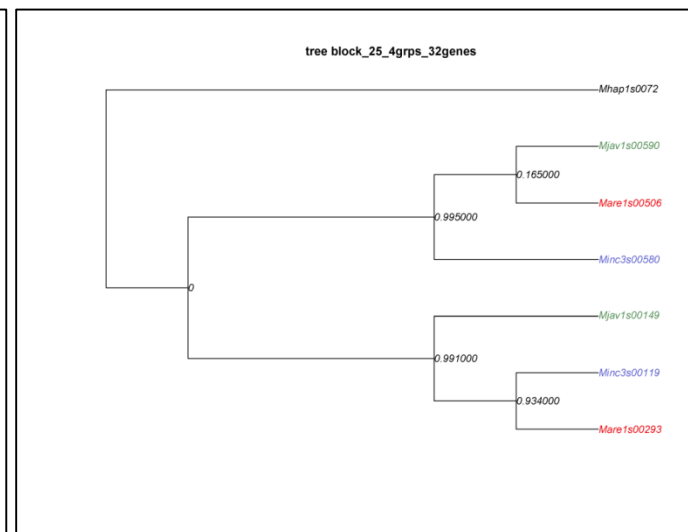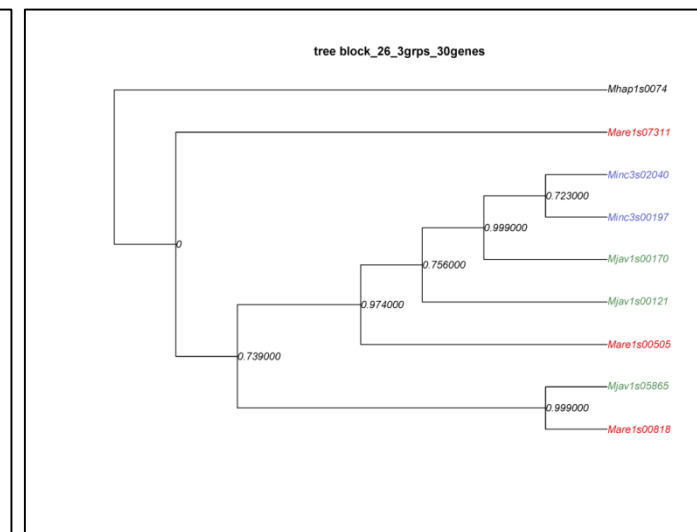

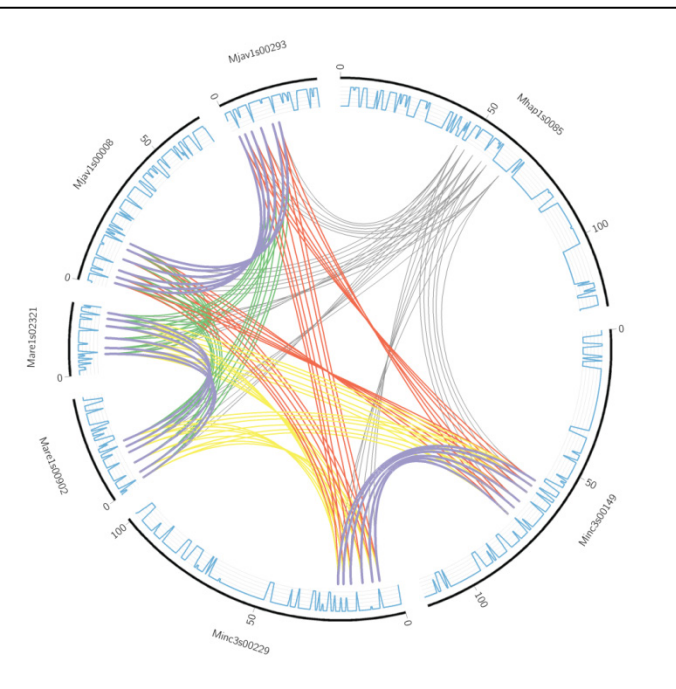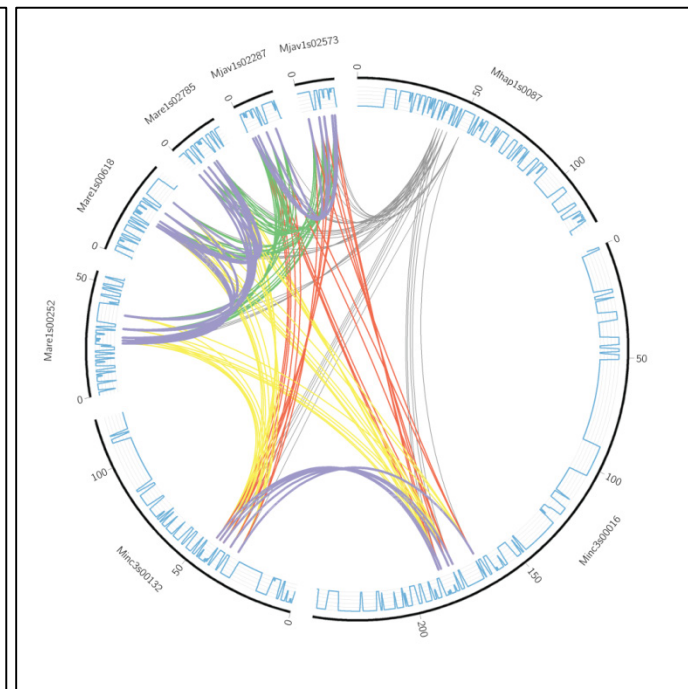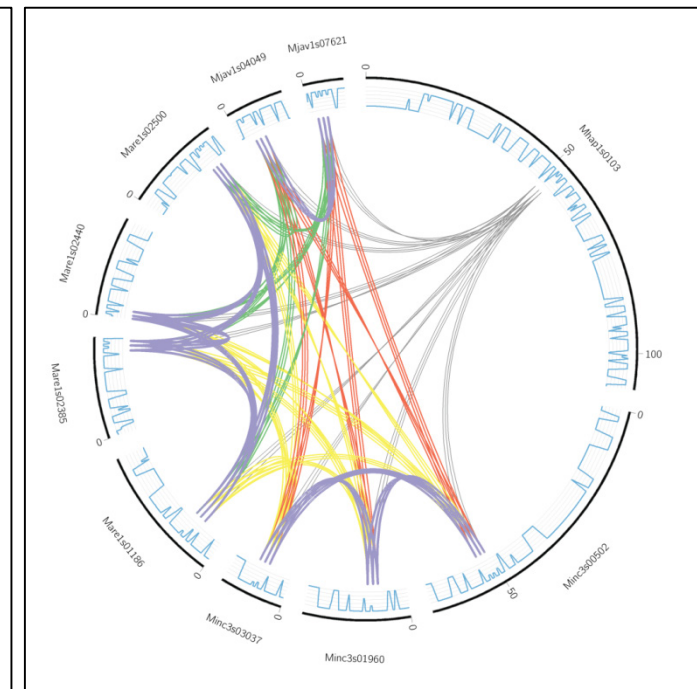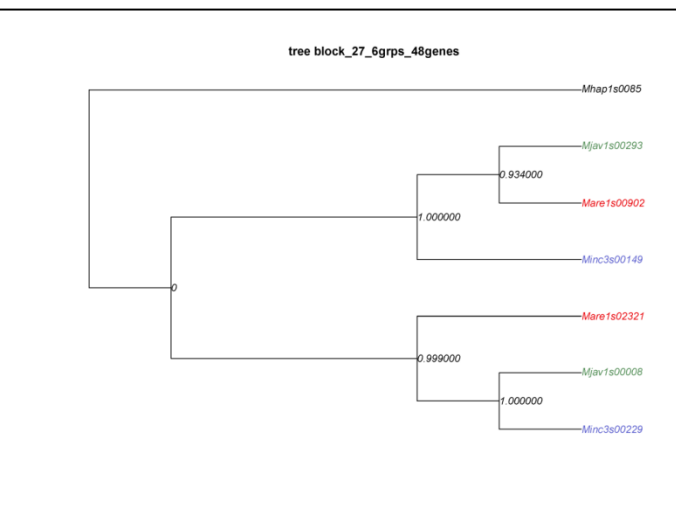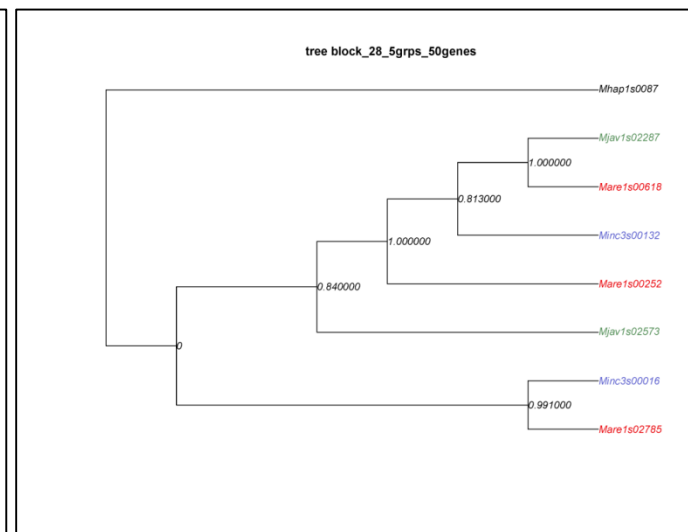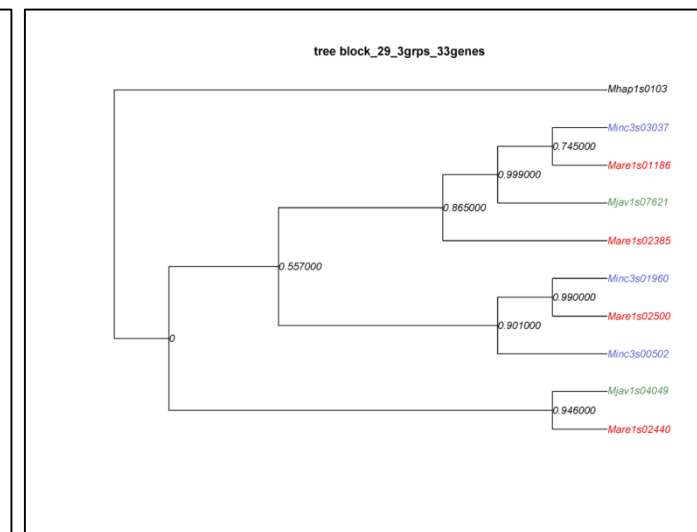

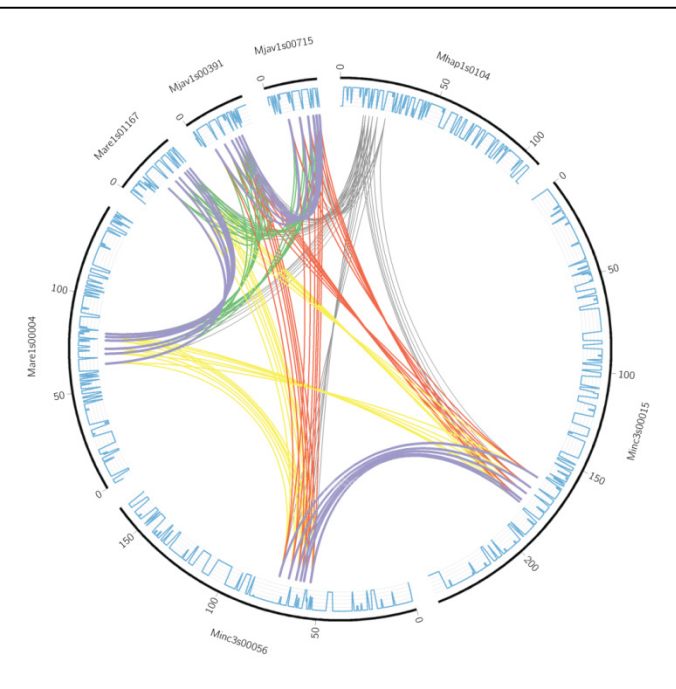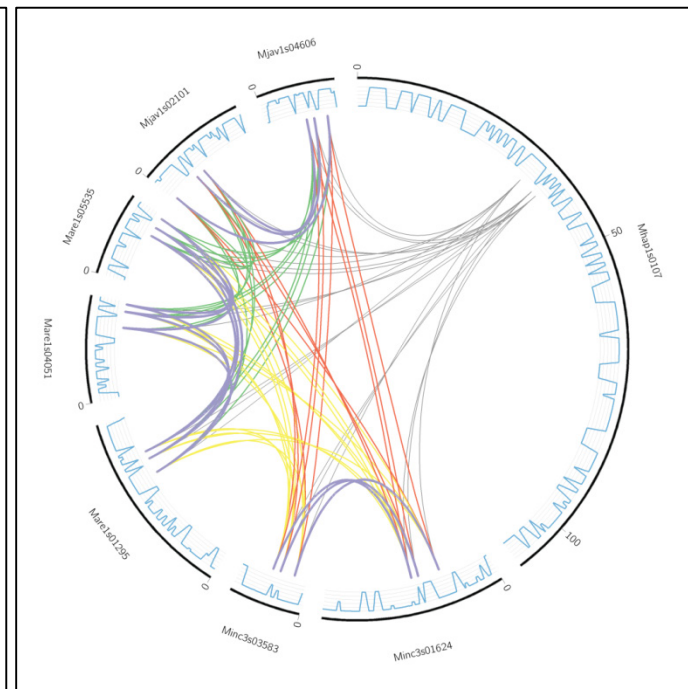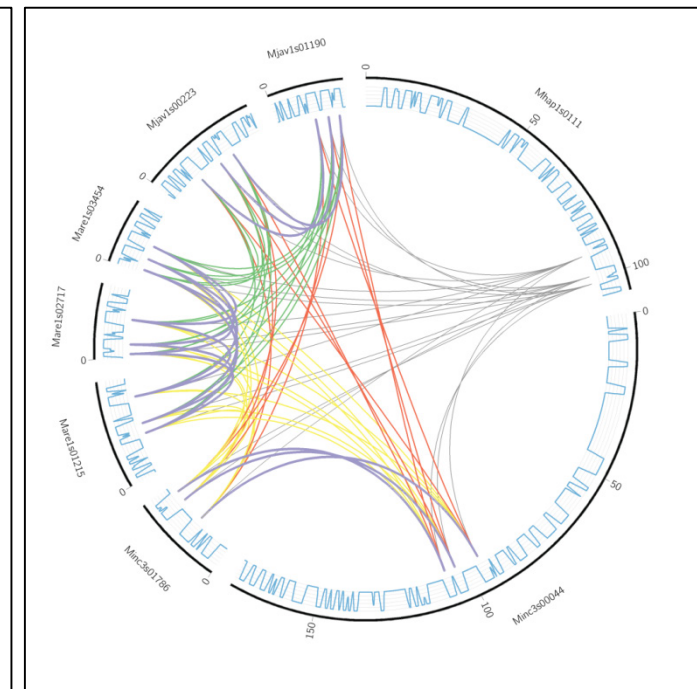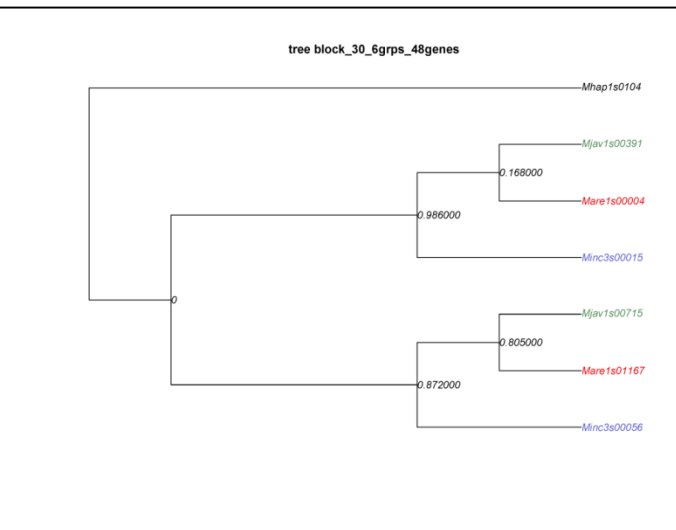

No tree

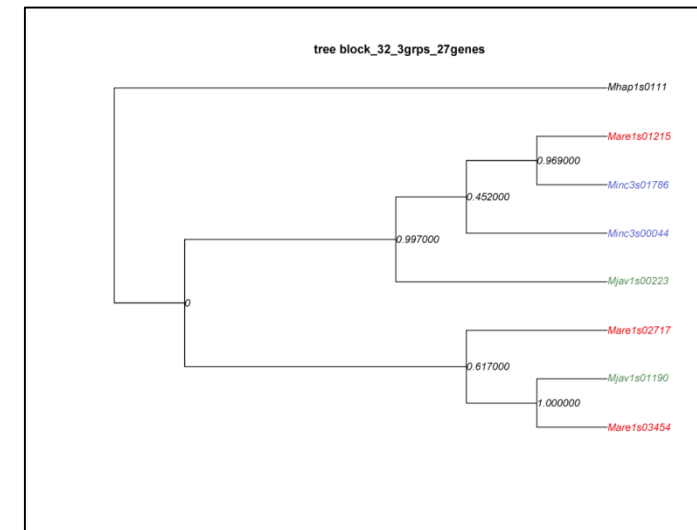



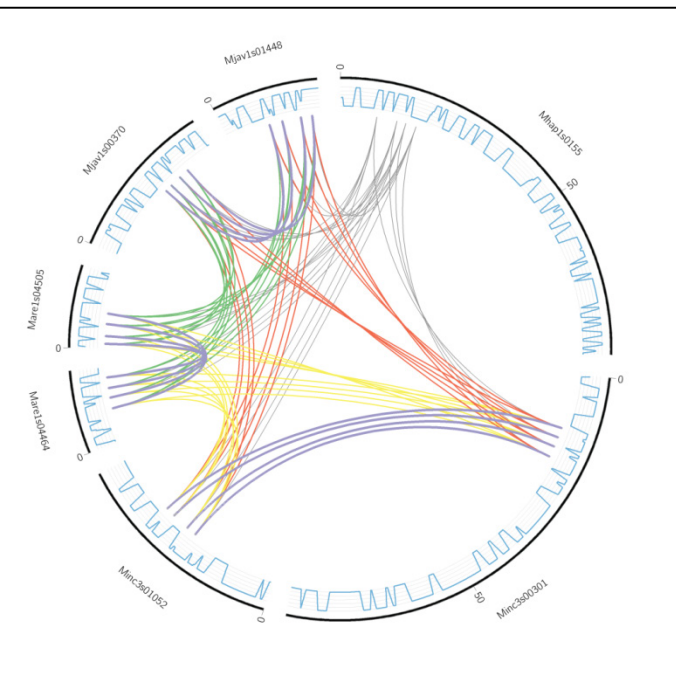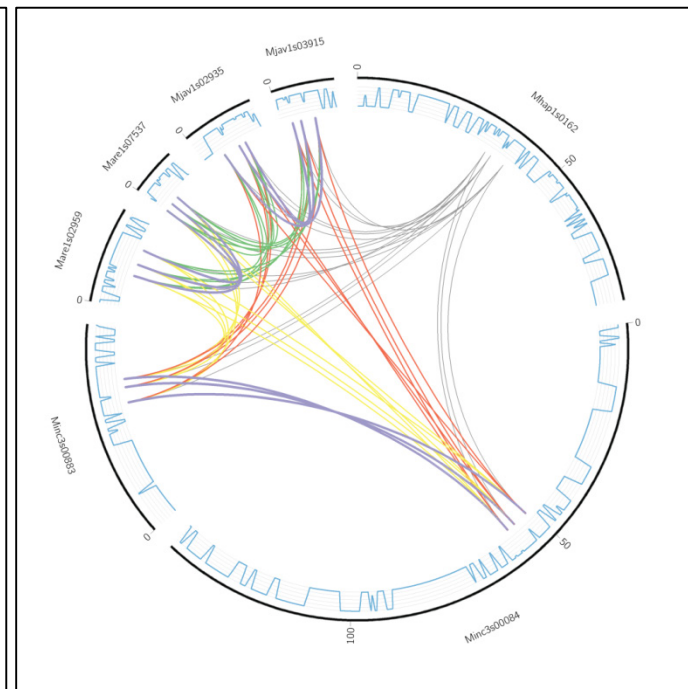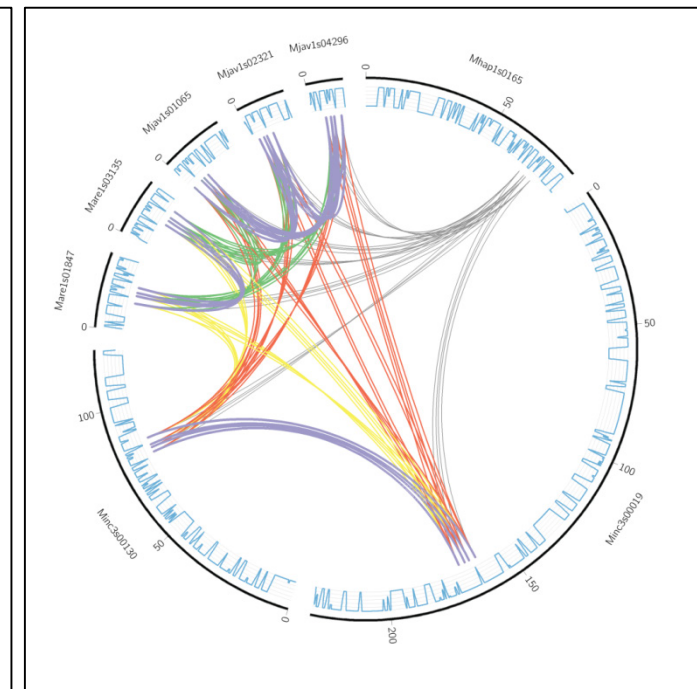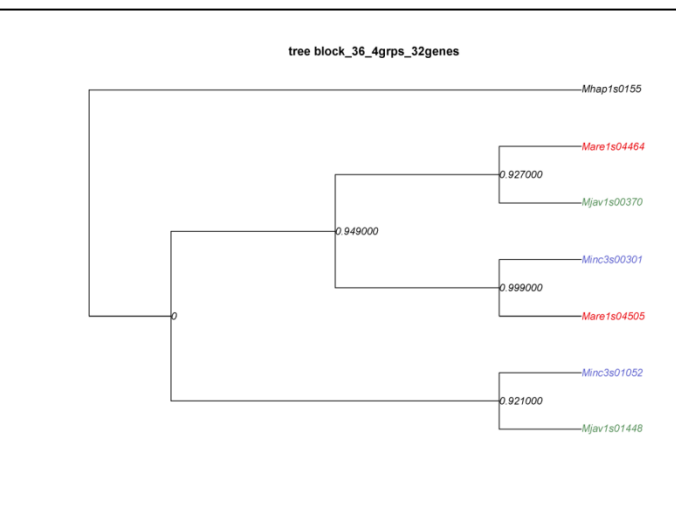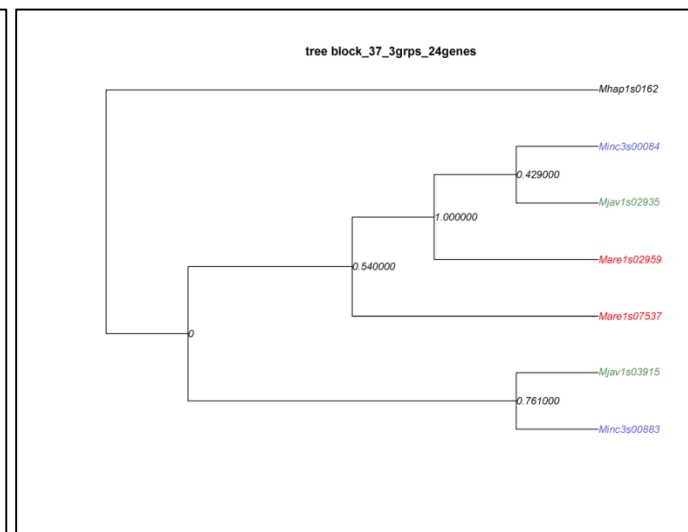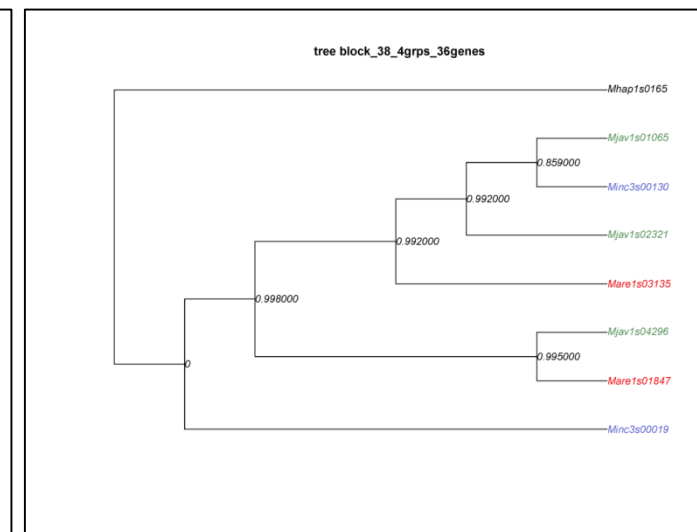

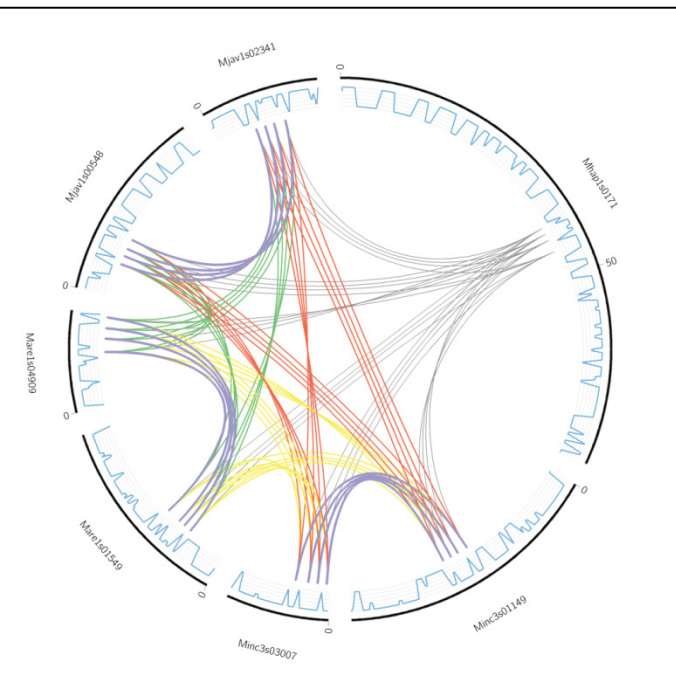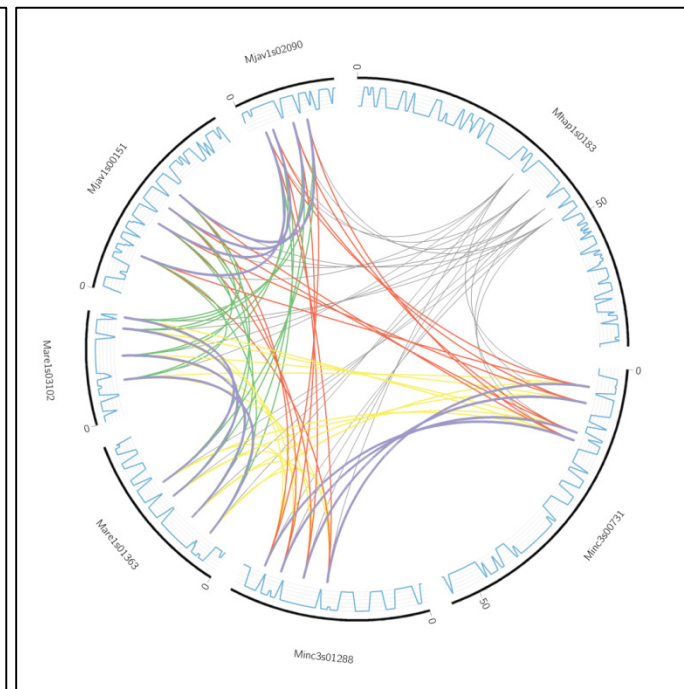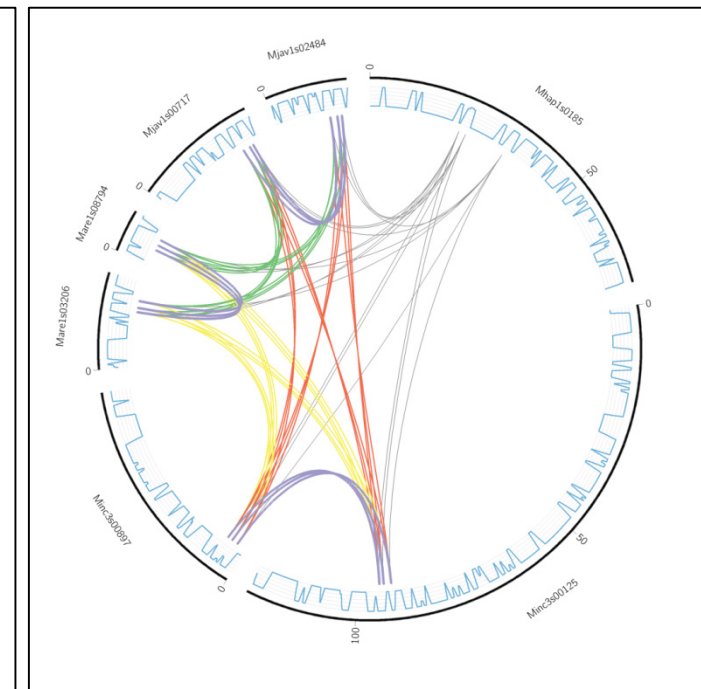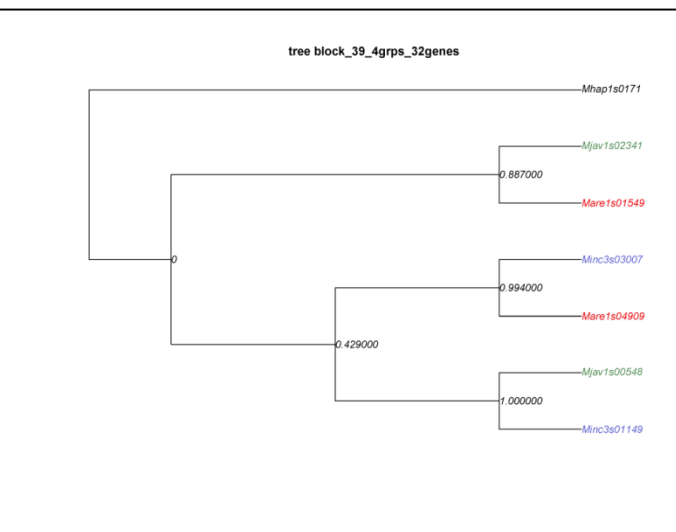

No tree

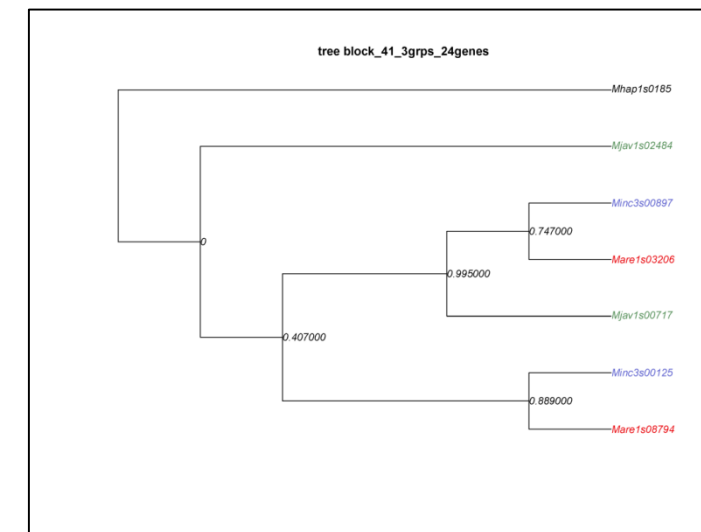

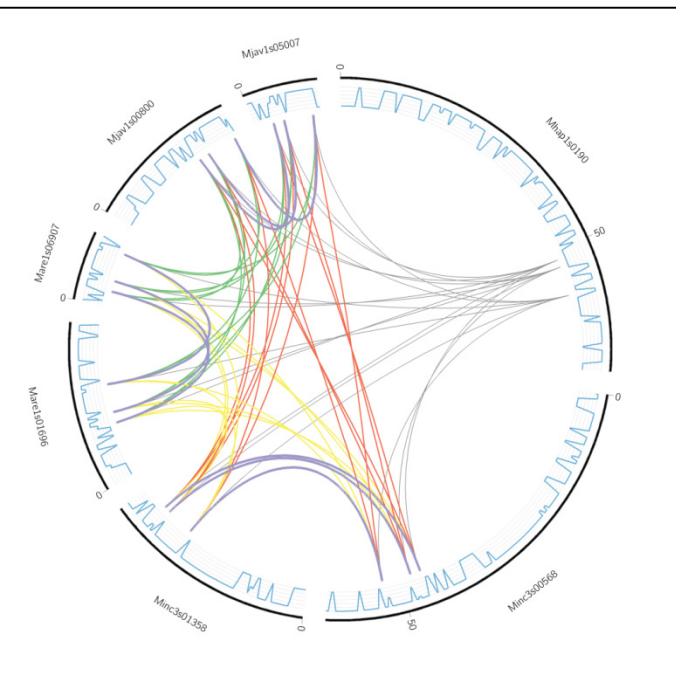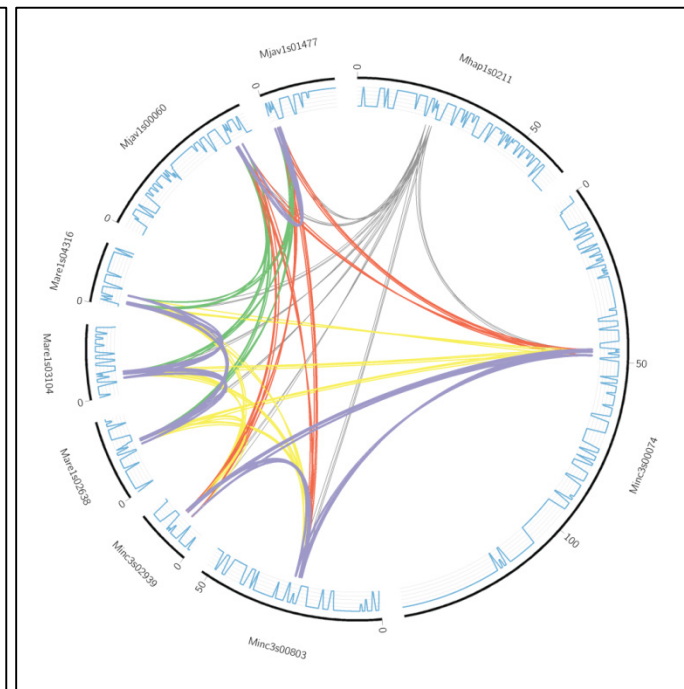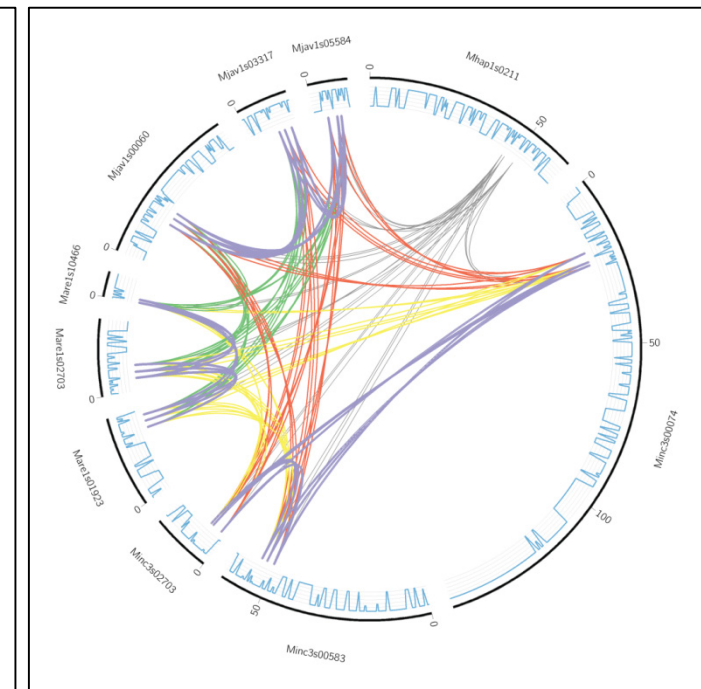

No tree

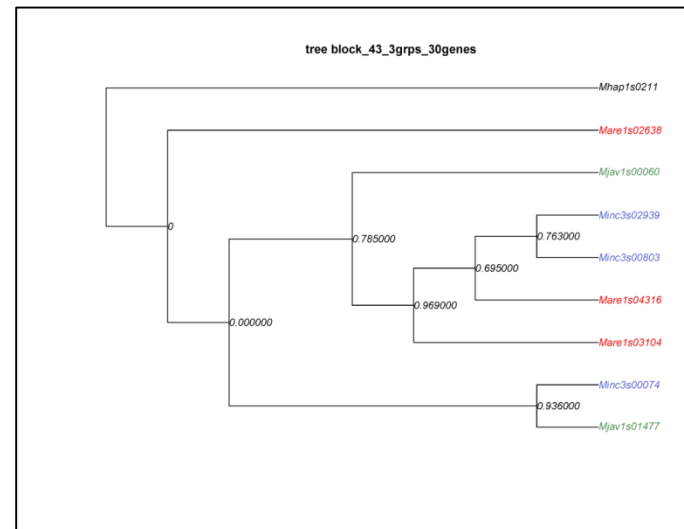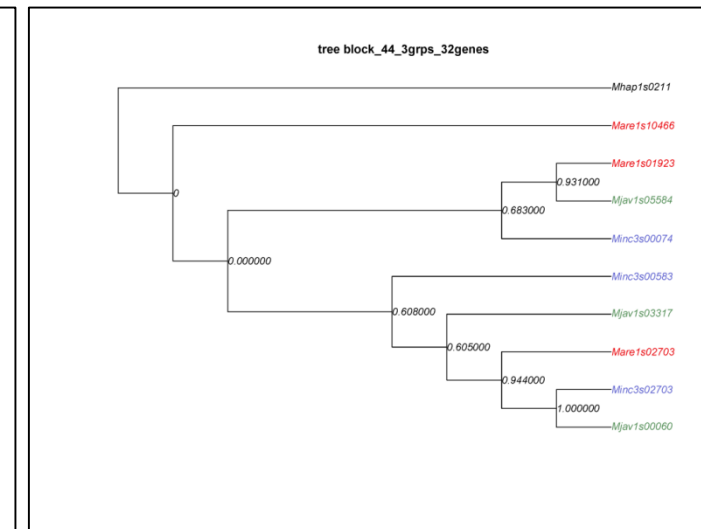

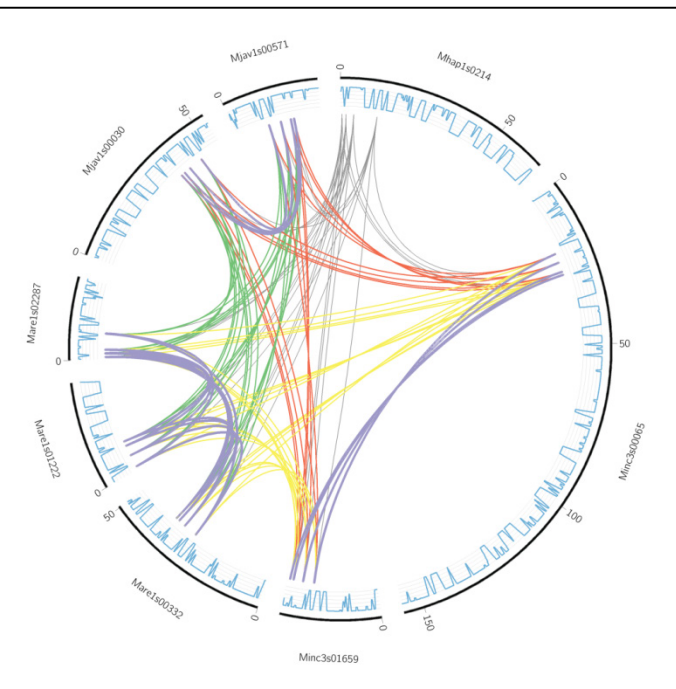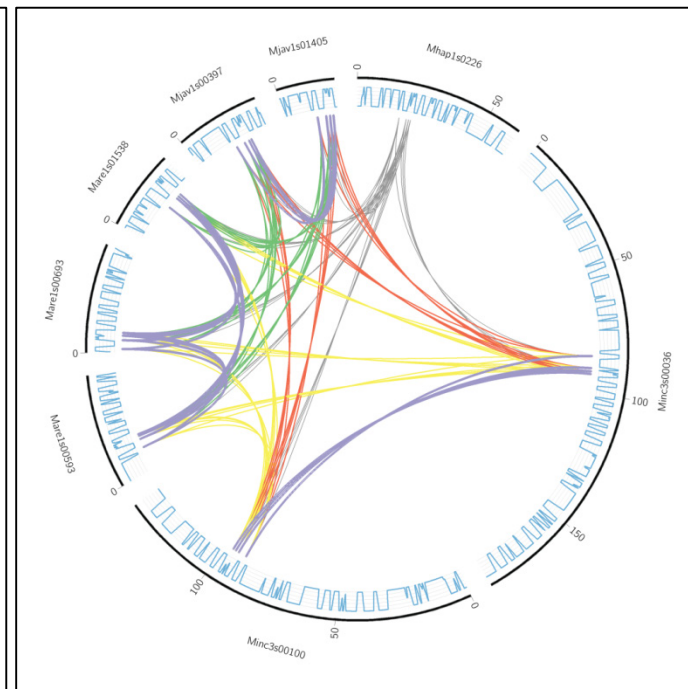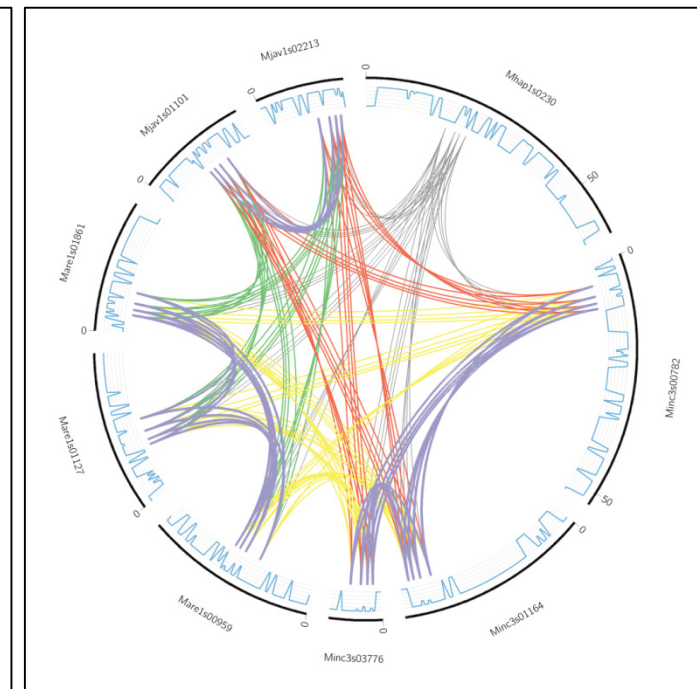

No tree

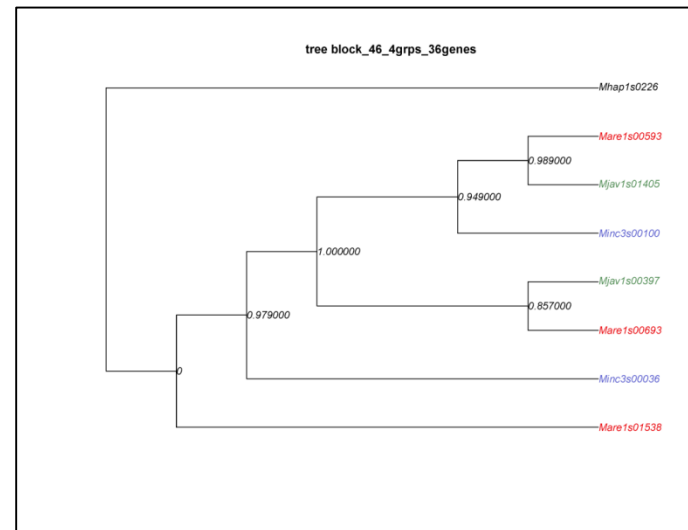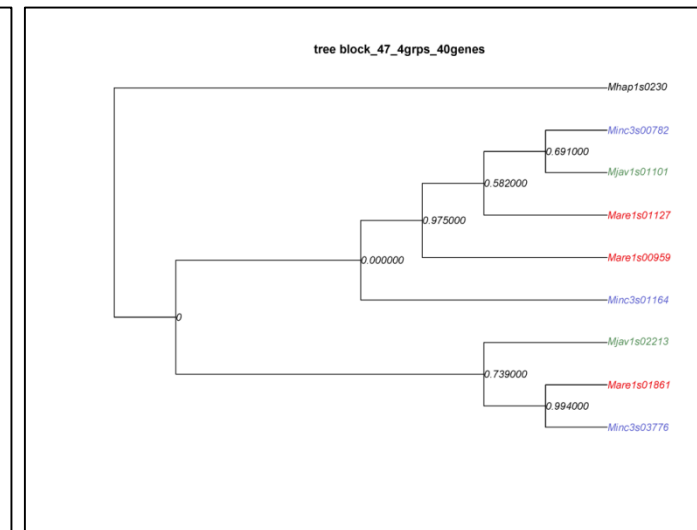

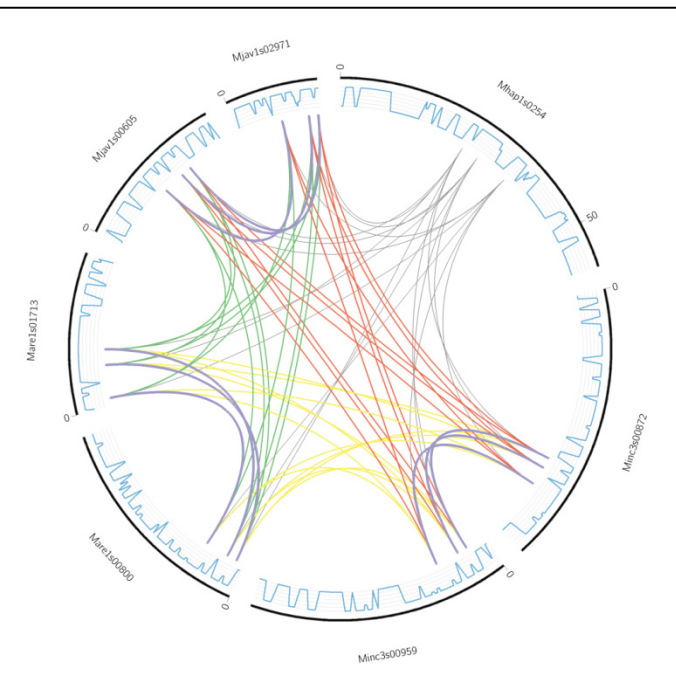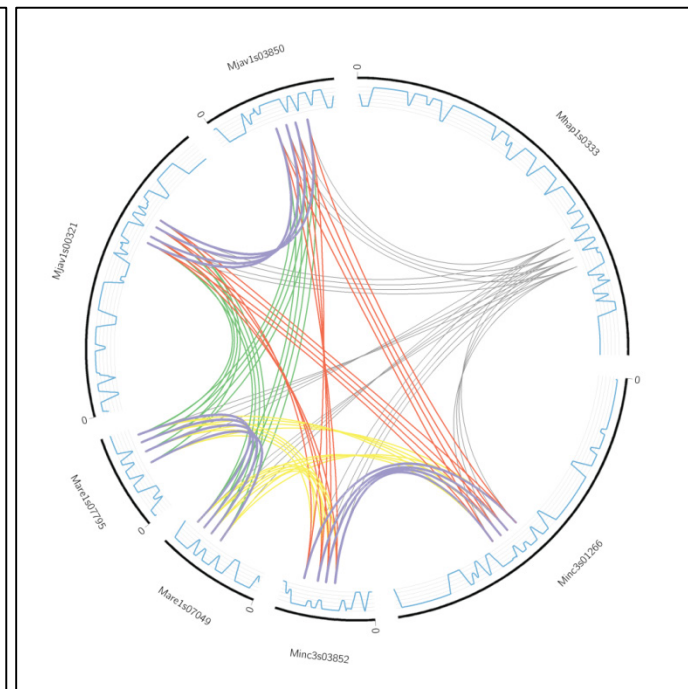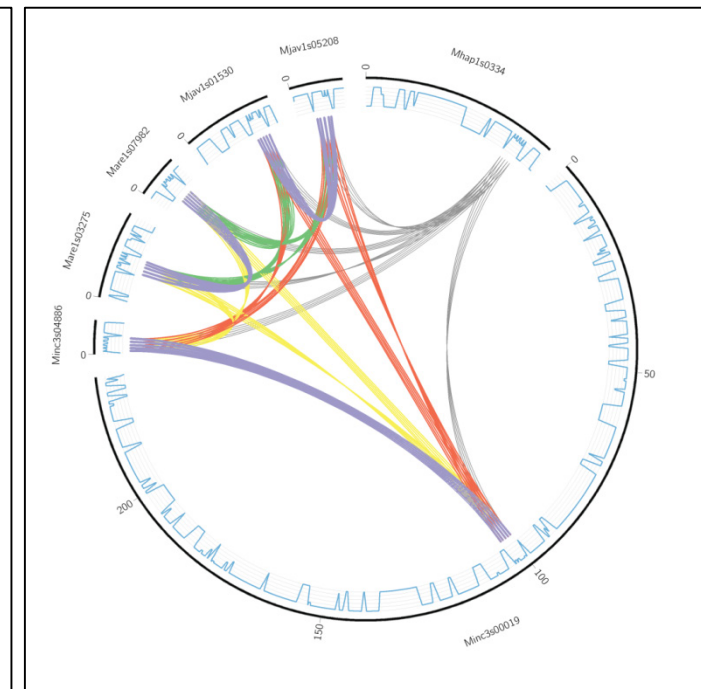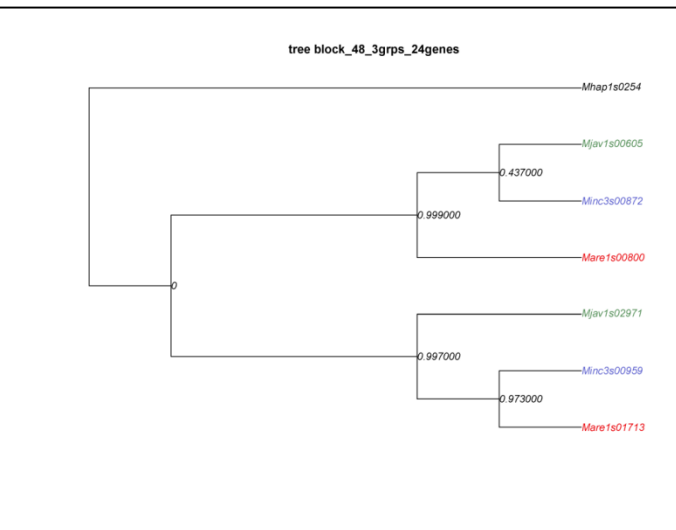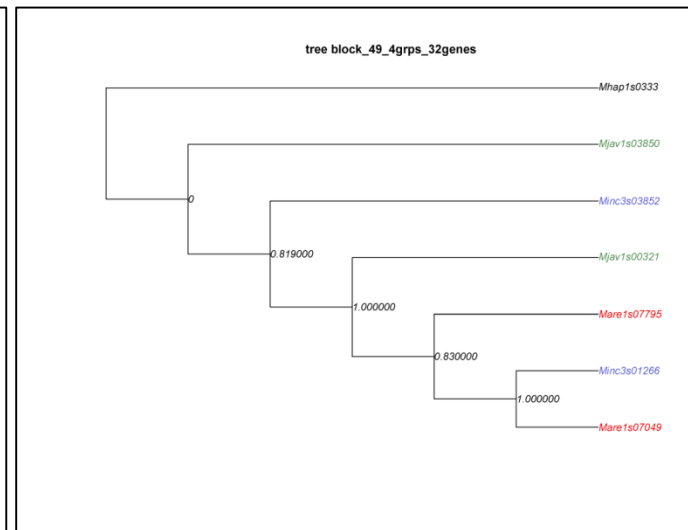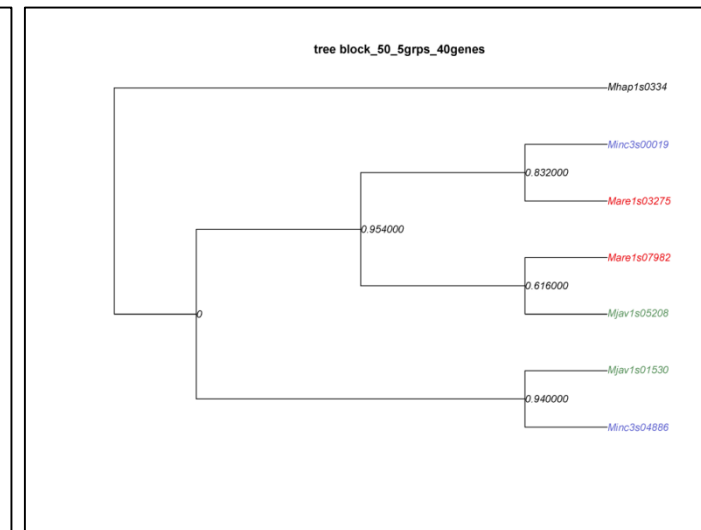

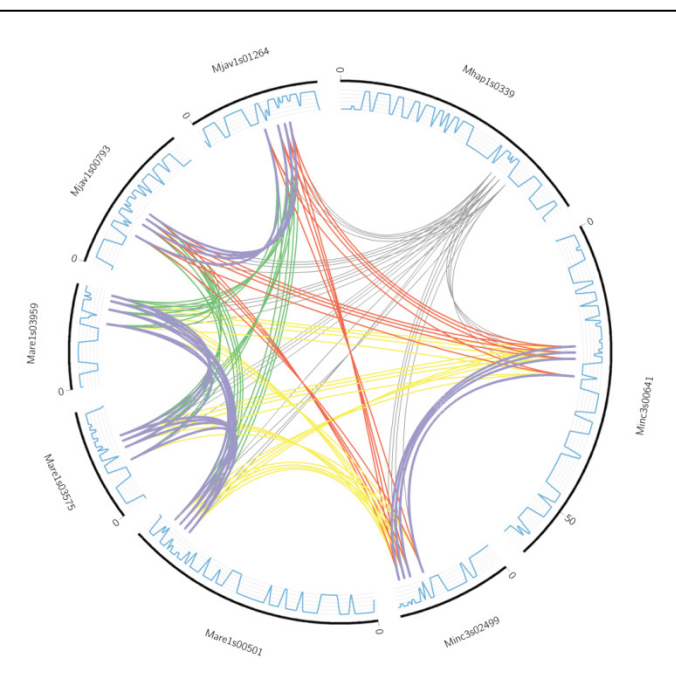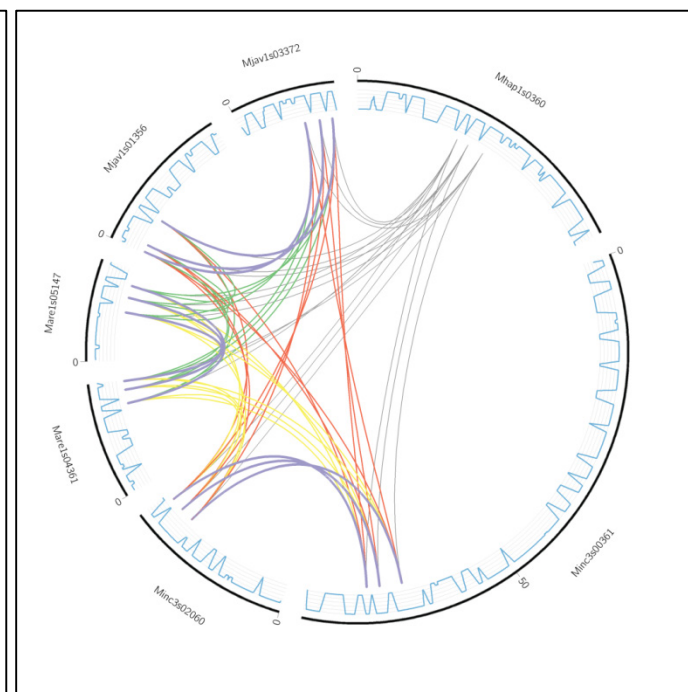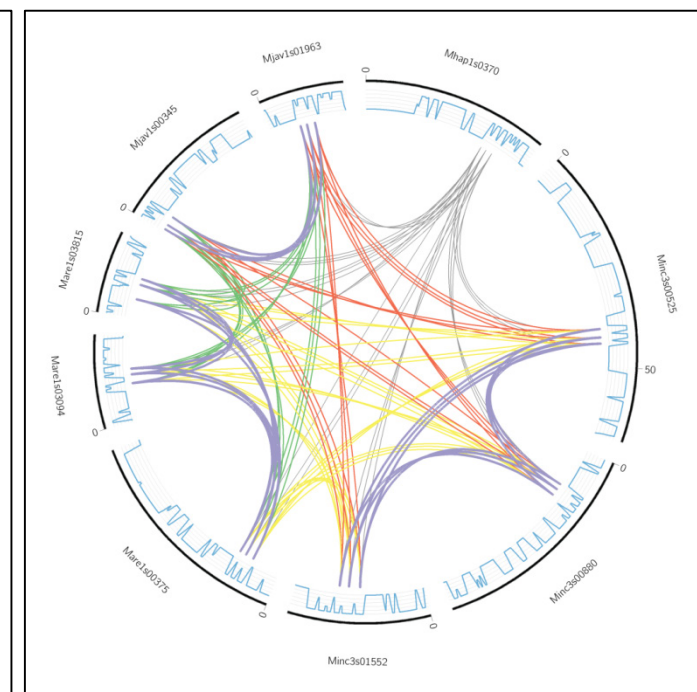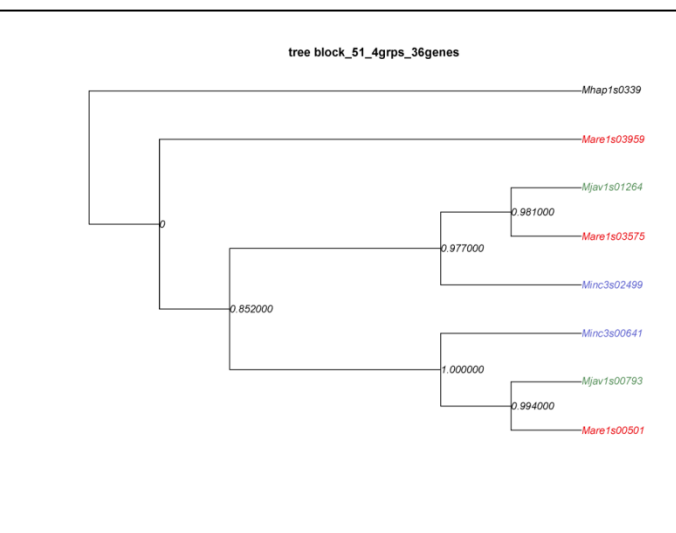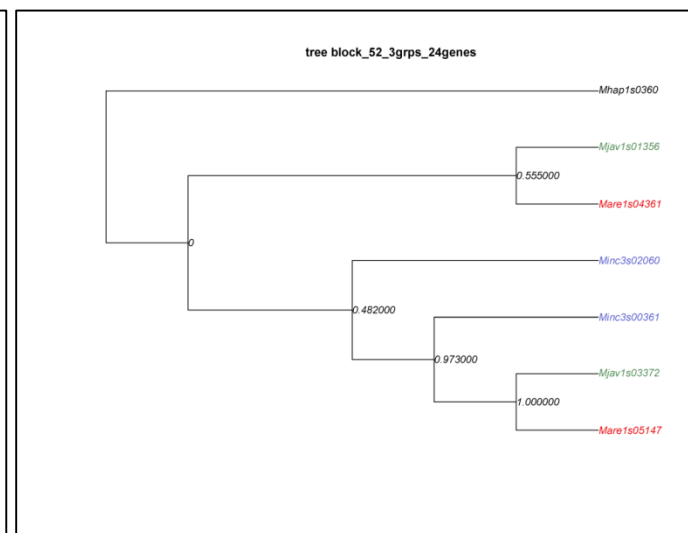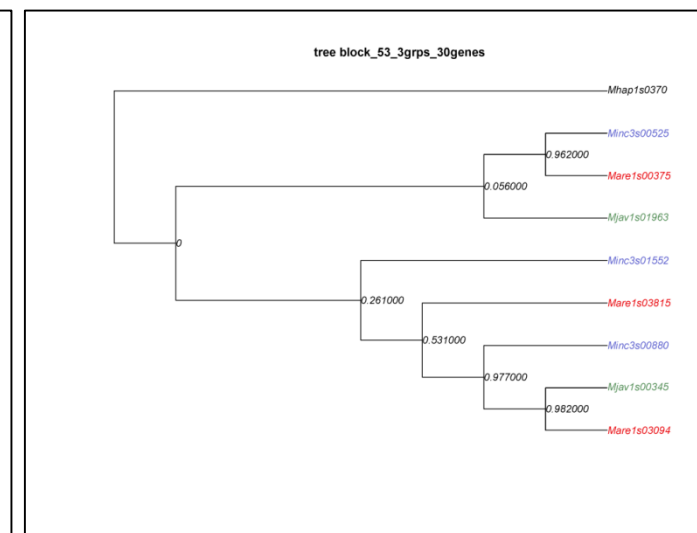

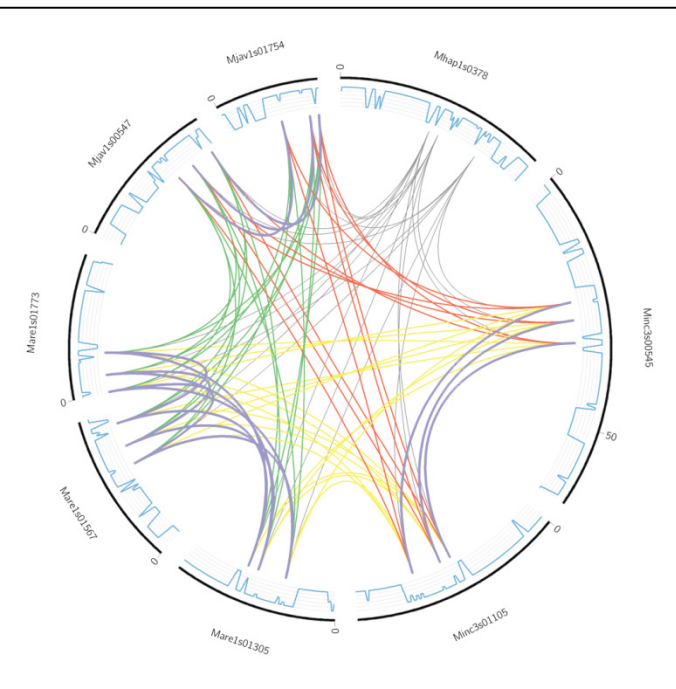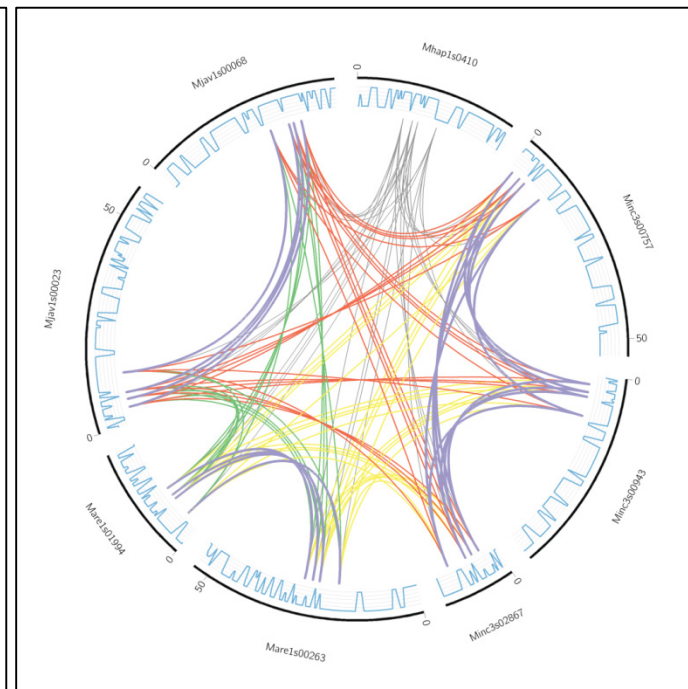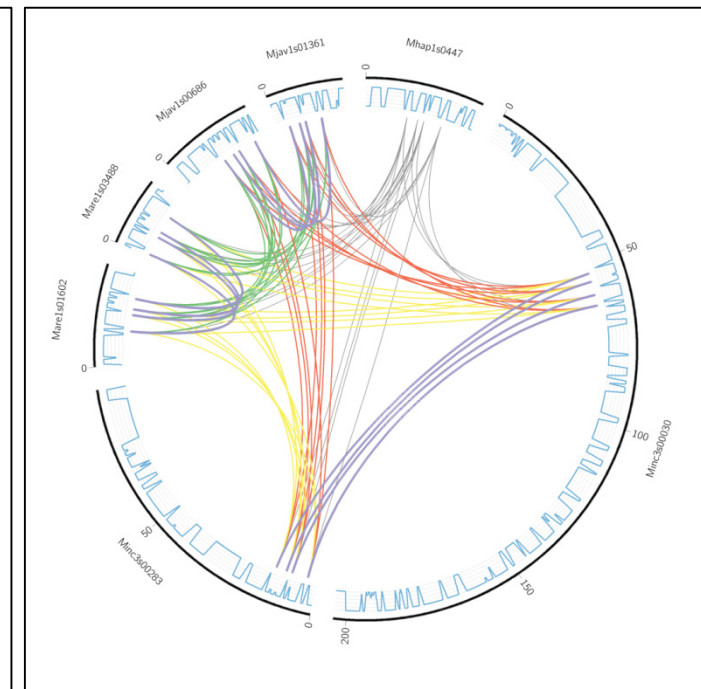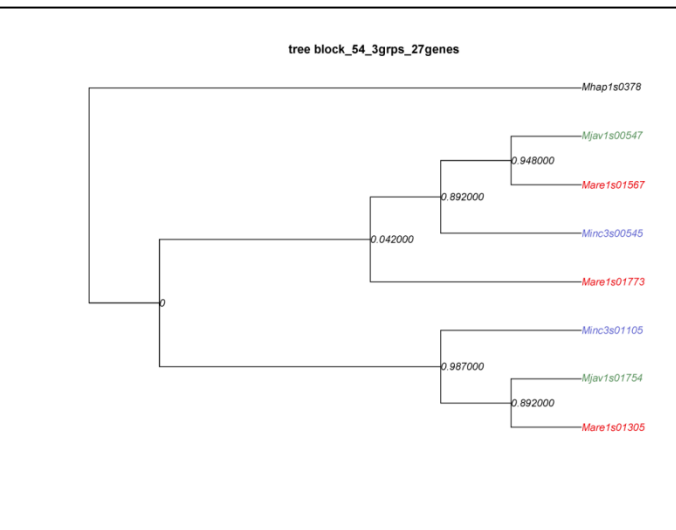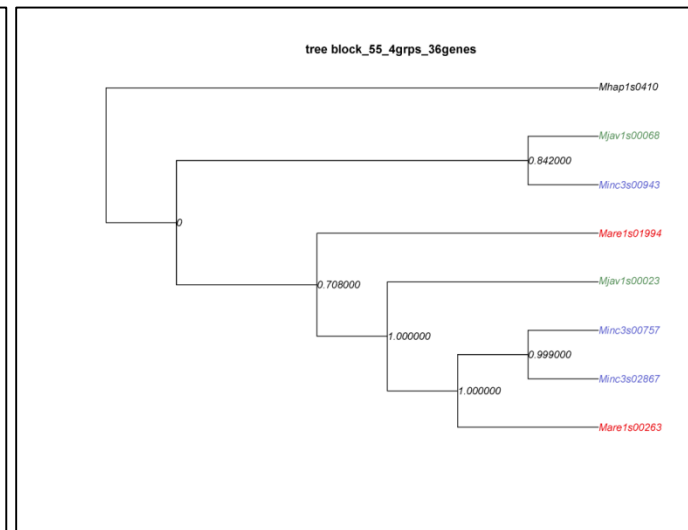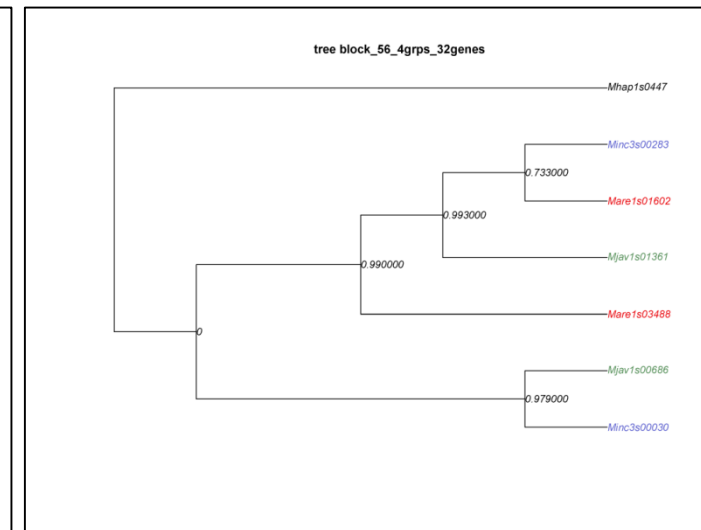

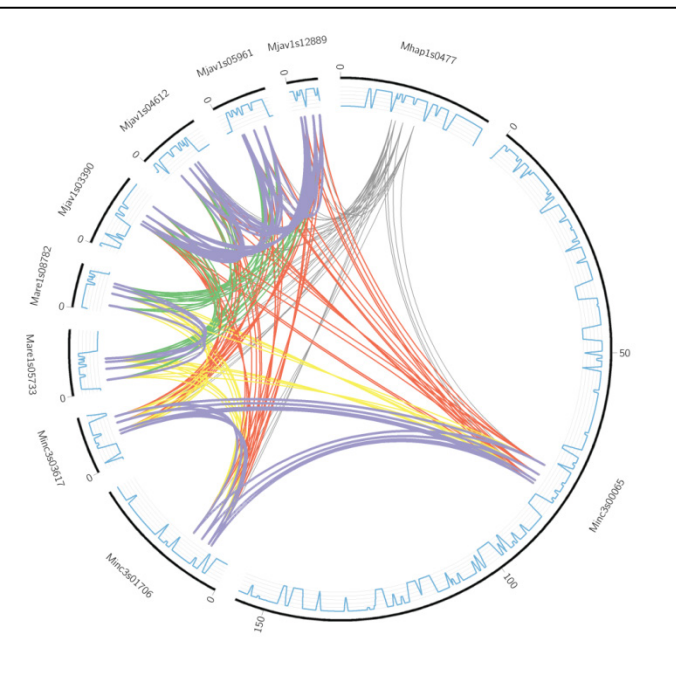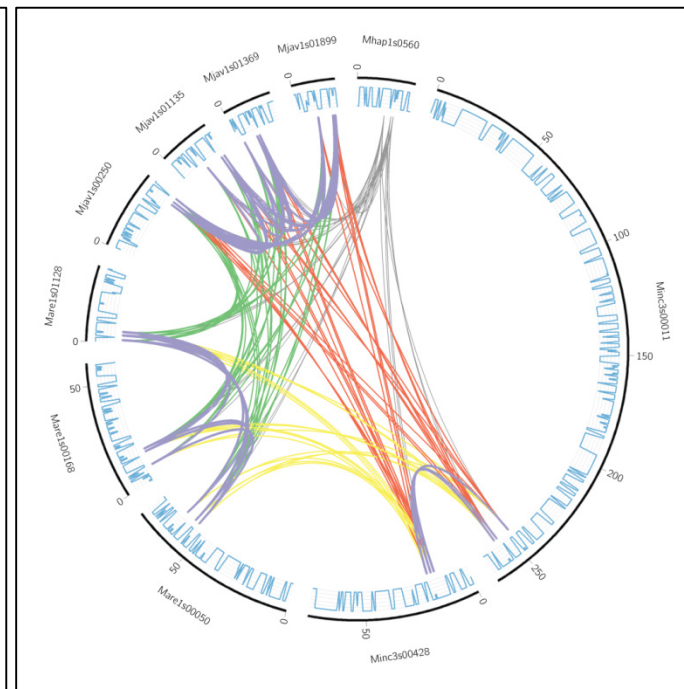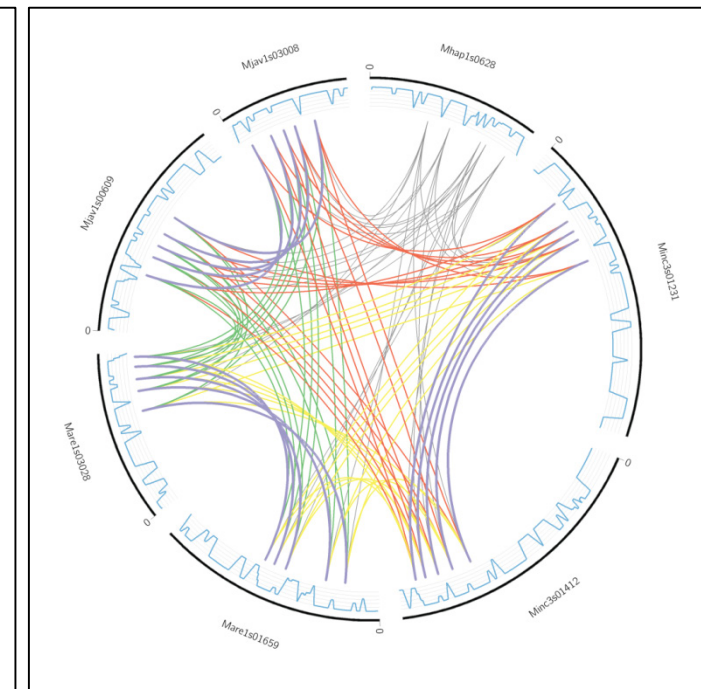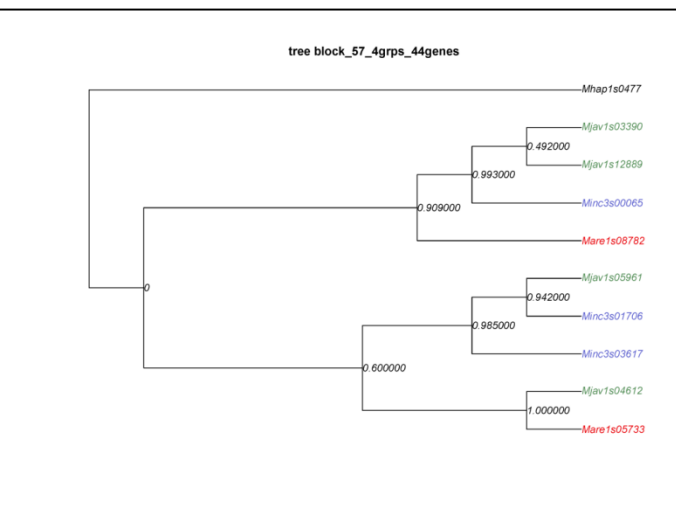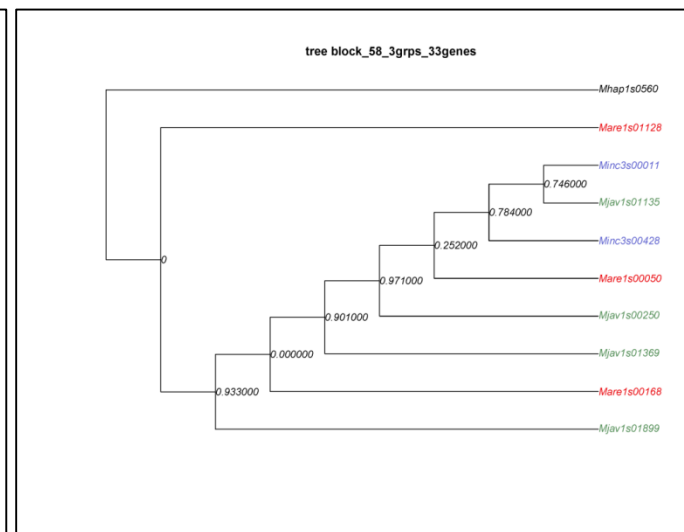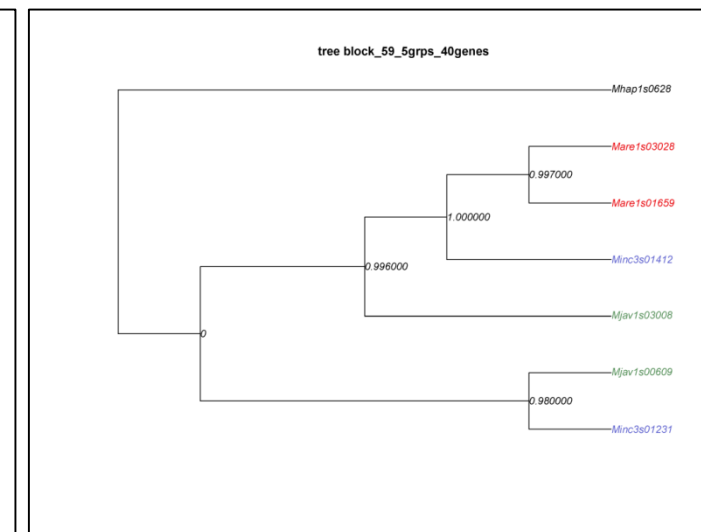

Supplement: S5 Fig — In each Circo (unit = kb), color codes between collinear blocks are as follows. Collinear orthologs between M. hapla and any of the three asexuals species in grey. Collinear ‘homoeologs’ within asexual species in purple. Collinear orthologs between M. arenaria and M. javanica in green. Collinear orthologs between M. arenaria and M. incognita in yellow. Collinear orthologs between M. incognita and M. javanica in red. The outer scaled blue line represents gene density on scaffolds. The corresponding ML phylogenies performed on the concatenated alignments per blocks are given below each Circo. (PDF) [file pgen.1006777.s005.pdf]

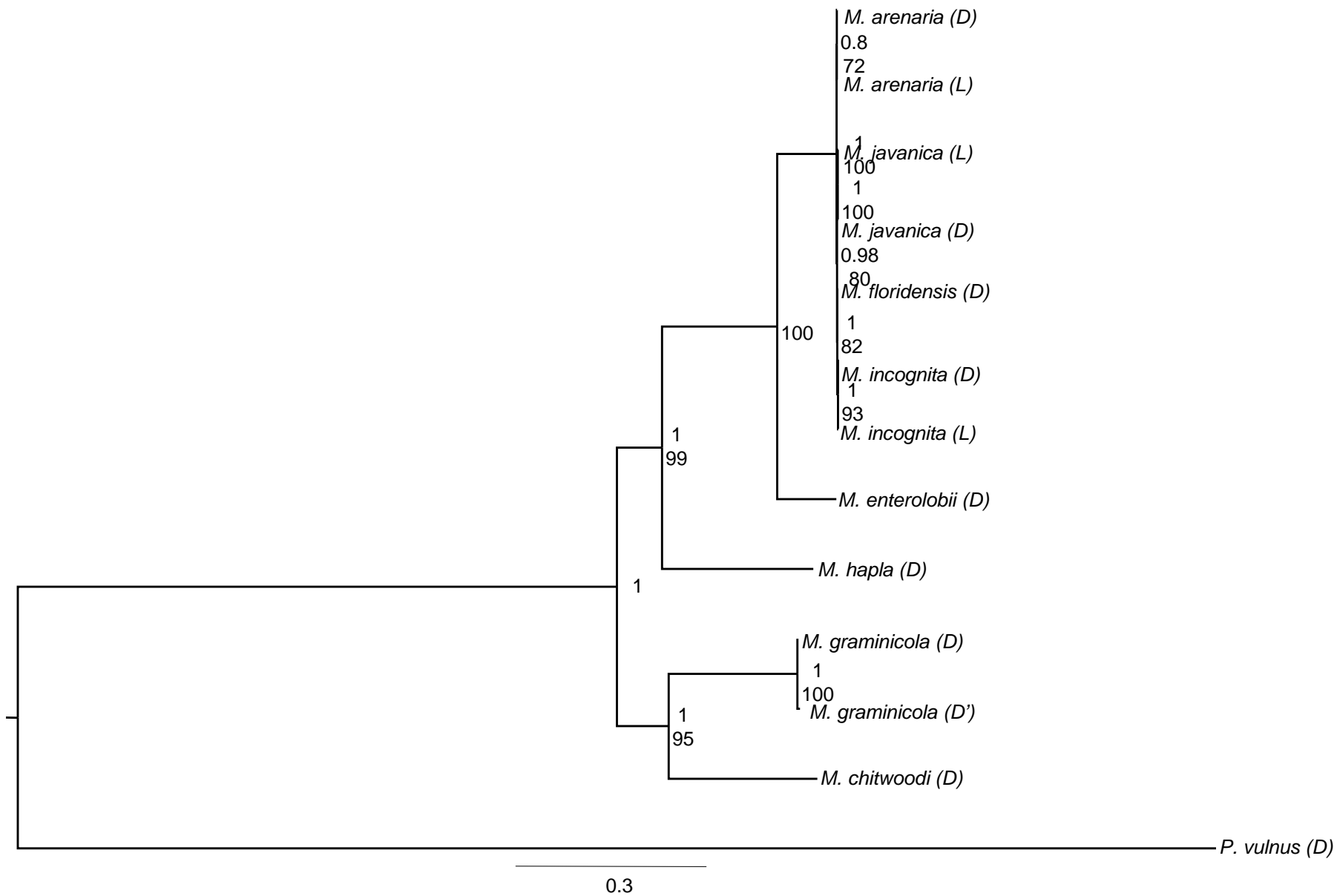

Supplement: S6 Fig — Mitochondrial phylogenetic tree of Meloidogyne with actual branch lengths showing high identity level between apomictic root-knot nematodes. (PDF) [file pgen.1006777.s006.pdf]

$\omega$  rate class

0.10

0.02

0.01

0

0.998

0.873

0.107

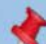

Minc3s00119g05130

Mjav1s00025g00060

Minc3s00043g02389

Mjav1s00402g005840

Mare1s02197g031032

Mhap1s0072g04803

Supplement: S7 Fig — Example of a duplicated gene copy harboring signature of episodic diversifying selection as detected by the Branch-Site REL model. The branch leading to the gene Mjav1s00402g005840 is inferred to be under (or to have underwent) episodic diversifying selection (corrected p = 0.013). The hue of each color indicates strength of selection, with primary red corresponding to ω > 5, primary blue to ω = 0 and grey to ω = 1. The width of each color component represents the proportion of sites in the corresponding class. Thicker branches have been classified as undergoing episodic diversifying selection by the sequential likelihood ratio test at corrected p ≤ 0.05. ω: the inferred rates of non-synonymous mutations / rate of synonymous mutations ratio. (PDF) [file pgen.1006777.s007.pdf]

**A**

Nuclei number

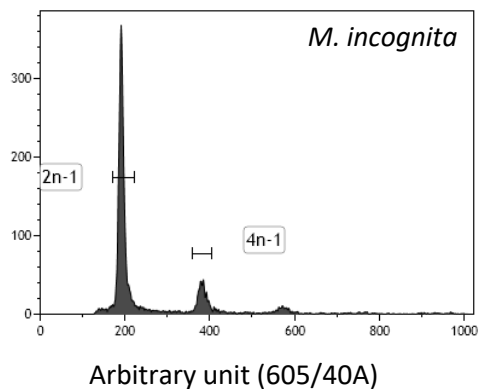**B**

Nuclei number

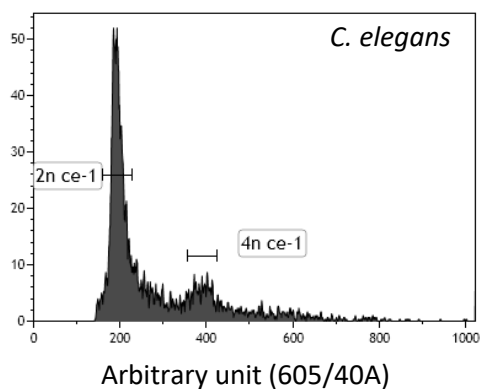**C**

Nuclei number

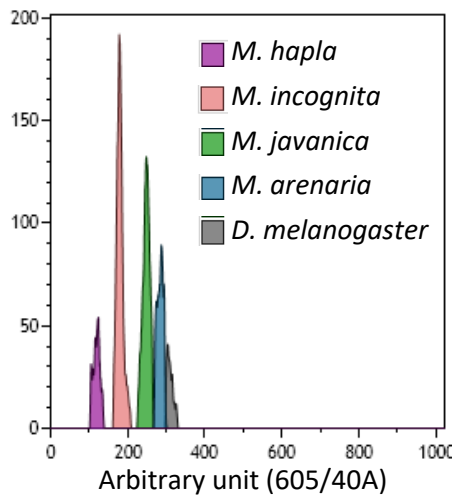

Supplement: S8 Fig — Example of an unfiltered cytogram (arbitrary units) where nuclei were processed alone in a single acquisition: (A) M. incognita sample and (B) C. elegans standard. (C) Cytogram example obtained after gating on G0/G1 nuclei (arbitrary units) from each Meloidogyne species (M. hapla, M. incognita, M. javanica and M. arenaria) when processed mixed altogether with an internal standard (D. melanogaster: approximately 350 Mb). (PDF) [file pgen.1006777.s008.pdf]
