# Supplementary material for: Hybridization and polyploidy enable genomic plasticity without sex in the most devastating plant-parasitic nematodes
Source: PLoS Genet. 2017 Jun 8;13(6):e1006777. doi: 10.1371/journal.pgen.1006777 (PMC5465968; doi:10.1371/journal.pgen.1006777)

sequence2 Minc3s00990:3627-27194

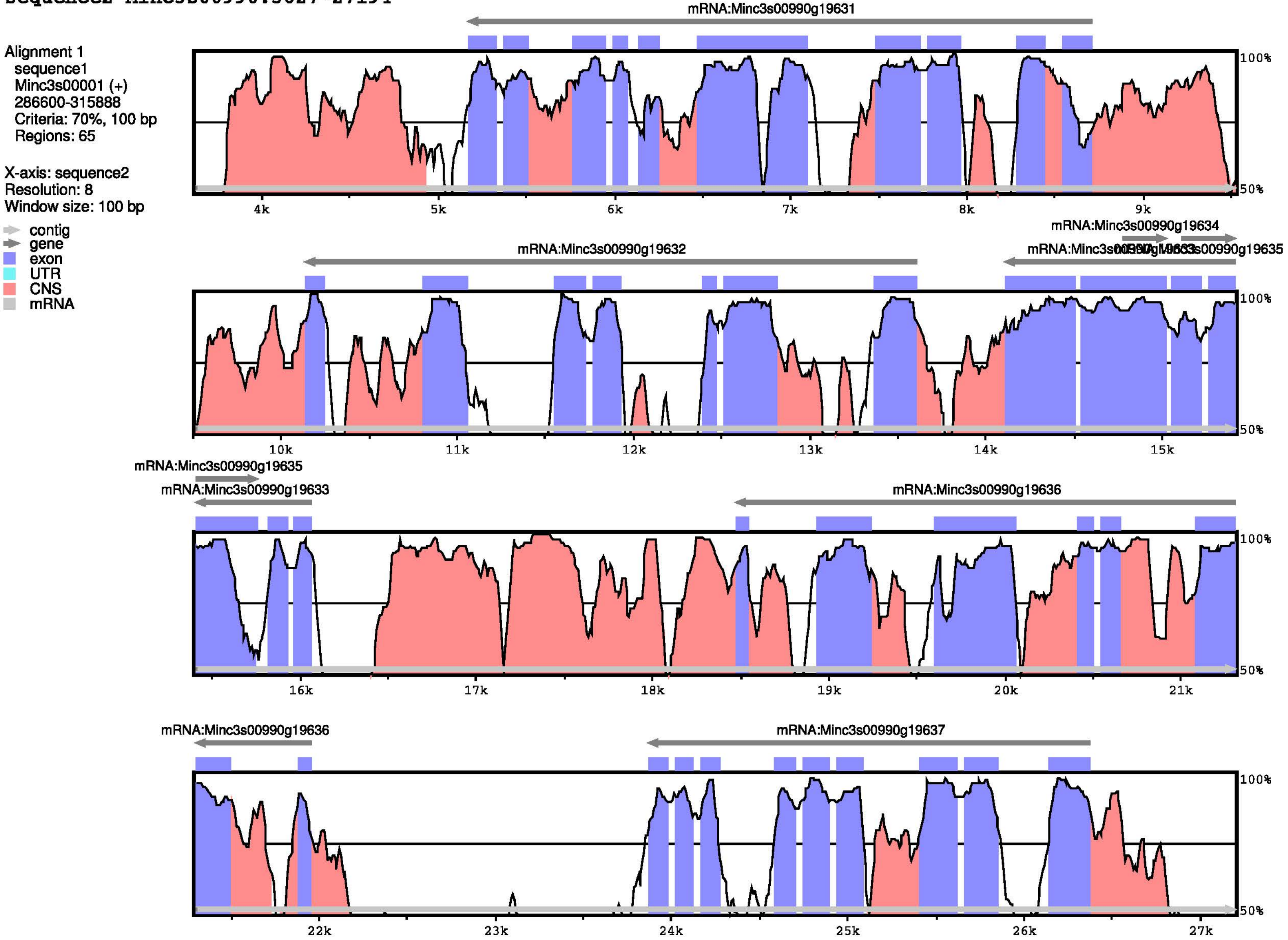

Supplement: S3 Fig — To illustrate the divergence between pair of duplicated collinear blocks formed by MCScanX, we aligned (using LAGAN: at http://genome.lbl.gov/vista/index.shtml), 2 collinear blocks of M. incognita. Block located on scaffold “Minc3s00001” from position 286,600 to 315,888 is aligned against its homoeolog block located on scaffold “Minc3s00990” from position 3,627 to 27,194. Percentage identity (between 50 and 100%) is plotted against the scaffold sequence (light grey line) for local alignment with 70% coverage and a minimum of 100 bp. Location of homologous genes is indicated by dark grey arrows. Identity is in salmon for conserved non-coding sequences (CNS) and in light slate blue for protein-coding sequences. (PDF) [file pgen.1006777.s003.pdf]
