# Supplementary material for: Hybridization and polyploidy enable genomic plasticity without sex in the most devastating plant-parasitic nematodes
Source: PLoS Genet. 2017 Jun 8;13(6):e1006777. doi: 10.1371/journal.pgen.1006777 (PMC5465968; doi:10.1371/journal.pgen.1006777)
Supplement: S1 Text — (PDF) [file pgen.1006777.s009.pdf]

SUPPLEMENTARY METHODS, RESULTS, NOTE AND DISCUSSION

Table of contents

Methods for the mitochondrial phylogeny 1

Supplementary results on Pfam and GO annotation........ 2

Supplementary discussion: origin of the duplicated genome structure of apomictic Meloidogyne........ 4

The Meselson-White effect hypothesis can be rejected 4

Hybridization appears as the most likely origin........ 5

Single vs. multiple founding hybridizations........ 7

Fractionation bias 7

Ploidy levels 7

Supplementary methods: biological material, DNA and RNA sequencing 8

Meloidogyne strains 8

DNA sequencing 8

RNA sequencing........ 9

NOTE S1: DATA AVAILABILITY........ 9

REFERENCES........ 10

Methods for the mitochondrial phylogeny

Canonical nematode mitochondrial genomes encode 12 proteins (ATP6, CO1, CO2, CO3, COB, ND1, ND2, ND3, ND4, ND4L, ND5 and ND6), 2 rRNAs (12S and 16S) as well as 22 tRNAs. The sequences of the 12 protein-coding genes and the 2 mitochondrial rRNA genes were retrieved from the 8 *Meloidogyne* species for which mitochondrial information was available in the literature (S1 Table): *M. incognita* and *M. chitwoodi* [1], *M. javanica*, *M. arenaria* and *M. enterolobii* [2], *M. floridensis* [3], *M. hapla* [4] and *M. graminicola* [5,6], as well as from *Pratylenchus vulnus* [7], the closest outgroup with a sequenced mitochondrial genome. Because mitochondrial genomes were also sequenced as part of our sequencing effort of the nuclear genomes of *M. incognita*, *M. javanica* and *M. arenaria*, the 12 protein-coding and 2 rRNA mitochondrial genes were also searched and annotated in the corresponding contigs in genome assemblies. Searching in our lab-produced *Meloidogyne* sequences allowed us to make sure the exact same strain was compared both at the nuclear and mitochondrial levels for the 3 apomictic species. Each of the 12 protein-coding genes was aligned at the protein level using MAFFT [8] in automatic mode and then reverse-translated into nucleotide sequences using PAL2NAL [9]. The 2 rRNA genes were directly aligned at the nucleotide level using MAFFT [8] in automatic mode. The 14 multiple

alignments (12 protein-coding and 2 rRNAs) were concatenated in a supermatrix using an in-house python script. Alignment columns containing more than 40% of gaps were eliminated using trimAL [10]. We used JModelTest [11] to determine the best evolutionary model for each gene. Phylogenetic analyses were performed on the supermatrix using a Bayesian approach in MrBayes 3.2.4 [12] and a maximum-likelihood approach (ML) in RAxML 8.1.5 [13]. In both cases, the fittest evolutionary models were specified for each partition corresponding to each gene in the supermatrix and a gamma distribution of heterogeneity of evolution rated between positions was taken into account. Two million of MCMC generations were run with MrBayes to reach congruence and 1,000 bootsrap replicates were run with RAxML to produce support values at nodes. Topologies obtained via Bayesian and ML approaches were rooted with *P. vulnus* as an outgroup. Phylogenies were visualized and edited using FigTree (http://tree.bio.ed.ac.uk/software/figtree/). The topology we obtained (Figure 6 and S5 Figure) confirms previous phylogenetic analyses [14–16].

Supplementary results on Pfam and GO annotation.

Overall, 363, 304 and 674 distinct Pfam domains were found in proteins encoded by genes with Ka / Ks ratio >1 in *M. incognita*, *M. javanica* and *M. arenaria*, respectively. These Pfam domains were used to assign GO terms using the Pfam2Go association file available at geneontology.org. A total of 177, 167 and 310 distinct GO terms were identified in proteins encoded by positively-selected genes in *M. incognita*, *M. javanica* and *M. arenaria*, respectively (S2 Table). We found 44 Molecular Function (MF), 31 Biological Process (BP) and 7 Cellular Component (CC) GO terms associated to genes under positive selection in common to the 3 apomictic species (S2 Table). The majority of MF terms were related to various different catalytic and binding activities, while BP terms were related to metabolism, DNA replication and transport. No specific CC term was particularly prominent. We used an hypergeometric test with a correction for multiple testing as implemented in FUNC [17] to detect significantly enriched GO terms in the set of positively-selected proteins as compared to the rest of proteins encoded by homeologous gene pairs. We identified 9, 8 and 8 significantly enriched MF terms (FDR <0.05) in *M. incognita*, *M. javanica* and *M. arenaria*, respectively (S2 Table), but none was common to the three species or even to 2 of the 3 species. At the BP level, 42, 23 and 68 terms were significantly enriched in Mi, Mj and Ma, respectively, with only one term (regulation of cell cycle process, GO: 0010564) in common between Mi and Ma. At the cellular component level, no term was significantly enriched in Mi while 1 was enriched in Mj and 15 in Ma and no term was common to 2 or more species. We performed the same analysis at a coarser-grain, by mapping the fine-grain GO-terms to the generic "GO-slim" ontology maintained at geneontology.org. In the MF ontology, 25 GO-slim terms were common to the 3 apomictic species and, mirroring the fine-grain GO terms, the majority of terms were related to binding or enzymatic activities. A total of 6, 2 and 0 GO-slim MF terms were found to be significantly enriched in Mi, Mj and Ma, respectively, but none was common to two or more species. Similarly, in the BP ontology, 22 GO-slim terms were common to the 3 apomictic species in the set of positively-selected proteins (S2 Table). These terms were mainly related to transport,

metabolism, stress response and cell-cycle / chromosome organization. Although 3, 0 and 12 GO-slim terms were found to be significantly enriched in Mi, Mj and Ma, respectively, none was found as commonly enriched in the 2 or more apomictic species. However, in Mi, the 3 enriched terms were "chromosome segregation (GO:0007059)", "chromosome organization" (GO:0051276) and "cell division (GO:0051301)" and mirrored the term "regulation of cell cycle process", found as commonly enriched fine-grain GO-tem between Mi and Ma. No term in the "cellular component" ontology was found to be commonly enriched in the 3 apomictic sets of proteins under positive selection.

This ensemble of results based on Gene Ontology annotation suggests that overall, genes underlying different functional categories and with proteins bearing different domains show signs of positive selection in the three apomictic root-knot nematodes.

We performed the same functional analysis on genes that showed evidence for episodic diversifying selection (EDS) based on phylogenetic analysis. We identified 123, 78 and 174 distinct Pfam domains in proteins encoded by genes under EDS in *M. incognita*, *M. javanica* and *M. arenaria*, respectively. Only 8 of these Pfam domains were common to all the 3 apomictic species, suggesting that different genes have undergone EDS independently in the different species. Based on Pfam domains, 93, 56 and 112 distinct GO terms were identified in proteins under EDS in *M. incognita*, *M. javanica* and *M. arenaria*, respectively (S3 Table). Twenty of these terms (10 MF, 7 BP and 3 CC) were common to the 3 species (S3 Table). Interestingly, 16 of these terms (9 MF, 6 BP and 1 CC) were also identified as common to the 3 species in the Ka / Ks analysis. These terms were in majority associated to binding activity (nucleic acid, RNA, GTP, ATP, nucleotide, protein) to catalytic activities (catalytic activity, metabolic process, proteolysis, metalloendopeptidase, protein kinase, phosporylation) or to transport (transmembrane transport, transport). We used a hypergeometric test with an associated FDR threshold of 0.05 to detect whether some GO-terms were significantly enriched in the genes that underwent EDS as compared to the rest of genes present in the 1,735 phylogenies of homeologous genes. No GO-term was found to be significantly enriched in the hypergeometric tests in *M. incognita*, *M. javanica* or *M. arenaria* whichever was the GO category (MF, BP or CC). We also analyzed GO-terms at a coarse-grain level, using mapping to the generic GO-Slim ontology. In the "biological process" ontology, 21, 16 and 23 GO terms were identified in *M. incognita*, *M. javanica* and *M. arenaria* EDS proteins, respectively (S3 Table). Twelve of these terms were common to the 3 apomictic species, and 9 of them were also found to be common to the three species in the Ka/ Ks analysis (S3 Table). These 9 terms were related to metabolic activities (cellular nitrogen compound metabolic process, biosynthetic process, cellular protein modification process, catabolic process), to transport (transmembrane transport, transport) or chromosome organization but none of them was significantly enriched in EDS proteins compared to the rest of the homeologous proteins analyzed. In the "molecular function" ontology, 19, 15 and 22 distinct GO terms were identified in EDS proteins of *M. incognita*, *M. javanica* and *M. arenaria*, respectively (S3 Table). A total of 11 GO terms were common to all the three species and they were mainly related to enzymatic activities (kinase, peptidase, ligase, isomerase, ATPase) or binding activities (ion, DNA, RNA), 10 of these terms were also identified as common to the 3 apomictic species

in the Ka / Ks analysis (S3 Table). However, none of these terms was significantly enriched in the set of EDS proteins as compared to the rest of homeologous proteins. Similarly, in the "cellular component" ontology, no GO-slim term was found as significantly enriched in the set of proteins under EDS.

Supplementary discussion: origin of the duplicated genome structure of apomictic Meloidogyne

Different evolutionary histories could have led to the observed genome structure made of divergent collinear regions. An ancient loss of sex in a diploid ancestor of apomictic *Meloidogyne*, followed by independent accumulation of mutations between the former paternal and maternal haplotypes could have generated the observed 8% average divergence between collinear blocks.

The Meselson-White effect hypothesis can be rejected

This so-called Meselson-White effect [18–20] can lead to the presence of two divergent genomes in one single species. Under the Meselson-White hypothesis, two sub-scenarios can be proposed. (i) Three independent long-term evolution under Meselson effect, in *M. incognita*, *M. javanica* and *M. arenaria*, after their speciation from a common sexual ancestor. This scenario would imply that duplicated regions are more similar to each other within a species than they are to any region from any other species. However, the most frequently observed topologies in our phylogenomic analysis show that genome regions tend to be more similar (and sometimes identical) across different species than they are to their other copies within the same species. Furthermore, if the three apomictic *Meloidogyne* had been experiencing Meselson effect independently for a time long enough to accumulate the ~8% intra-genomic divergence level, we would expect mitochondrial genome divergence between these species to be even higher. Indeed, in the model nematode *C. elegans*, it has been shown that the mitochondrial genome accumulates mutations 100-1,000 times more rapidly than the nuclear genome [21–23]. Yet, our mitochondrial phylogenetic analyzes shows that mitochondrial genes are almost identical in *M. incognita*, *M. arenaria* and *M. javanica* (avg. divergence ~0.17%). This confirms previous observation that these three species share virtually identical mtDNA markers [24–26] and suggests that *Mi*, *Mj*, and *Ma* share closely related or common maternal ancestors. (ii)- Another sub-scenario would be a common ancestral loss of sex, followed by long evolution under the Meselson-White effect and 3 recent speciation events (giving rise to *M. incognita*, *M. javanica* and *M. arenaria*). This sub-scenario would allow reconciliation of a high within-species genomic divergence at the nuclear level (due to long term evolution under Meselson) and nearly identical mitochondrial genomes between species (due to recent speciation events). Under this hypothesis, we would expect the different copies, representing former alleles, to form subclades with twice the same topologies (and identical to the mitochondrial topology), exactly as old shared paralogs would show. However, when duplicated regions form two or more subclades in phylogenomic analysis, these subclades generally present distinct topologies. Collinear duplicated regions within a species have thus different origins and evolutionary histories and do not originate from common ancestral allelic regions that accumulated mutations separately (*i.e.* no Meselson-White effect). Sub-scenarios i and ii differ in the age of speciation events but they both involve

long-term evolution under Meselson-White effect. Indeed, although rates of evolution in nematodes and primates cannot be directly compared, an average sequence divergence of 8% between genome copies within a species is higher than the divergence observed between the human and macaque genomes, two distinct species distant from 25 – 28 million years [27]. Thus, it can be hypothesized that a long separate accumulation of mutation would be necessary to reach such a high average sequence divergence. The presence of males is another observation arguing against an ancient loss of sexuality. Although these males do not participate in the generation of the offspring, they are morphologically close to "functional" males of sexual *Meloidogyne* species and attempts of mating with females (that did not ended up in fertilized eggs) have been reported [28]. Furthermore, sequencing of the gamete-specific gene for the major sperm protein from both meiotic and ameiotic species did not reveal any increase in evolutionary rate nor change in substitution pattern in the apomictic taxa, indicating that this locus is still under selection [26]. However, the major sperm protein might be involved in other functions unrelated with sexual reproduction or male gametogenesis. Finally, the Meselson-White effect alone would not explain the 3-5 times larger genomes of apomictic *Meloidogyne* compared to *M. hapla*.

Hybridization appears as the most likely origin

Hybridization is a tempting alternative hypothesis that could explain at the same time the presence of diverged genomic regions in the genomes, the discordance between low mitochondrial and high nuclear divergence levels as well as the observed topologies in the phylogenomic analysis. High similarity between mitochondrial genomes of the three apomictic *Meloidogyne* suggests that hybridizations with a same or very closely related maternal lineages took place. A series of observations and results converge in supporting this hypothesis. First, nuclear markers such as 18S rDNA, usually efficient to resolve phylogenetic positions, including in nematode species, fail to separate the apomictic *Meloidogyne* species (see for review Adams et al. 2009. "Molecular Taxonomy and Phylogeny" in [29]). In fact, several versions of the 18S rDNA co-exist in one single species and some versions are more similar to their cognate in another species than they are to the other copies in the same species. One clear example of this situation is observable in the 18S phylogeny published by Tigano et al. in 2005 [14], in which the different versions of 18S do not form monophyletic groups by species in apomictic *Meloidogyne*, but are rather shuffled, irrespective of the taxonomy. In the same paper, by contrast, a phylogeny performed on mtDNA markers allowed separation of the different *Meloidogyne* species and there was no observation of multiple divergent versions per species. These results suggest that there exist several versions of nuclear markers in one given apomictic species, while there is only one mtDNA version per species. Interestingly, as early as 1983, observation of heterozygous patterns of isozymes in mitotic parthenogenetic *Meloidogyne* had led to the hypothesis that these species might have undergone hybridization [30]. In 1999, based on the presence of multiple divergent ITS nuclear ribosomal markers within apomictic *Meloidogyne* despite closely related mitochondrial markers between species, it was suggested that these species had undergone hybridization from a set of closely related females mating with more diverse paternal lineages [25]. Further observations of divergent copies of nuclear markers

within a species with occasional identical copies between species, accompanied with nearly identical mitochondrial markers, have also led to similar hypothesis involving hybridization [24,26,31]. However, all these conclusions were based on one or a few nuclear and mitochondrial markers as no whole genomes were available for several apomictic *Meloidogyne* at that time.

More recently, a phylogenomic analysis of the first version of the *M. incognita* genome compared to the draft version of the *M. floridensis* genome also suggested that *M. incognita* was of hybrid origin [3]. It was also proposed that *M. floridensis* is itself a hybrid and is one of the parents of *M. incognita*. According to this hypothesis, *M. incognita* would be a double hybrid of *M. floridensis* with another, yet undetermined parent. At the mitochondrial level, our phylogenetic analysis shows that the closest known relative of *M. incognita* is *M. floridensis*, a result supporting this theory and also previously observed in other phylogenetic analyses [14–16]. Furthermore, *M. floridensis* is the only known meiotic species in a clade otherwise only comprised of mitotic species [32]. Indeed, it is thus tempting to speculate that *M. floridensis* could be the maternal donor of *M. incognita*, considering their high mitochondrial similarity. However, important additional supporting evidences are currently lacking. For instance, it is unknown whether the peculiar meiosis in *M. floridensis*, lacking a second division [33], would allow production of gametes compatible with amphimixis. It is also unclear whether *M. floridensis* is able to reproduce sexually or is obligatory parthenogenetic. Thus, another possibility would be that the donor might be another close relative of *M. floridensis* but not *M. floridensis* itself. Regardless of whether *M. floridensis* represents the maternal donor of *M. incognita*, the origins of *M. javanica* and *M. arenaria* appear to be distinct. Indeed, in mitochondrial and nuclear phylogenies, *M. floridensis* does not hold a close outgroup position relative to the three apomictic *Meloidogyne* but only to *M. incognita*. Unfortunately, the lack of contiguity of the *M. floridensis* genome prevented including this species in our phylogenomic analysis (only 12 genes forming 1 pair of duplicated regions was identified by McScanX). This prevented distinguishing WGD-derived gene copies from those resulting from other independent duplication events (tandem, proximal, dispersed).

Our phylogenomic study, includes the whole genomes of three apomictic *Meloidogyne* sequenced at deep coverage and high completeness and we compared them to the genome of the amphimictic *M. hapla*. To be as stringent as possible, we focused on genes belonging to duplicated conserved collinear regions likely to represent former parental haplotypes. Our study supports, at a whole-genome level, the hybridization hypothesis and sheds light on its evolutionary history. Phylogenies of hybrids are complex because reticulate evolution violates the assumption of a bifurcating, tree-like, evolutionary pattern. Consequently, we will likely observe different topologies for trees built from genes of either maternal or paternal origins. In this study, we separated homoeologous genomic regions (the remnant of orthologous chromosomes united into a single genome by hybridization) and reconstructed their evolutionary history. We compared history of duplicate blocks with the mtDNA phylogeny, and this allowed us to disentangle the maternal and paternal contributions to the hybrid species. The most frequently observed subclade in the phylogenies (*Mi*,(*Mj,Ma*)) is congruent with the phylogeny obtained on rDNA markers in 2002 [34]. This topology is different from the mitochondrial topology (*Ma*,(*Mi,Mj*))

and probably represents blocks with paternal contribution to the genome. The mitochondrial topology (*Ma*,(*Mi,Mj*)) is the second most frequently observed among duplicated genomic regions. Therefore, regions yielding this topology likely represent the maternal contribution to the genome of apomictic *Meloidogyne*. This topology is also congruent with the phylogeny obtained by Bayesian and ML analyses on a concatenated matrix of 47 putative orthologous genes [35]. The least frequently observed topology (*Mj*(*Ma,Mi*)) probably represents an additional and independent paternal contribution and supports the hypothesis of hybridization between one or several closely related females and different males, as first formulated in 1999 [25].

Single vs. multiple founding hybridizations

Whether, the three species each derive from three species-specific ancestral hybridization between a closely related female lineages and different males is unknown. An alternative hypothesis is that, each species does not have a single hybrid origin but that the same hybridization between the same parental species, occurred multiple times independently, leading to the same combination of genomes. Multiple independent hybridization rather than one single origin has recently been shown for the non-outcrossing hybrid *Arabidopis suecica* [36], suggesting such multiple events could be possible in *Meloidogyne* too. Interestingly, hybrids of two facultative sexual Meloidogyne have already been obtained in laboratory conditions [37]. Although the offspring is not fertile, this suggests that among all the possibilities of crossing in fields or in the wild, asexual Meloidogyne hybrids, able to produce offspring via parthenogenesis, could have emerged multiple times and new combinations could emerge in the future.

Fractionation bias

Overall, a total of 3,162 pairs of regions were identified in the genomes of the three apomictic *Meloidogyne*. We observed only ~50 cases where one block had significantly lost more genes than its counterpart in the apomictic *Meloidogyne* genomes. Thus, although this observation suggests a fragmentation bias might occur, it does not seem to be a general feature of the whole genomes. Another possibility is that hybridization events are too recent to observe a general fragmentation bias. Fragmentation bias is known in polyploid plant genomes where one homeologous genome, usually the paternal one, tends to lose more genes than the maternal genome [38].

Ploidy levels

Genome assembly sizes supported by flow cytometry estimates, CDS mapping to the genome assemblies (Figure 1) as well as abundance of Pfam domains in the asexuals compared to the sexual *M. hapla* (Figure 10), converge in bolstering polyploidy for the genomes of the asexual *Meloidogyne*. The distribution of percent identities between pairs of duplicated blocks shows a unimodal distribution, which suggests all the duplication events have taken place in a same time window. This ensemble of results suggest that *M. incognita* is triploid, *M. javanica* is tetraploid and *M. arenaria* is tetra- to (degenerate) pentaploid. Observations of chromosome numbers in these different species further support polyploidy. Determination of karyotypes and chromosome numbers is very challenging in the *Meloidogyne* because the chromosomes are dot-like and are holocentric. It is almost impossible to

differentiate and number the different chromosomes. Actually, chromosome numbers show wide variations in *Meloidogyne*, including between different populations and isolates of a same species. Nevertheless, the haploid chromosome number of the *M. hapla* strain that has been sequenced is n=16, similar to the putative ancestral haploid number of chromosomes (n=18) in *Meloidogyne* species [31,32,39]. In *M. incognita*, the most frequently observed number of chromosomes is xn=40-48 [31,40] (consistent with triploidy ~3x16). In *M. arenaria*, the most frequently observed chromosome number is xn=50-56 (~3.5x16 or 4x16 + chromosome fusion or loss) [31,39]. In *M. javanica*, the standard observed chromosome number appears to be xn=42-48 (~3x16 or ~4x16 with chromosome fusions and loss as expected in many mitotic polyploid species) [39]. These observations of high chromosome numbers that coincide with multiples of haploid ancestral chromosome numbers are consistent with polyploidy, in the apomictic *Meloidogyne*.

Supplementary methods: biological material, DNA and RNA sequencing

Meloidogyne strains

For each Meloidogyne species, we have sequenced clones from the progeny of one original single female. Eggs from *M. incognita* strain Morelos, *M. javanica* strain Avignon and *M. arenaria* strain Guadeloupe (from INRA Sophia Antipolis collection) were collected, 6 weeks after inoculation, from tomato roots (*Solanum esculentum* cv. St Pierre). Before storage at -80°C, eggs were purified from root debris by sucrose gradient centrifugations.

DNA sequencing

For *M. incognita*, because Sanger reads with different insert sizes were already available from the first version of the genome assembly [41], we produced high-throughput reads via 454 single-end as well as Illumina paired-end technologies as detailed below. For *M. arenaria* and *M. javanica*, there were no Sanger reads available, so in addition to 454 single-end and Illumina paired-end reads, we also produced 454 mate-pair libraries of different insert sizes, as explained below (see also S6 Table).

For 454 libraries, DNA was fragmented to a range of 2-4 kb or 5-10 kb using a HydroShear instrument. Fragments were end-repaired and extremities were ligated with 454 circularization adaptors. Fragments were size selected respectively to 3kb or 8kb through regular gel electrophoresis, and circularized using Cre-Lox recombination. Circular DNA was fragmented again by nebulisation. Fragments were end-repaired and ligated with library adaptors. Mate-pair libraries were amplified and purified. Single-stranded libraries were isolated, then bound to capture beads and amplified in an oil emulsion (emPCR). Libraries were then loaded on a pico-titer plate and pyrosequenced using a GS FLX according to the manufacturer’s protocol.

For Illumina libraries, DNA was sonicated using the S2 Covaris instrument (Covaris, Inc., USA). Single end libraries were prepared following Illumina’s protocol (Illumina DNA sample kit). Briefly, fragments were end-repaired, then 3’-adenylated, and Illumina adapters were added. Ligation products of 350-400 bp were gel-purified, and size-selected DNA fragments were PCR-amplified using Illumina adapter-

specific primers. Libraries were purified and then quantified using a Qubit Fluorometer (Life technologies); libraries profiles were evaluated using an Agilent 2100 bioanalyser (Agilent Technologies USA). Each library was sequenced using 101-bp paired-end read chemistry in a single flow cell on an Illumina HiSeq 2000 (Illumina, USA).

RNA sequencing

For *M. incognita*, RNA-seq data of 6 different developmental life-stages as well as for a pool of mixed stages had already been generated as part of a previous analysis [42]. For *M. arenaria* and *M. javanica* we generated libraries from nematode eggs that were sequenced on an Illumina HiSeq2000 instrument using 101 base-length read chemistry in a paired-end mode (S7 Table).

NOTE S1: DATA AVAILABILITY

All sequence data generated in this study were deposited in the EMBL-EBI European Nucleotide Archive (ENA). The genome assemblies of the 3 Meloidogyne were deposited under project accession number PRJEB8714 with sample accession numbers ERS1696677, ERS671129 and ERS671128 for *M. incognita*, *M. javanica* and *M. arenaria*, respectively. The RNA-seq reads generated for *M. incognita*, *M. javanica* and *M. arenaria* were deposited under accession numbers ERA419974, ERA419971 and ERA419973, respectively. The genome and transcriptome data are also available for download and for exploration at the Meloidogyne portal (http://meloidogyne.inra.fr).

REFERENCES

1. Humphreys-Pereira DA, Elling AA. Mitochondrial genomes of Meloidogyne chitwoodi and M. incognita (Nematoda: Tylenchina): Comparative analysis, gene order and phylogenetic relationships with other nematodes. Mol Biochem Parasitol. 2014;194: 20–32. doi:10.1016/j.molbiopara.2014.04.003

2. Humphreys-Pereira DA, Elling AA. Mitochondrial genome plasticity among species of the nematode genus Meloidogyne (Nematoda: Tylenchina). Gene. 2015;560: 173–183. doi:10.1016/j.gene.2015.01.065

3. Lunt DH, Kumar S, Koutsovoulos G, Blaxter ML. The complex hybrid origins of the root knot nematodes revealed through comparative genomics. PeerJ. 2014;2: e356. doi:10.7717/peerj.356

4. Opperman CH, Bird DM, Williamson VM, Rokhsar DS, Burke M, Cohn J, et al. Sequence and genetic map of Meloidogyne hapla: A compact nematode genome for plant parasitism. Proc Natl Acad Sci U A. 2008;105: 14802–7.

5. Sun L, Zhuo K, Lin B, Wang H, Liao J. The Complete Mitochondrial Genome of Meloidogyne graminicola (Tylenchina): A Unique Gene Arrangement and Its Phylogenetic Implications. PLoS ONE. 2014;9: e98558. doi:10.1371/journal.pone.0098558

6. Besnard G, Jühling F, Chapuis É, Zedane L, Lhuillier É, Mateille T, et al. Fast assembly of the mitochondrial genome of a plant parasitic nematode (Meloidogyne graminicola) using next generation sequencing. C R Biol. 2014;337: 295–301. doi:10.1016/j.crvi.2014.03.003

7. Sultana T, Kim J, Lee S-H, Han H, Kim S, Min G-S, et al. Comparative analysis of complete mitochondrial genome sequences confirms independent origins of plant-parasitic nematodes. BMC Evol Biol. 2013;13: 12. doi:10.1186/1471-2148-13-12

8. Katoh K, Standley DM. MAFFT multiple sequence alignment software version 7: improvements in performance and usability. Mol Biol Evol. 2013;30: 772–80. doi:10.1093/molbev/mst010

9. Suyama M, Torrents D, Bork P. PAL2NAL: robust conversion of protein sequence alignments into the corresponding codon alignments. Nucleic Acids Res. 2006;34: W609–W612. doi:10.1093/nar/gkl315

10. Capella-Gutiérrez S, Silla-Martínez JM, Gabaldón T. trimAl: a tool for automated alignment trimming in large-scale phylogenetic analyses. Bioinformatics. 2009;25: 1972–1973. doi:10.1093/bioinformatics/btp348

11. Posada D. jModelTest: Phylogenetic Model Averaging. Mol Biol Evol. 2008;25: 1253–1256. doi:10.1093/molbev/msn083

12. Ronquist F, Teslenko M, Mark P van der, Ayres DL, Darling A, Höhna S, et al. MrBayes 3.2: Efficient Bayesian Phylogenetic Inference and Model Choice across a Large Model Space. Syst Biol. 2012; sys029. doi:10.1093/sysbio/sys029

13. Stamatakis A. RAxML Version 8: A tool for Phylogenetic Analysis and Post-Analysis of Large Phylogenies. Bioinformatics. 2014; btu033. doi:10.1093/bioinformatics/btu033

14. Tigano MS, Carneiro RM, Jeyaprakash A, Dickson DW, Adams BJ. Phylogeny of Meloidogyne spp. based on 18S rDNA and the intergenic region of mitochondrial DNA sequences. Nematology. 2005;7: 851–862.

15. Adams BJ, Dillman AR, Finlinson C. Molecular Taxonomy and Phylogeny. In: Perry RN, Moens M, Starr JL, editors. Root-Knot Nematodes. 2009. pp. 119–138.

16. García LE, Sánchez-Puerta MV. Comparative and Evolutionary Analyses of Meloidogyne spp. Based on Mitochondrial Genome Sequences. PLoS ONE. 2015;10: e0121142. doi:10.1371/journal.pone.0121142

17. Prüfer K, Muetzel B, Do H-H, Weiss G, Khaitovich P, Rahm E, et al. FUNC: a package for detecting significant associations between gene sets and ontological annotations. BMC Bioinformatics. 2007;8: 41.

18. White MJD. Animal cytology and evolution. Cambridge, UK: Cambridge University Press.; 1977.

19. Birky CW. Heterozygosity, heteromorphy, and phylogenetic trees in asexual eukaryotes. Genetics. 1996;144: 427–37.

20. Mark Welch D, Meselson M. Evidence for the evolution of bdelloid rotifers without sexual reproduction or genetic exchange. Science. 2000;288: 1211–5.

21. Denver DR, Morris K, Lynch M, Vassilieva LL, Thomas WK. High direct estimate of the mutation rate in the mitochondrial genome of Caenorhabditis elegans. Science. 2000;289: 2342–4.

22. Denver DR, Morris K, Lynch M, Thomas WK. High mutation rate and predominance of insertions in the Caenorhabditis elegans nuclear genome. Nature. 2004;430: 679–682. doi:10.1038/nature02697

23. Denver DR, Dolan PC, Wilhelm LJ, Sung W, Lucas-Lledó JI, Howe DK, et al. A genome-wide view of Caenorhabditis elegans base-substitution mutation processes. Proc Natl Acad Sci. 2009;106: 16310–16314. doi:10.1073/pnas.0904895106

24. Fargette M, Berthier K, Richaud M, Lollier V, Franck P, Hernandez A, et al. Crosses prior to parthenogenesis explain the current genetic diversity of tropical plant-parasitic Meloidogyne species (Nematoda: Tylenchida). Infect Genet Evol. 2010;10: 806–813. doi:10.1016/j.meegid.2009.04.013

25. Hugall A, Stanton J, Moritz C. Reticulate evolution and the origins of ribosomal internal transcribed spacer diversity in apomictic Meloidogyne. Mol Biol Evol. 1999;16: 157–164.

26. Lunt DH. Genetic tests of ancient asexuality in Root Knot Nematodes reveal recent hybrid origins. BMC Evol Biol. 2008;8: 194. doi:10.1186/1471-2148-8-194

27. Rogers J, Gibbs RA. Comparative primate genomics: emerging patterns of genome content and dynamics. Nat Rev Genet. 2014;15: 347–359. doi:10.1038/nrg3707

28. Triantaphyllou AC. Oogenesis and the Chromosomes of the Parthenogenic Root-knot Nematode Meloidogyne incognita. J Nematol. 1981;13: 95–104.

29. Perry RN, Moens M, Starr JL, editors. Root-knot nematodes. Cambridge, MA: CABI North American Office; 2009.

30. Dalmasso A, Berge JB. Enzyme polymorphism and the concept of parthenogenetic species, exemplified by Meloidogyne.. Stone AR, Platt HM, Khalil LF, editors. Concepts Nematode Syst Proc Intern Symp Camb Univ 2-4 Sept 1981 Syst Assoc Spec Vol 22. 1983; 187–196.

31. Castagnone-Sereno P. Genetic variability and adaptive evolution in parthenogenetic root-knot nematodes. Heredity. 2006;96: 282–9.

32. Castagnone-Sereno P, Danchin EG, Perfus-Barbeoch L, Abad P. Diversity and Evolution of

Root-Knot Nematodes, Genus Meloidogyne: New Insights from the Genomic Era. Annu Rev Phytopathol. 2013;51: 130517121443006. doi:10.1146/annurev-phyto-082712-102300

33. Handoo ZA, Nyczepir AP, Esmenjaud D, van der Beek JG, Castagnone-Sereno P, Carta LK, et al. Morphological, Molecular, and Differential-Host Characterization of Meloidogyne floridensis n. sp. (Nematoda: Meloidogynidae), a Root-Knot Nematode Parasitizing Peach in Florida. J Nematol. 2004;36: 20–35.

34. De Ley IT, De Ley P, Vierstraete A, Karssen G, Moens M, Vanfleteren J. Phylogenetic Analyses of Meloidogyne Small Subunit rDNA. J Nematol. 2002;34: 319–27.

35. Scholl EH, Bird DM. Resolving tylenchid evolutionary relationships through multiple gene analysis derived from EST data. Mol Phylogenet Evol. 2005;36: 536–545. doi:10.1016/j.ympev.2005.03.016

36. Novikova PY, Tsuchimatsu T, Simon S, Nizhynska V, Voronin V, Burns R, et al. Genome Sequencing Reveals the Origin of the Allotetraploid Arabidopsis suecica. Mol Biol Evol. 2017;34: 957–968. doi:10.1093/molbev/msw299

37. van der Beek JG, Karssen G. Interspecific Hybridization of Meiotic Parthenogenetic Meloidogyne chitwoodi and M. fallax. Phytopathology. 1997;87: 1061–6. doi:10.1094/PHYTO.1997.87.10.1061

38. Murat F, Zhang R, Guizard S, Flores R, Armero A, Pont C, et al. Shared Subgenome Dominance Following Polyploidization Explains Grass Genome Evolutionary Plasticity from a Seven Protochromosome Ancestor with 16K Protogenes. Genome Biol Evol. 2014;6: 12–33. doi:10.1093/gbe/evt200

39. Triantaphyllou AC. Cytogenetics, cytotaxonomy and phylogeny of root-knot nematodes. In: Sasser JN, Carter CC, editors. An advance treatise on Meloidogyne. Raleigh, USA: North Carolina State University Graphics; 1985. pp. 113–26.

40. Triantaphyllou AC. Oogenesis and the chromosomes of the parthenogenetic Root Knot Nematode Meloidogyne incognita. J Nematol. 1981; 95–104.

41. Abad P, Gouzy J, Aury J-M, Castagnone-Sereno P, Danchin EGJ, Deleury E, et al. Genome sequence of the metazoan plant-parasitic nematode Meloidogyne incognita. Nat Biotechnol. 2008;26: 909–915. doi:10.1038/nbt.1482

42. Danchin EGJ, Arguel M-J, Campan-Fournier A, Perfus-Barbeoch L, Magliano M, Rosso M-N, et al. Identification of Novel Target Genes for Safer and More Specific Control of Root-Knot Nematodes from a Pan-Genome Mining. PLoS Pathog. 2013;9: e1003745. doi:10.1371/journal.ppat.1003745
