# Supplementary material for: Hybridization and polyploidy enable genomic plasticity without sex in the most devastating plant-parasitic nematodes
Source: PLoS Genet. 2017 Jun 8;13(6):e1006777. doi: 10.1371/journal.pgen.1006777 (PMC5465968; doi:10.1371/journal.pgen.1006777)
Supplement: S2 Text — (PDF) [file pgen.1006777.s010.pdf]

```
#!/usr/bin/python
from __future__ import division
from Bio import SeqIO
import sys
import random
import shlex
import os

'''
README
author: romain blanc-mathieu, INRA sophia antipolis, FRANCE.
aim1: get the genomics sequences corresponding to within genome
syntenic blocks (as defined by MCScanX).
aim2: Nucmer pairwise alignment of block genomic sequences and compute
percent identity between the two.
command: extractColBlock.py McScanX.collinearity genomeAssembly.fna
genomeAnnotation.gff3
dependencies: SeqIO from Bio python and nucmer, delta-filter and dna-
diff from the Mummer package.
'''

if len(sys.argv) <= 1:
        print 'enter name of collinear file'
        sys.exit(1)
f_col=open(sys.argv[1], 'r')

if len(sys.argv) <= 2:
        print 'enter name of genome fasta'
        sys.exit(1)
f_fasta=open(sys.argv[2], 'r')

if len(sys.argv) <= 3:
        print 'enter name of gff3 file'
        sys.exit(1)
f_gff3=open(sys.argv[3], 'r')

print "this codes reads into:"
print "...genome collinearity MCScanX files"
print "...genome scaffolds fasta"
print "...original genome gff3"
print "it extracts sequences of collinear blocks "
print "let s beging...\n\n"
print "load list of list of genes per collinear block (a list for each
homeologs in a collinear block"
l1=[]
l2=[]
L1=[]
L2=[]
start='no'
for line in f_col:
     if line[3:12]=="Alignment":
          if start=="yes":
```

```
                L1.append(l1)
                L2.append(l2)
            l1=[]
            l2=[]

        elif line[0]!="#":
            tmp=line.split("\t")
            l1.append(tmp[1])
            l2.append(tmp[2])
            start="yes"
L1.append(l1)
L2.append(l2)
f_col.close()

print "Now the script reads the gff3 file"
#It creates 3 dicos. All with genes name as key.
#The first one gives the posi of the gene on its scaffol,
#The second one gives the posf
# and the 3rd one gives the scaffold name
dicoPosi={}
dicoPosf={}
dicoScaf={}
for line in f_gff3:
        if line[0]!="#":
            #print line
            tmp=line.split("\t")
            if tmp[2]=="gene":
                tmp2=tmp[8].split(";")
                #print tmp2
                #geneName=tmp2[1][5:14]
                geneName=tmp2[0][8::]
                #print geneName
                dicoPosi[geneName]=int(tmp[3])
                dicoPosf[geneName]=int(tmp[4])
                dicoScaf[geneName]=tmp[0]
                #exit()
f_gff3.close()

print "Now load the posi posf and scaffold name of each pair of
collinear blocks"
bloc_1_Posi=[]
bloc_1_Posf=[]
bloc_1_Scaf=[]
for blo in L1:
        tmpi=[]
        tmpf=[]
        for ge in blo:
            tmpi.append(dicoPosi[ge])
            tmpf.append(dicoPosf[ge])
        bloc_1_Posi.append(min(tmpi))
        bloc_1_Posf.append(max(tmpf))
        bloc_1_Scaf.append(dicoScaf[ge])
```

```
bloc_2_Posi=[]
bloc_2_Posf=[]
bloc_2_Scaf=[]
for blo in L2:
      tmpi=[]
      tmpf=[]
      for ge in blo:
            tmpi.append(dicoPosi[ge])
            tmpf.append(dicoPosf[ge])
      bloc_2_Posi.append(min(tmpi))
      bloc_2_Posf.append(max(tmpf))
      bloc_2_Scaf.append(dicoScaf[ge])

print bloc_1_Posi
print bloc_1_Posf
print bloc_1_Scaf

print "there is %d collinear blocs" % len(bloc_1_Scaf)

print "Now read in the genome assembly fasta file (scaffold) to extract
sequences and do nucmer pairwise alignment"
found=0
lg1=[]
lg2=[]
TotalLength=[]
AvgLength=[]
AvgIdentity=[]
def specialParsingNucmer(nucline):
      value=''
      get=0
      for c in nucline[12::]:
            if c!=" ":
                  value=value+c
                  get=1
            if c==" " and get==1:
                  break
      return value

for numBlo in range(len(bloc_1_Posf)):
      for seq_record in SeqIO.parse(f_fasta, "fasta"):
            if seq_record.name==bloc_1_Scaf[numBlo]:
                  fout_sequ=open('bloc%d_1st_inPair' %  numBlo, 'w' )
                  fout_sequ.write(">%s_%d_%d\n%s\n" %
(bloc_1_Scaf[numBlo], bloc_1_Posi[numBlo], bloc_1_Posf[numBlo],
seq_record.seq[bloc_1_Posi[numBlo]:bloc_1_Posf[numBlo]]))
                  fout_sequ.close()

      lg1.append(len(seq_record.seq[bloc_1_Posi[numBlo]:bloc_1_Posf[num
Blo]])+1)
                  found+=1
            if seq_record.name==bloc_2_Scaf[numBlo]:
```

```
                fout_sequ=open('bloc%d_2nd_inPair' %  numBlo, 'w' )
                fout_sequ.write(">%s_%d_%d\n%s\n" %
(bloc_2_Scaf[numBlo], bloc_2_Posi[numBlo], bloc_2_Posf[numBlo],
seq_record.seq[bloc_2_Posi[numBlo]:bloc_2_Posf[numBlo]]))
                fout_sequ.close()

     lg2.append(len(seq_record.seq[bloc_2_Posi[numBlo]:bloc_2_Posf[num
Blo]])+1)
                found+=1
          if found==2:
                os.system("sed 's/N//g' bloc%d_1st_inPair> tmp1" %
numBlo)
                os.system("sed 's/N//g' bloc%d_2nd_inPair> tmp2" %
numBlo)

                os.system("mv tmp1 bloc%d_1st_inPair" % numBlo)
                os.system("mv tmp2 bloc%d_2nd_inPair" % numBlo)

                os.system("nucmer bloc%d_1st_inPair bloc%d_2nd_inPair
> tmp.txt" % (numBlo, numBlo))
                os.system("delta-filter -l 50 out.delta >
out.flt.delta")
                os.system("dnadiff -d out.flt.delta > tmp.txt")
                os.system("grep 1-to-1 -A 4 out.report > tmp.txt")
                for line in open("tmp.txt", 'r'):
                     if line[0:11]=="TotalLength":

     TotalLength.append(int(specialParsingNucmer(line)))
                     if line[0:9]=="AvgLength":

     AvgLength.append(float(specialParsingNucmer(line)))
                     if line[0:11]=="AvgIdentity":

     AvgIdentity.append(float(specialParsingNucmer(line)))

                #os.system("rm out* tmp.txt")
                f_fasta.seek(0)
                found=0
                break
f_fasta.close()
f_table_out=open("table_nucmer_dnadiff_summary_perBloc.txt", 'w')
f_table_out.write("lg1\tlg2\talglg1\tfractionalginedlg1\tavgalglg\tavgi
d\n")
#Write out results:
for i in range(len(AvgIdentity)):
     f_table_out.write("%d\t%d\t%d\t%f\t%f\t%f\n" % (lg1[i], lg2[i],
TotalLength[i], TotalLength[i]/lg1[i], AvgLength[i], AvgIdentity[i]))


print "\nfinito ! "
print "%d and %d collinear block have been treated\n" % (len(L1),
len(L2))
```
