# Supplementary material for: Hybridization and polyploidy enable genomic plasticity without sex in the most devastating plant-parasitic nematodes
Source: PLoS Genet. 2017 Jun 8;13(6):e1006777. doi: 10.1371/journal.pgen.1006777 (PMC5465968; doi:10.1371/journal.pgen.1006777)
Supplement: S1 Table — (PDF) [file pgen.1006777.s011.pdf]

S1 Table: Source and accession numbers of mitochondrial sequences

Species	Suffix	Source	Accession
Meloidogyne incognita	D	Genbank	NC_024097
	L	EMBL ENA	PRJEB8714
Meloidogyne javanica	D	Genbank	KP202352
	L	EMBL ENA	PRJEB8714
Meloidogyne arenaria	D	Genbank	KP202350
	L	EMBL ENA	PRJEB8714
Meloidogyne floridensis	D	Genbank	CCDZ00000000.1
Meloidogyne enterolobii	D	Genbank	KP202351
Meloidogyne hapla	D	Genbank	ABLG00000000.1
Meloidogyne chitwoodi	D	Genbank	NC_024096
Meloidogyne graminicola	D	Genbank	KJ139963
	D'	Genbank	NC_024275
Pratylenchus vulnus	D	Genbank	NC_020434
