# Supplementary material for: Hybridization and polyploidy enable genomic plasticity without sex in the most devastating plant-parasitic nematodes
Source: PLoS Genet. 2017 Jun 8;13(6):e1006777. doi: 10.1371/journal.pgen.1006777 (PMC5465968; doi:10.1371/journal.pgen.1006777)
Supplement: S4 Table — (PDF) [file pgen.1006777.s014.pdf]

S4 Table

Pfam - TE	Desc	Long-desc	*M. inc*	*M. jav*	*M. are*	*M. hap*
PF01359	Transposase_1	Transposase (partial DDE domain)	1	15	13	1
PF12762	DDE_Tnp_IS1595	ISXO2-like transposase domain	10	71	109	7
PF13518	HTH_28	Helix-turn-helix domain	0	2	5	0
PF13358	DDE_3	DDE superfamily endonuclease	13	61	61	0
PF14529	Exo_endo_phos_2	Endonuclease-reverse transcriptase	33	140	160	16
PF01527	HTH_Tnp_1	Transposase	0	2	2	0
PF05380	Peptidase_A17	Pao retrotransposon peptidase	71	271	285	14
PF03732	Retrotrans_gag	Retrotransposon gag protein	3	9	16	3
PF00665	rve	Integrase core domain	205	599	689	59
PF00078	RVT_1	Reverse transcriptase (RNA-dependent DNA polymerase)	205	593	669	69
PF13975	gag-asp_proteas	gag-polyprotein putative aspartyl protease	1	3	6	0
PF02892	zf-BED	BED zinc finger	39	85	102	25
PF03564	DUF1759	Protein of unknown function (DUF 1759)	205	424	465	20
PF04937	DUF659	Protein of unknown function (DUF 659)	1	5	1	0
PF00385	Chromo	Chromo (CHRromatin Organisation MOdifier) domain	88	163	191	29
PF00098	zf-CCHC	Zinc knuckle	134	242	295	42
PF05699	Dimer_Tnp_hAT	hAT family C-terminal dimerisation region	94	135	225	24
PF01498	HTH_Tnp_Tc3_2	Transposase	4	9	8	1
PF00692	dUTPase	dUTPase	4	9	7	3
PF03184	DDE_1	DDE superfamily endonuclease	19	32	37	7
PF05485	THAP	THAP domain	3	6	6	0
PF03221	HTH_Tnp_Tc5	Tc5 transposase DNA-binding domain	27	40	46	10
PF00077	RVP	Retroviral aspartyl protease	38	51	66	15
PF04218	CENP-B_N	CENP-B N-terminal DNA-binding domain	10	16	15	7
PF07572	BCNT	Bucentaur or craniofacial development	3	5	4	1
PF00075	RNase_H	RNase H	5	6	7	2
PF03276	Gag_spuma	Spumavirus gag protein	0	1	0	0
PF01393	Chromo_shadow	Chromo shadow domain	2	3	2	0
**Total**			1218	2998	3492	355
