# Supplementary material for: Hybridization and polyploidy enable genomic plasticity without sex in the most devastating plant-parasitic nematodes
Source: PLoS Genet. 2017 Jun 8;13(6):e1006777. doi: 10.1371/journal.pgen.1006777 (PMC5465968; doi:10.1371/journal.pgen.1006777)
Supplement: S5 Table — (PDF) [file pgen.1006777.s015.pdf]

S5 Table. Statistics of genome assemblies.

	Meloidogyne incognita	*Meloidogyne arenaria*	*Meloidogyne javanica*
Assembly software	Mira + Sspace	Mira + Sspace	Mira + Sspace
# of contigs	13,148	28,641	33,008
Cumulative size (bp)	180,221,179	256,294,081	235,523,531
Contig N50 bp (L50)	32,388 (1,440)	14,448 (5,017)	9,986 (6,981)
# of scaffolds	12,091	26,196	31,341
Cumulative size (bp)	183,531,997	258,067,405	235,798,407
Scaffold N50 bp (L50)	38,588 (1,209)	16,462 (4,438)	10,388 (6,741)
Number of N's	3,319,814 (1.8%)	1,803,958 (0.69%)	312,237 (0.13%)
GC percent	29.75%	29.97%	29.96%
Accession Numbers	PRJEB8714 (ERS1696677)	PRJEB8714 (ERS671129)	PRJEB8714 (ERS671128)
