# Supplementary material for: Hybridization and polyploidy enable genomic plasticity without sex in the most devastating plant-parasitic nematodes
Source: PLoS Genet. 2017 Jun 8;13(6):e1006777. doi: 10.1371/journal.pgen.1006777 (PMC5465968; doi:10.1371/journal.pgen.1006777)
Supplement: S6 Table — (PDF) [file pgen.1006777.s016.pdf]

S6 Table. Statistics for different sequencing technologies performed in this study for the three genomes.

Species	Sequencing technology	Library preparation	Number of reads	Number of bp	Coverage
Meloidogyne incognita	Sanger*	Paired-end + BAC-ends (3kb + 40kb)	994,997	~410,000,000	~2X
	Roche/454 Titanium	Single end reads	8,702,024	2,964,881,774	~16X
	Illumina HiSeq2000	Paired-end reads (2x101bp)	156,748,942	15,831,643,142	~82X
Meloidogyne arenaria	Roche/454 GSFLX+	Single end reads	5,122,701	2,482,541,174	~10X
	Roche/454 GSFLX+	Mate-Pair 3Kb	1,002,580	255,992,163	~1X
	Roche/454 GSFLX+	Mate-Pair 8Kb	2,456,652	767,186,841	~3X
	Illumina HiSeq2000	Paired-end reads (2x101bp)	178,179,864	17,429,987,689	~68X
Meloidogyne javanica	Roche/454 GSFLX+	Single end reads	5,384,705	2,292,836,886	~10X
	Roche/454 Titanium	Mate-Pair 3Kb	1,362,980	355,082,359	~1X
	Illumina HiSeq2000	Paired-end reads (2x101bp)	147,863,372	14,121,114,101	~60X

* material produced for the first version of the *M. incognita* genome [1]

1. Abad P, Gouzy J, Aury J-M, Castagnone-Sereno P, Danchin EGJ, Deleury E, et al. Genome sequence of the metazoan plant-parasitic nematode Meloidogyne incognita. Nat Biotechnol. 2008;26: 909–915. doi:10.1038/nbt.1482
