# Supplementary material for: Hybridization and polyploidy enable genomic plasticity without sex in the most devastating plant-parasitic nematodes
Source: PLoS Genet. 2017 Jun 8;13(6):e1006777. doi: 10.1371/journal.pgen.1006777 (PMC5465968; doi:10.1371/journal.pgen.1006777)
Supplement: S7 Table — (PDF) [file pgen.1006777.s017.pdf]

S7 Table. Statistics for sequencing technologies performed for transcriptomic support for gene prediction.

Species	Sequencing technology	Library preparation	Description	Number of reads	Number of bp	Accession Number
*M. incognita**	Illumina GAIIx	Single-end reads (76nt)	Egg Transcriptome	19,077,883	1,388,062,528	ERR790022
			Female Transcriptome	13,240,851	927,553,054	ERR790025
			Male Transcriptome	25,368,782	1,777,456,973	ERR790023
			Parasitic juvenile J3-J4 Transcriptome	3,112,384	218,791,109	ERR790024
			Parasitic juvenile Group2 Transcriptome	27,533,689	2,005,818,633	ERR790026
			Parasitic juvenile Group3 Transcriptome	7,333,207	530,034,047	ERR790029
			Mix of egg, J2, J3-J4, female, male	8,826,463	643,493,089	ERR790021
	Illumina HiSeq2000	Paired-end reads (2x101bp)	Preparasitic J2.1 Transcriptome	55,636,452	5,083,772,246	ERR790028
			Preparasitic J2.2 Transcriptome	39,683,554	3,602,903,802	ERR790027
M. arenaria	Illumina HiSeq2000	Paired-end reads (2x101bp)	Egg and J2 Transcriptome	61,704,102	5,623,317,775	ERR790020
M. javanica	Illumina HiSeq2000	Paired-end reads (2x101bp)	Egg and J2 Transcriptome	72,253,538	6,552,638,438	ERR790021

* RNA-seq data produced as part of a previous analysis [1]

1. Danchin EGJ, Arguel M-J, Campan-Fournier A, Perfus-Barbeoch L, Magliano M, Rosso M-N, et al. Identification of Novel Target Genes for Safer and More Specific Control of Root-Knot Nematodes from a Pan-Genome Mining. PLoS Pathog. 2013;9: e1003745. doi:10.1371/journal.ppat.1003745
